# Semiconductor Porous Hydrogen-Bonded Organic Frameworks Based on Tetrathiafulvalene Derivatives

**DOI:** 10.1021/jacs.2c01957

**Published:** 2022-05-16

**Authors:** María Vicent-Morales, María Esteve-Rochina, Joaquín Calbo, Enrique Ortí, Iñigo J. Vitórica-Yrezábal, Guillermo Mínguez Espallargas

**Affiliations:** † Instituto de Ciencia Molecular (ICMol), Universidad de Valencia, c/ Catedrático José Beltrán, 2, Paterna 46980, Spain; ‡ School of Chemistry, University of Manchester, Oxford Road, Manchester M13 9PL, U.K.

## Abstract

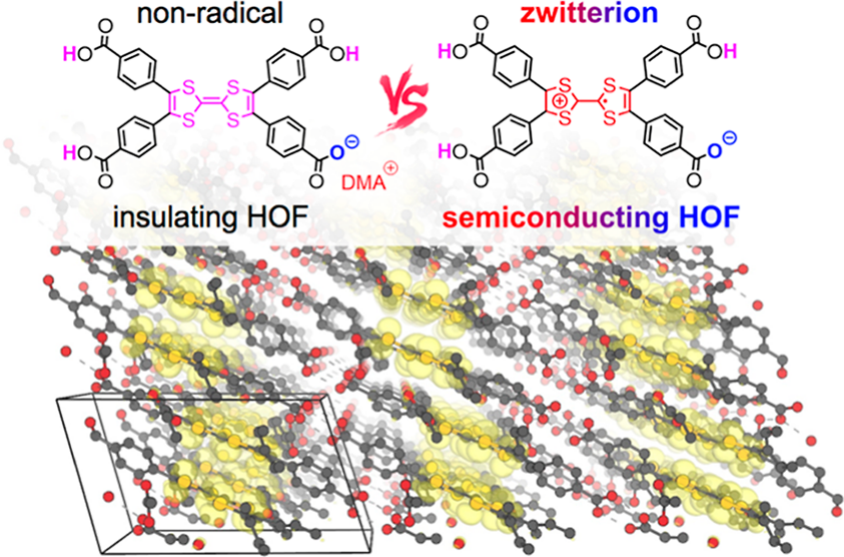

Herein, we report on the use of tetrathiavulvalene-tetrabenzoic acid, H_4_TTFTB, to engender semiconductivity in porous hydrogen-bonded organic frameworks (HOFs). By tuning the synthetic conditions, three different polymorphs have been obtained, denoted **MUV-20a**, **MUV-20b**, and **MUV-21**, all of them presenting open structures (22, 15, and 27%, respectively) and suitable TTF stacking for efficient orbital overlap. Whereas **MUV-21** collapses during the activation process, **MUV-20a** and **MUV-20b** offer high stability evacuation, with a CO_2_ sorption capacity of 1.91 and 1.71 mmol g^–1^, respectively, at 10 °C and 6 bar. Interestingly, both **MUV-20a** and **MUV-20b** present a zwitterionic character with a positively charged TTF core and a negatively charged carboxylate group. First-principles calculations predict the emergence of remarkable charge transport by means of a through-space hopping mechanism fostered by an efficient TTF π–π stacking and the spontaneous formation of persistent charge carriers in the form of radical TTF^•+^ units. Transport measurements confirm the efficient charge transport in zwitterionic **MUV-20a** and **MUV-20b** with no need for postsynthetic treatment (e.g., electrochemical oxidation or doping), demonstrating the semiconductor nature of these HOFs with record experimental conductivities of 6.07 × 10^–7^ (**MUV-20a**) and 1.35 × 10^–6^ S cm^–1^ (**MUV-20b**).

## Introduction

The design of materials combining electrical conductivity and porosity is an interesting but challenging topic that has gained exponential attention in recent years for exploitation in next-generation applications.^1,2^ Despite being usually considered insulating materials, metal–organic frameworks (MOFs)^3^ and covalent organic frameworks (COFs)^4^ are porous crystalline structures that have proven to be efficient for the transport of electrical charge with a suitable chemical design.^5−7^ This has been achieved through organic linkers with an extended π-conjugation,^8,9^ via the existence of a π–π stacking through-space pathway,^10−12^ or with the incorporation of electroactive guest molecules within the pores of these crystalline materials.^13^ Most relevant strategies to boost conductivity in porous materials rely on charge carrier formation, for example, through partial ligand oxidation upon iodine doping or partial metal-node oxidation/reduction in mixed-valence frameworks.^14^ However, these approaches imply either a trade-off where porosity and available surface area are decreased or a very limited versatility to access mixed-valence states (e.g., Fe(II)/Fe(III)-based frameworks), respectively. Moreover, the implementation of MOFs/COFs in efficient devices is not trivial,^6^ although different approaches have been successfully accomplished in this regard.^15−18^

Hydrogen-bonded organic frameworks, or HOFs,^19−21^ have appeared as an alternative type of porous molecular-based crystalline materials that are self-assembled through H-bonding interactions. Similar to MOFs and COFs, HOFs have been used in gas storage,^22^ separation,^23^ encapsulation,^24^ or proton conductivity,^25^ among others.^26^ However, due to the lack of formation of strong coordination bonds (as in MOFs) or covalent bonds (as in COFs), HOFs can be synthesized under mild conditions,^27^ which facilitates their processing, one of the major drawbacks of MOFs and COFs.^16,20^ Thus, the preparation of conductive HOFs appears as an intuitive solution for the problematic synthetic methods available for MOFs and COFs.

Tetrathiafulvalene (TTF) derivatives are a well-known family of molecular-based conductors extensively studied in the field of molecular electronics,^28,29^ as well as for being used in the preparation of conductive MOFs^30^ and COFs.^31^ In these systems, in addition to a suitable orbital overlap, it is essential to obtain the radical cation TTF^•+^ to engender the charge carrier species. This usually implies the oxidation of the material, typically with I_2_ or Br_2_, which results in a decreased sorption capacity due to the incorporation of the reduced I_3_^–^ or Br^–^ species.^32^

Tetrathiafulvalene-tetrabenzoic acid, in short H_4_TTFTB, is a well-known ligand that has been used by us^33−37^ and others^38,39^ in the preparation of MOFs and COFs. This ligand has also been employed in the construction of a HOF with limited stability, which collapses upon desolvation,^40^ and more recently, it has shown to be involved in single-crystal-to-single-crystal transformations.^41^ A very recent development in TTF-based HOFs has been shown by Farha and co-workers, who have prepared a HOF with a naphthalene derivative of TTF, which, upon oxidation with I_2_, becomes a semiconductor (conductivity of 6.0 × 10^–7^ S cm^–1^).^42^

Herein, we present the use of the H_4_TTFTB molecule to prepare two new porous HOFs, in which the building block ligand has a zwitterionic character. These materials, denoted **MUV-20a** and **MUV-20b**, do not require additional doping for electrical charge transport, resulting in two porous conducting HOFs while preserving their crystalline porosity. The semiconducting properties of **MUV-20a** and **MUV-20b** stand record within the HOF materials and contrast with those exhibited by a non-zwitterionic HOF based on the same ligand, denoted **MUV-21**, which behaves as an insulator despite the suitable stacking of the TTF units. Zwitterionic polaron formation within an organic crystal therefore emerges as an new potential strategy to boost conductivity in porous materials.

## Results and Discussion

**Figure 1:**
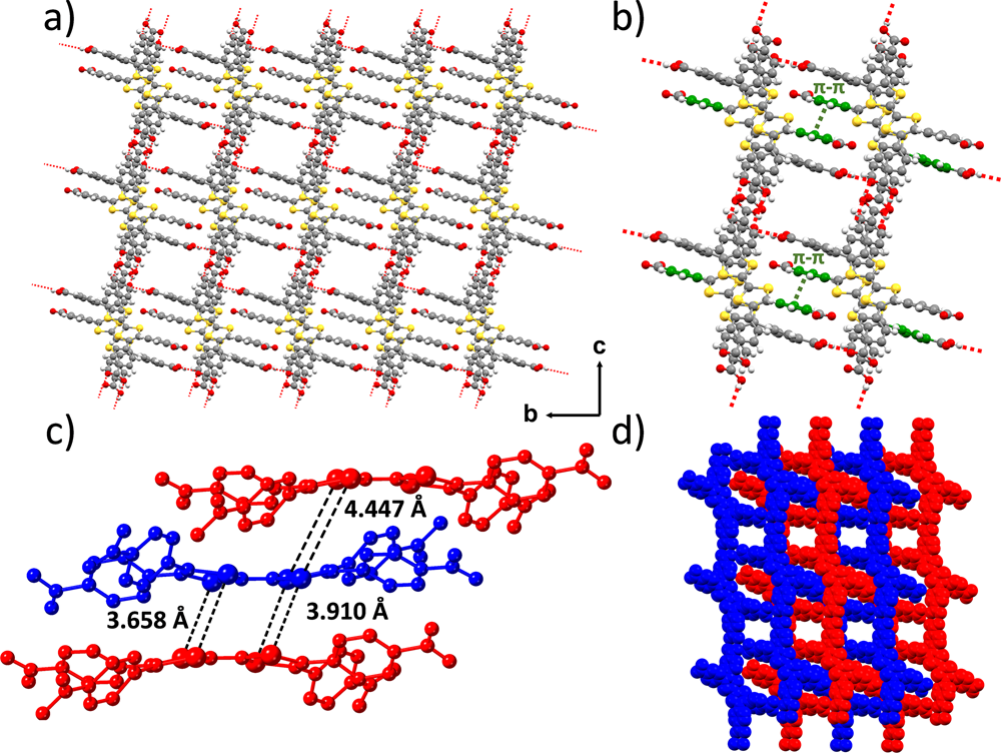
(a) Crystal structure of **MUV-20a** showing the channels along the *a*-axis. (b) Microporous channel showing the hydrogen-bond interactions (in red) and the π–π stacking (in green) between benzene groups. (c) Distances between sulfur atoms of TTF units in adjacent layers. (d) Different layers showing the interpenetration along the *a*-axis. The THF molecules present in the pores have been omitted for clarity.

**Figure 2:**
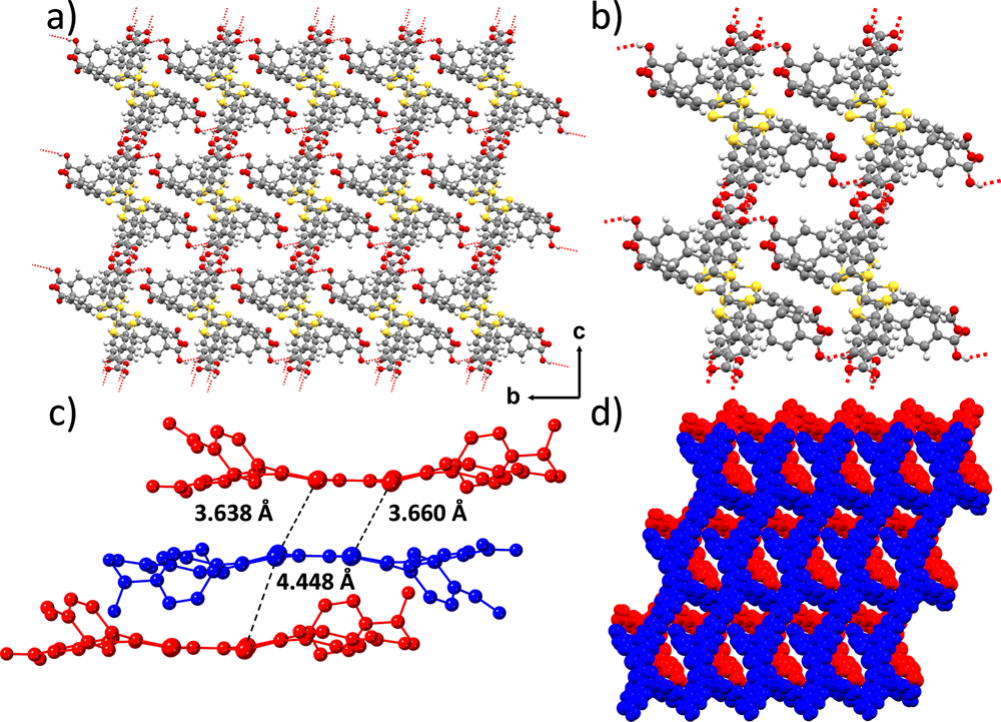
(a) Crystal structure of **MUV-20b** showing the channels along the *a*-axis. (b) Microporous channel showing the hydrogen-bond interactions in red. (c) Distances between sulfur atoms of TTF units in adjacent layers. (d) Different layers showing the non-interpenetration of the structure along the *a*-axis. The ether molecules present in the pores have been omitted for clarity.

**Figure 3:**
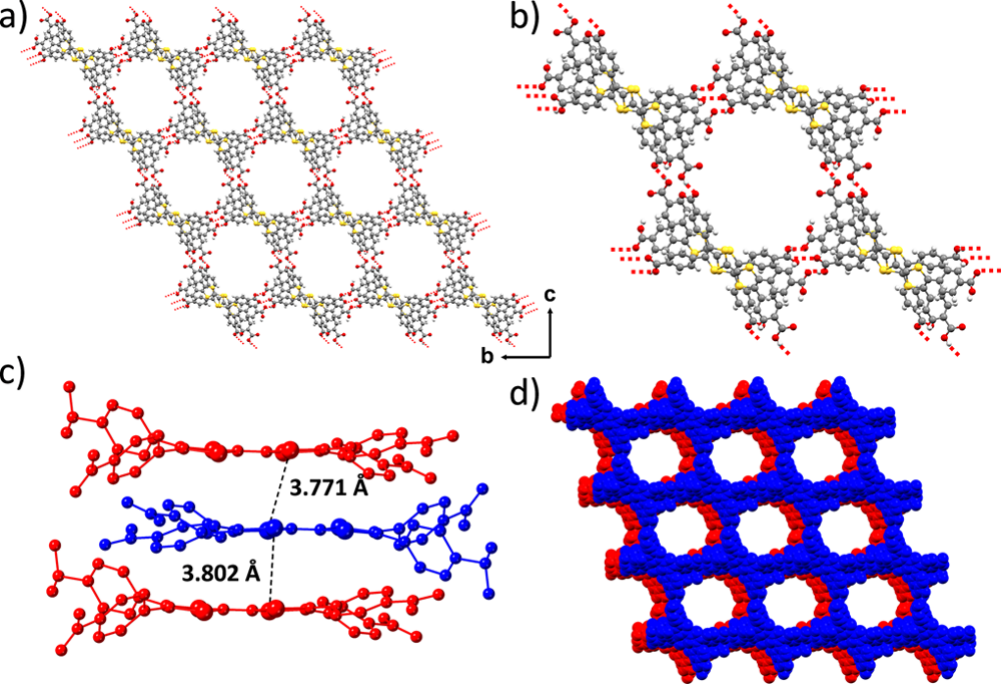
(a) Crystal structure of **MUV-21** showing the mesoporous channels along the *a*-axis. (b) Closer view of one microporous channel along the *a* axis. (c) Distances between sulfur atoms of TTF units in adjacent layers. (d) Different layers showing the non-interpenetrated framework along the *a* axis. The DMF molecules and DMA^+^ cations present in the pores have been omitted for clarity.

TTF-tetrabenzoic ether was prepared from the coupling of TTF and ethyl 4-bromobenzoate, followed by hydrolysis and acidification (see Section S1 in the Supporting Information). Importantly, acidification resulted in the protonation of three among the four carboxylate groups, resulting in an amorphous material of formula NaH_3_TTFTB, as seen with ^1^H NMR (see Figure S1) and energy-dispersive X-ray analysis (EDAX) (see Figure S2 and Table S1). Dissolution of NaH_3_TTFTB in THF and heating at 80 °C forms a transparent dark-red solution, and addition of diethyl ether causes the appearance of red needle-like crystals of **MUV-20a** after few minutes at room temperature (Figure 1). Upon washing the crystals with diethyl ether, the THF molecules are replaced by diethyl ether molecules, yielding crystals of **MUV-20b** (Figure 2). These crystals were suitable for single-crystal X-ray diffraction analysis (see Table S2 for crystallographic details). Quite differently, crystallization from DMF at 105 °C resulted in large single crystals of **MUV-21** (Figure 3 and Table S2). Phase purity of each sample was confirmed by powder X-ray diffraction (Figures S28–S30).

**MUV-20a** forms a 2D hydrogen-bonded network involving three carboxylic acids from each molecule (Figure 1a), with O···O hydrogen-bond distances in the range of 2.60–2.73 Å (Figure S3), forming voids with dimensions of ca. 7 × 9 Å (Figure S4). The 2D layers are interconnected via π–π stacking between the benzene moieties possessing the carboxylate group that is not involved in hydrogen bonding (Figure 1b), with a distance of 3.28 Å between parallel benzene groups (Figure S5). As a result, interpenetration between adjacent layers occurs (blue and red in Figure 1d). The stacking of the layers results in a 3D-networked, rhombic, interpenetrated framework. The structural features of **MUV-20a** are completed with π–π stacking between the TTF moieties (Figures 1c, S6, and S7), with shortest S···S distances of 3.66 and 4.45 Å for closest and furthest neighbors, respectively (Tables S3 and S4).

Similarly, **MUV-20b** forms a 2D hydrogen-bonded network involving three carboxylic acids from each molecule (Figure 2a), with O···O hydrogen-bond distances in the range 2.63–2.67 Å (Figures 2b and S12), forming narrow voids with dimensions of ca. 9 × 9 Å (Figure S13). The stacking of the layers results in a 3D-networked, rhombic, non-interpenetrated framework (blue and red in Figure 2d). The structural features of **MUV-20b** are completed with π–π stacking between the TTF moieties (Figures 2c, S14, and S15), with shortest S···S distances of 3.64 and 4.45 Å for closest and furthest neighbors, respectively (Tables S5 and S6).

In contrast, structural analysis of **MUV-21** (Figure 3) reveals that the three carboxylic acids and the carboxylate group are involved in hydrogen-bonding interactions, with O···O hydrogen bond distances in the range 2.46–2.58 Å (Figure S20). The crystalline structure is connected in a 2D framework with no interpenetration between adjacent layers (Figure 3d), forming large channels along the *a* axis (Figure 3a) of ca. 20 × 15 Å (Figures 3b and S21). The TTF moieties are closer on average than in **MUV-20a** and in **MUV-20b**, with shortest S···S distances of 3.77 and 3.80 Å (Figures 3c, S22, and S23, and Tables S7 and S8). In contrast to **MUV-20a** and **MUV-20b**, the structure of **MUV-21** contains dimethylammonium cations (DMA^+^) originating from DMF decomposition.

**Figure 4:**
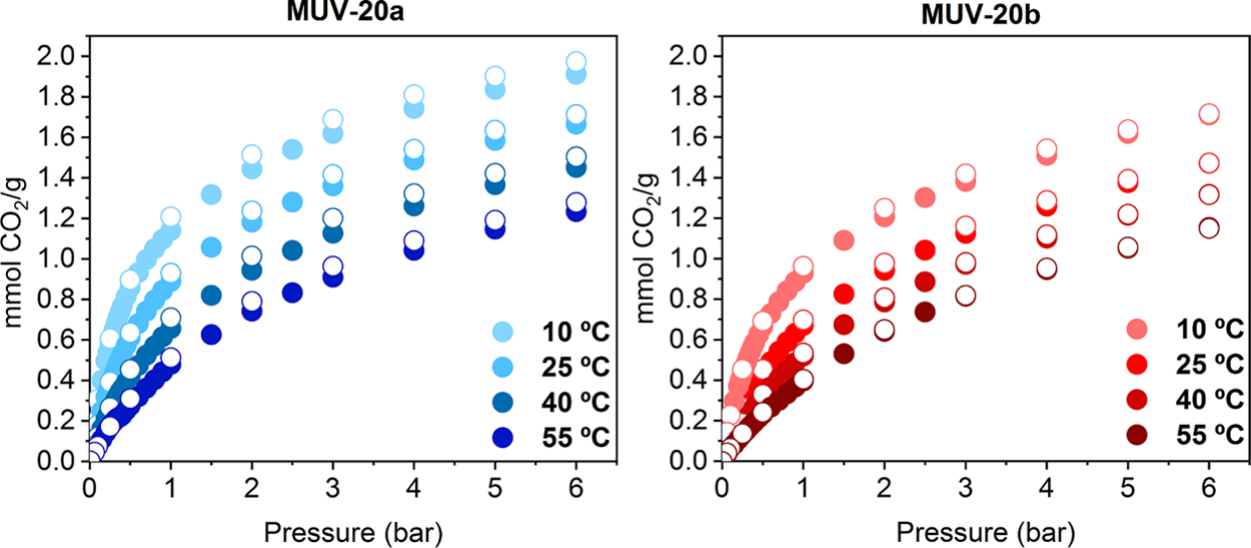
Gas adsorption isotherms of CO_2_ on **MUV-20a** (blue) and **MUV-20b** (red) at different temperatures. CO_2_ adsorption and desorption capacities are shown in closed and open circles, respectively.

The calculated free space of **MUV-20a**, **MUV-20b**, and **MUV-21** is 22% (308 Å^3^ as void volume), 15% (250 Å^3^), and 27.6% (788.3 Å^3^), respectively (see Figures S9, S17, and S24). However, despite the apparent large porosity of **MUV-21**, this material collapses upon activation (Figure S38), probably caused by the stronger interactions of DMF molecules with the framework, as previously observed for analogous systems.^40^ The crystallinity of **MUV-20a** and **MUV-20b** is maintained upon activation (1 h at 70 °C), remaining stable up to 330 °C (Figures S35–S37). CO_2_ adsorption isotherms reveal a maximum CO_2_ adsorption capacity, at 10 °C and 6 bar, of 1.91 and 1.71 mmol g^–1^ for **MUV-20a** and **MUV-20b**, respectively (see Figure 4 and Table S9). These values are comparable to other HOFs found in the literature, such as **HOF-6**(43) and **HOF-7**,^44^ although it is still far from the CO_2_ record uptake of 4.02 mmol g^–1^ of **HOF-5** at 1 bar and 296 K.^22^

**Figure 5:**
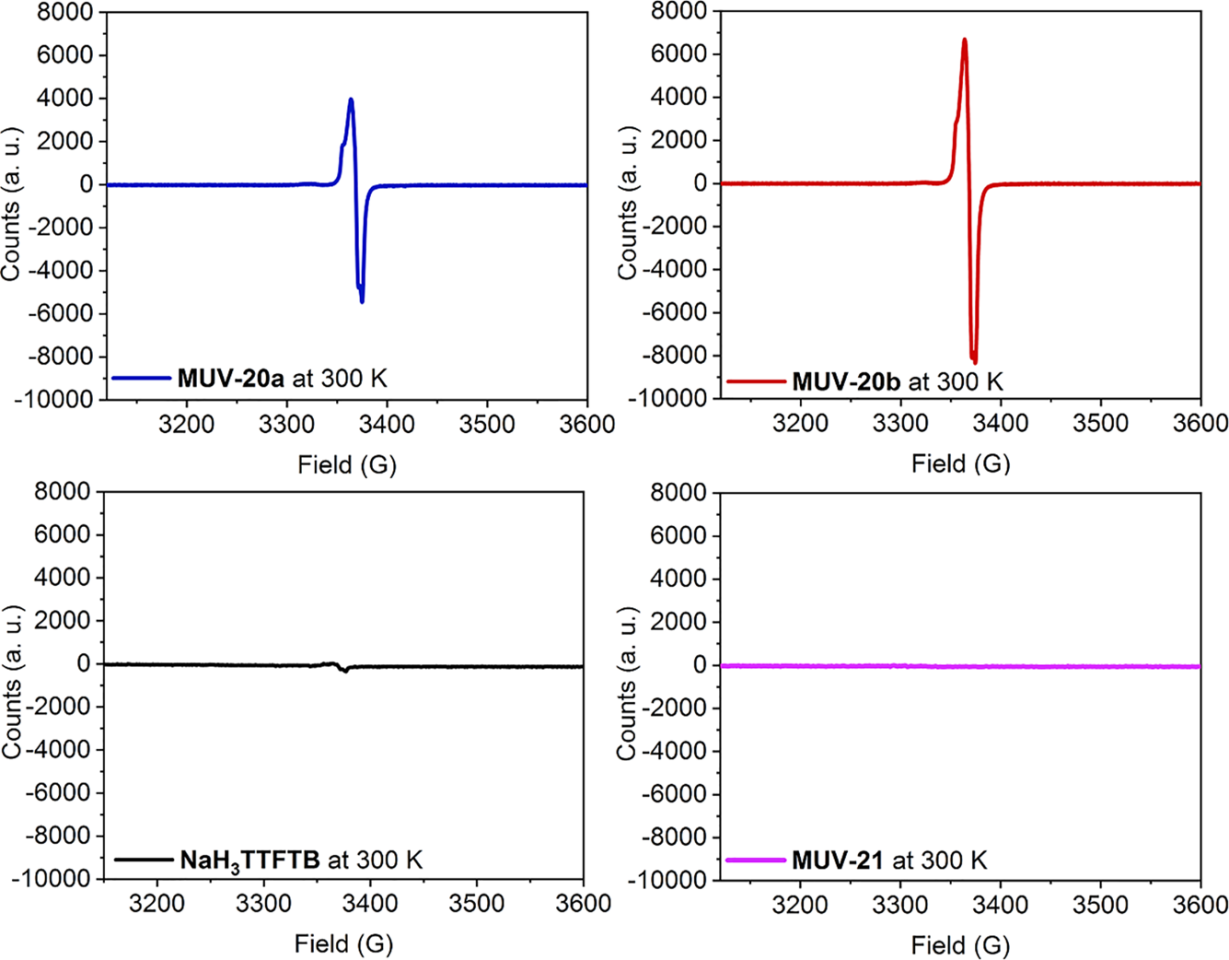
EPR spectra of **MUV-20a** (blue), **MUV-20b** (red), **MUV-21** (purple), and the NaH_3_TTFTB (black) ligand at room temperature.

Comparing the two families of HOFs, **MUV-21** has a higher void space and slightly shorter mean distances between TTF units than both **MUV-20a** and **MUV-20b**, which seems to indicate a better material for combining porosity and electrical conductivity, although, as already shown, the stability of **MUV-21** upon activation is limited. However, close inspection of the structures reveals more relevant differences between the two families. Thus, whereas a DMA^+^ cation is present in **MUV-21** to counterbalance the negative charge of the deprotonated carboxylate, no additional cation is present in **MUV-20a** or **MUV-20b**. EPR measurements actually evidence the presence of an unpaired electron in both **MUV-20a** and **MUV-20b**, which is neither present in the as-synthesized NaH_3_TTFTB nor in **MUV-21** (Figure 5). The *g* values (*g* = 2.07) are in good agreement with an oxidized tetrathiafulvalene moiety. Thus, we hypothesize that compounds **MUV-20a** and **MUV-20b** can be considered as zwitterionic HOFs formed by molecules with a negative charge at one carboxylate unit and a positive charge at the TTF core. Unlike DMF, both THF and diethyl ether solvent molecules have a tendency to form peroxides,^45−47^ which we believe is at the origin of TTF spontaneous oxidation.

**Figure 6:**
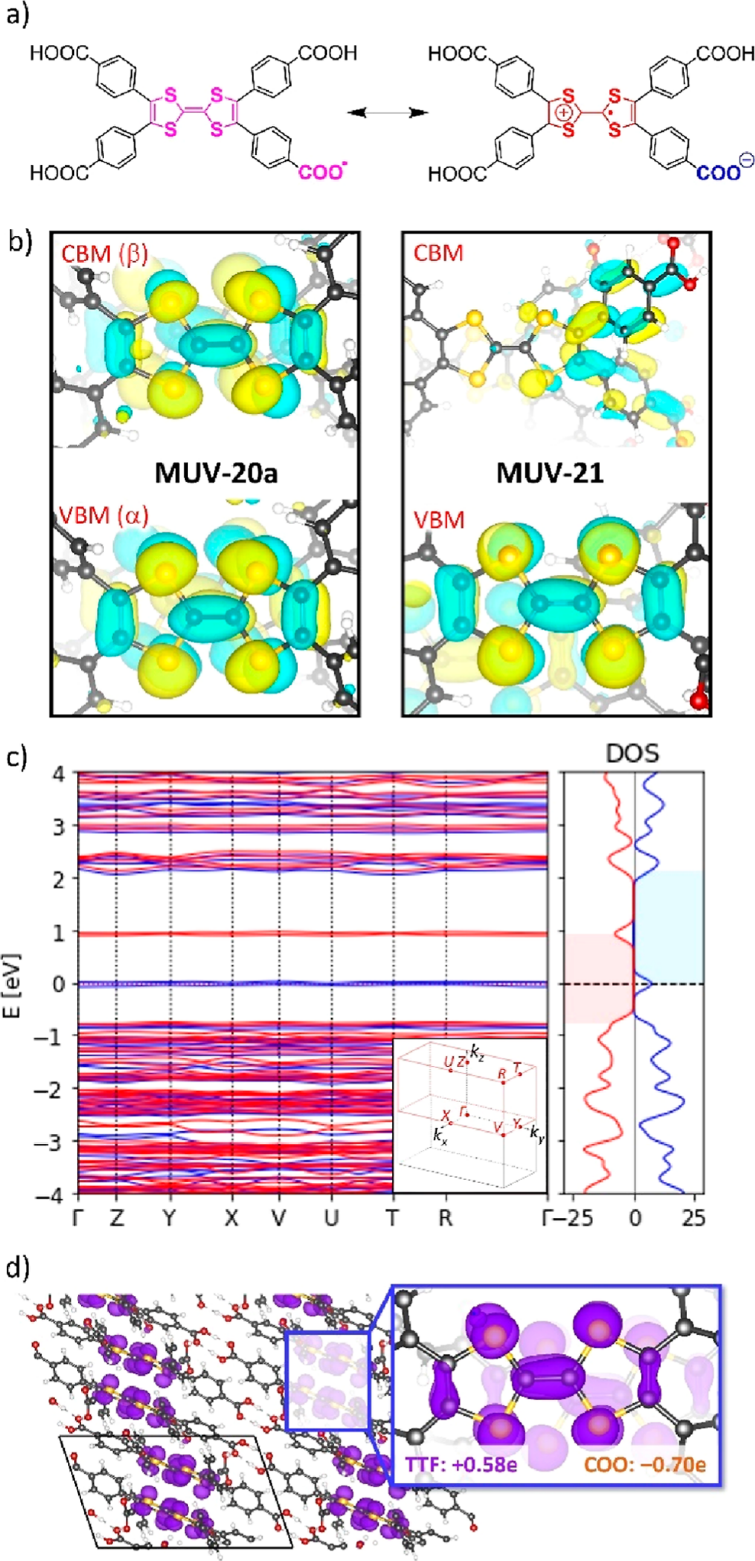
(a) Schematic representation of deprotonated TTFTB as a carboxylic radical (left) and a zwitterion (right) in **MUV-20a** and **MUV-20b**. (b) VBM and CBM calculated for **MUV-20a** and **MUV-21** with an isovalue contour of 0.05 atomic units (au). (c) Electronic band structure diagram (left) and density of states (right) calculated for the zwitterionic ferromagnetic **MUV-20a** at the HSE06 level. The Fermi level is set to the VBM. Spin-up α and spin-down β channels are displayed in blue and red, respectively. The band gaps of α (2.15 eV) and β (1.68 eV) channels are colored in blue and red, respectively. (d) Spin density of **MUV-20a** represented with an isovalue contour of 0.008 au. The charge accumulation increase (Δ*q*) for the TTF core and the carboxylate group with respect to the reference isolated, fully protonated TTFTB ligand is indicated.

In order to confirm the zwitterionic-radical nature of **MUV-20a** and **MUV-20b**, compared to **MUV-21**, theoretical calculations were performed under the density functional theory (DFT) framework using periodic boundary conditions. The minimum-energy crystal structure of the three compounds was obtained upon full lattice and ionic relaxation at the PBEsol level starting from the experimental X-ray data (see Section S9 in the Supporting Information for computational details). The optimized lattice parameters of the HOFs compare well with the values inferred for the synthetized crystals (see Table S13). Importantly, the presence of DMA^+^ cations in **MUV-21** leads to a H-bonding connection between the countercation and two TTFTB units (one of them with a deprotonated carboxylic acid; COO^–^), involving two diagonal carboxylic positions of the ligands, whereas a H-bond network connecting four TTFTBs is found for the other two diagonal positions (Figure S48). In contrast, the frameworks of **MUV-20a** and **MUV-20b** can grow without any need for countercations toward the formation of a HOF with singly deprotonated TTFTB ligands and overall charge neutrality. The most stable site for the deprotonated carboxylic acid of TTFTB was assessed by placing it at the four inequivalent acid groups of the unit cell of **MUV-20a** and **MUV-20b** and fully relaxing the resulting structure (Figure S49). Theoretical calculations predict the preferentially deprotonated carboxylic group as that showing short O(COO)···S(TTF) contacts in the range 2.7–3.1 Å for **MUV-20a** and **MUV-20b** (Figure S49). As experimental pieces of evidence indicate that the TTFTB ligand is singly deprotonated upon formation of the porous frameworks, the two ligands of the unit cell were considered to be singly deprotonated in their most stable isomer.^48^ Moreover, as no countercation is present in the neutral crystal structure of **MUV-20a** and **MUV20b**, the negative charge of the carboxylate group has to be compensated by the formation of TTF^•+^ through spontaneous oxidation of the electron-donor moiety. We then propose two possible scenarios: (i) the extra electron of the carboxylate moves to the cationic TTF, leading to a carboxylic radical TTFTB species, or (ii) the ligand remains in its zwitterionic form engendering persistent charge carriers and a radical in the TTF core (Figure 6a). Preliminary calculations at the molecular level indicate that although both situations are computed very similar in energy, the zwitterionic species is more stable than the radical COO^•^ analogue in singly deprotonated, neutral TTFTB when including boundary conditions (Figures S50 and S51).

**MUV-20a** and **MUV-20b** can be modeled in a ferromagnetic or an antiferromagnetic configuration by considering the interaction between neighboring radical TTFTB ligands (Figure S52). Theoretical calculations predict that the ferromagnetic alignment is 0.33 and 0.17 eV more stable in **MUV-20a** and **MUV-20b**, respectively, than the antiferromagnetic phase at the HSE06 level. In fact, the antiferromagnetic solution leads to the coupling of the unpaired electrons of the interacting TTFTB radicals, prompting a “diamagnetic”-like configuration (see Figures S53 and S54). As experimental data confirm the presence of the TTF radical, and following the theoretical relative stability calculations indicating that the ferromagnetic configuration is more stable, from now on, we focus on the ferromagnetic phase of both **MUV-20a** and **MUV-20b**.

Analysis of the electronic band structure and density of states (DOS) reveals a band gap of 1.42 eV in HOF **MUV-21** (Figure S55), with a valence-band maximum (VBM) centered on the TTF unit and a conduction-band minimum (CBM) localized over the benzoic acid groups (Figure 6b). On the other hand, the band gap in **MUV-20a** and **MUV-20b** is predicted to be 1.68 and 1.52 eV, respectively, in the β-spin manifold (Figures 6c and S56, respectively). In this case, both the VBM(α) and CBM(β) are centered over the TTF moiety (Figures 6b, S57, and S58), suggesting the formation of the radical TTF^•+^. The spin density calculated for **MUV-20a** (Figure 6d) and **MUV-20b** (Figure S59) is fully located over the TTF core, which experiences a charge accumulation increase (Δ*q*) of +0.58 and +0.55e, respectively, with respect to the isolated, fully protonated TTFTB ligand, whereas the deprotonated carboxylic group is predicted with a Δ*q* of −0.70 and −0.66e, respectively. These results evidence the presence of an oxidized TTF core (with an unpaired electron; TTF^•+^) and a negatively charged carboxylate group (COO^–^) per TTFTB ligand, therefore confirming the zwitterionic nature of **MUV-20a** and **MUV-20b** (Figure 6a).

In our HOFs, the TTF units are assembled in a π-stacking arrangement along the *a*-axis; thus, charge conduction after carrier generation may arise anisotropically in one dimension. The electronic band structure calculated for our series of HOFs displays flat bands along the full *k*-path of the first Brillouin zone (Figures 6c, S53, and S56 for **MUV-20a**, **MUV-20b**, and **MUV-21**, respectively), which points toward a hopping regime as the dominant charge-transport mechanism. The size of the polaron was estimated from the spin density contours to localize over few (1–3) TTF units in the three HOFs (Figure S60),^49^ supporting a hopping-like transport.

**Figure 7:**
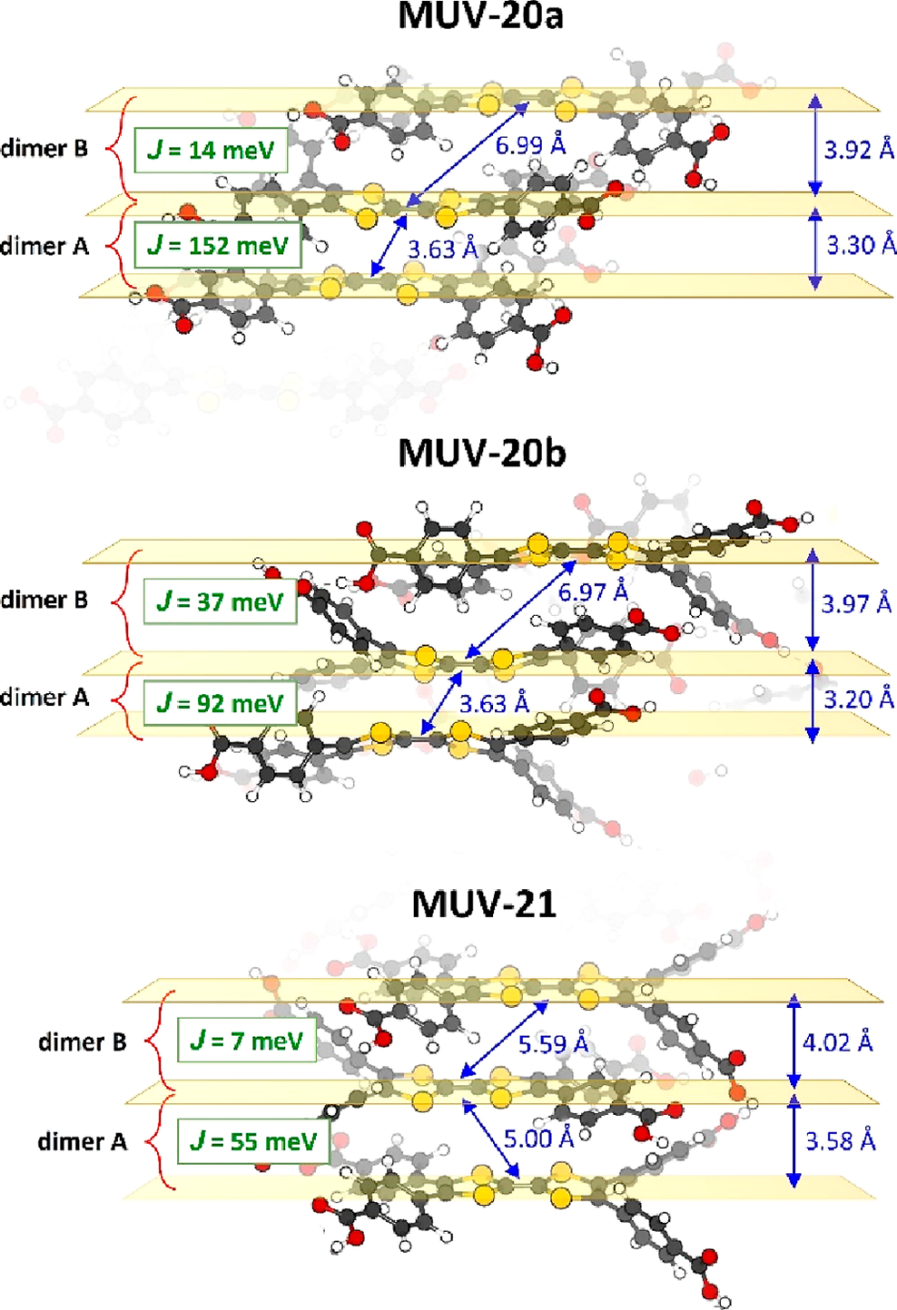
TTFTB···TTFTB dimeric pairs in the π-stacking arrangement extracted from the minimum-energy crystal structure of **MUV-20a** (top), **MUV-20b** (middle), and **MUV-21** (bottom). Relevant intermolecular distances and electronic couplings (*J*) between pairs are indicated.

To shed light on the conducting properties of **MUV-20a**, **MUV-20b**, and **MUV-21**, the electronic couplings (*J*) between neighboring TTFTB ligands, the reorganization energy, and the resulting charge-transfer rate constants (*k*) were estimated by means of the Marcus theory and molecular calculations (see Section S9 in the Supporting Information for details). The energy splitting between the highest-occupied crystal orbitals involving the two TTFs of the unit cell (VBM energy splitting)^50^ was evaluated as a bare approximation of the electronic communication between TTF pairs, showing a larger mean value for zwitterionic **MUV-20a** (175 meV) and **MUV-20b** (158 meV) than for **MUV-21** (90 meV). Accurate electronic couplings for the closest interacting TTF pairs (dimer A in Figure 7) confirm this picture, with *J* values of 152, 92, and 55 meV for **MUV-20a**, **MUV-20b**, and **MUV-21**, respectively, which are in the same order of magnitude of those reported for prototypical organic semiconductors.^51,52^ The coupling in the weakly interacting TTF pair (dimer B) is predicted one order of magnitude smaller (14, 37, and 7 meV for **MUV-20a**, **MUV-20b**, and **MUV-21**, respectively), which stems from the less efficient π-stacking of the TTF moieties (core-to-core intermolecular distances > 5 Å; Figure 7). Overall, considering the smaller electronic coupling as the limiting step for charge conduction, we predict rate constants of 8 × 10^10^, 6 × 10^11^, and 2 × 10^10^ s^–1^ for **MUV-20a**, **MUV-20b**, and **MUV-21**, respectively (Table S14). As conductivity depends on charge mobility and carrier concentration, zwitterionic **MUV-20a** and **MUV-20b**, which present larger TTFTB electronic couplings and already contain the TTF^•+^/COO^–^ polaron in the framework, are expected to show a significant enhancement of charge transport compared to bare **MUV-21**.

Transport measurements for **MUV-20a**, **MUV-20b**, and **MUV-21** were performed using two-contact probe pressed-pellet devices measured at room temperature (300 K) (Figure S46), revealing conductivity values of 6.07 × 10^–7^, 1.35 × 10^–6^, and 6.23 × 10^–9^ S cm^–1^, respectively (see Table S11). This indicates that **MUV-20a** and **MUV-20b** behave as semiconductors, presenting electrical conductivities similar to those reported for **Cd**_**2**_**(TTFTB)**(53) and **TTF-COF**(39) after being oxidized with iodine and more than 10 times higher than iodine-doped **HOF-110**(42) (see Table S12 for comparative values with all reported MOFs and HOFs containing the TTFTB unit). In contrast, non-zwitterionic **MUV-21** behaves as an insulator due to the absence of charge carriers. Thus, conductivity measurements confirm the semiconducting nature of zwitterionic **MUV-20a** and **MUV-20b**, which present record charge transport in HOFs without the need for further oxidation due to the existence of persistent positive charge carriers in the form of TTF^•+^ radicals.

**Figure 8:**
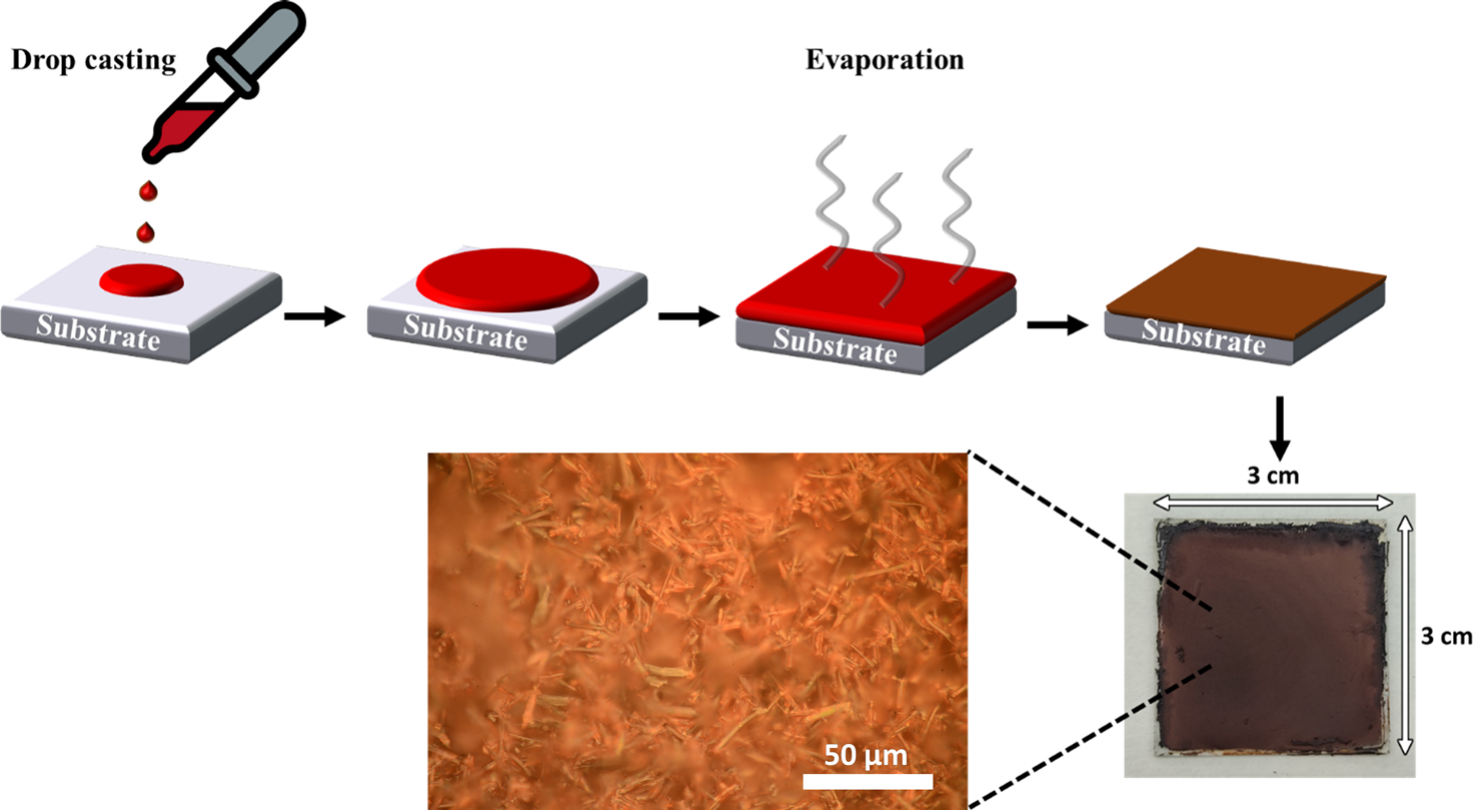
Preparation of a thin film of **MUV-20a** using drop casting.

Due to the fast synthesis of the crystals under ambient conditions, the processability to prepare thin films of these porous semiconductor materials was examined for **MUV-20a**. Drop-casting of a 0.02 M THF solution of NaH_3_TTFTB on a 3 × 3 cm^2^ glass support yields a continuous thin film, as shown in Figure 8. This illustrates the facile processability of HOFs, contrary to what is commonly found for MOFs and COFs, which paves the way to their implementation in charge-storage devices, electrochemical sensors, or as electrocatalysts.

## Conclusions

A new strategy for the synthesis of conducting HOFs is presented, based on the *in situ* oxidation of a partially deprotonated TTF-based molecule, which yields a zwitterionic material. This has been exemplified with two different structures, namely, **MUV-20a** and **MUV-20b**, which are obtained using the TTFTB building block and have different solvents in the pores (THF and ether, respectively). On the contrary, the solvothermal approach in DMF commonly used for the preparation of porous materials yields a non-zwitterionic HOF, denoted **MUV-21**. Whereas **MUV-21** collapses during pore activation, **MUV-20a** and **MUV-20b** display high stability upon evacuation, likely due to their zwitterionic behavior and lower pore sizes, and offer large CO_2_ sorption capacities up to 1.91 and 1.71 mmol g^–1^, respectively. Theoretical calculations confirm the zwitterionic nature of these two HOFs and the emergence of efficient charge transport properties without the need of postsynthetic oxidation treatments. Transport measurements indicate conductivity values of 6.07 × 10^–7^ and 1.35 × 10^–6^ S·cm^–1^ for **MUV-20a** and **MUV-20b**, respectively, thus placing these materials as the highest conducting HOFs so far, with the added value of facile synthesis for further application in organic electronics.

## Experimental Section

**H**_**4**_**TTFTB** was prepared as previously described,^38,54^ and all details can be found in the Supporting Information.

### Crystallization of **MUV-20a**

2 mL of tetrahydrofuran (THF) was added to 20 mg of **NaH**_**3**_**TTFTB** in a 40 mL vial. The mixture was sonicated for 2 min until a transparent dark-red solution was formed. This solution was heated for 5 min at 80 °C by placing the vial on top of a heating plate. Then, 30 mL of diethyl ether was added, resulting in red needle-like single crystals of **MUV-20a**, after few minutes at room temperature, in which THF molecules are embedded in the pores (Figure S11). Finally, the crystals were filtered in air (12 mg, yield 60%).

### Crystallization of **MUV-20b**

Upon formation of red needle-like single crystals of **MUV-20a**, these were washed with diethyl ether, yielding red needle-like single crystals of **MUV-20b** (16 mg, yield 80%), in which THF molecules are replaced by diethyl ether molecules (Figure S19). In fact, immersion of crystals of **MUV-20a** in diethyl ether causes the transformation of **MUV-20a** to **MUV-20b** (see Figure S31 in the Supporting Information).

### Crystallization of **MUV-21**

20 mg of **NaH**_**3**_**TTFTB** was solved with 1 mL of DMF in a 4 mL vial at 105 °C for 60 h in an oven with heating and cooling ramps of 0.5 °C min^–1^, resulting in red needle-like single crystals of **MUV-21** that were washed with diethyl ether at room temperature (4 mg, yield: 20%).

### Single-Crystal Diffraction

X-ray data for compound **MUV-20a** were collected at beamline I19 in a Diamond Light Source synchrotron at a temperature of 100 K using a Pilatus 6M detector.^55^ X-ray data for the compound **MUV-20b** were collected at 100 K using a Rigaku FR-X rotating anode with a Hypix 6000HE detector. X-ray data for compound **MUV-21** were collected at 120 K using a Rigaku microfocus Supernova with an Atlas CCD detector. Data were measured using GDA and CrysAlisPro suite of programs. X-ray data were processed and reduced using the CrysAlisPro suite of programs. Absorption correction was performed using empirical methods (SCALE3 ABSPACK) based upon symmetry-equivalent reflections combined with measurements at different azimuthal angles. The crystal structures were solved and refined against all *F*^2^ values using the SHELX and Olex 2 suite of programs.^56,57^ All atoms were refined anisotropically with the exception of the partially occupied diethyl ether molecule in **MUV-20b** and a solvent DMF molecule in **MUV-21**. Hydrogen atoms were placed in the calculated positions. Details of each refinement can be found in the Supporting Information. CCDC 2153374–2153376 contains the supplementary crystallographic data for this paper. These data can be obtained free of charge via www.ccdc.cam.ac.uk/conts/retrieving.html (or from the Cambridge Crystallographic Data Centre, 12 Union Road, Cambridge CB21EZ, UK; fax: (+44)1223-336-033; or deposit@ccdc.cam.ac.uk).

### Gas Sorption

**MUV-20a** and **MUV-20b** crystals were activated at 70 °C during 1 h. High-pressure adsorption isotherms of CO_2_ were measured at different temperatures from 10 to 55 °C in an IGA-100 gravimetric analyzer (Hiden Isochem) using approximately 35 mg of the different samples. The heat of adsorption was calculated according to the Clausius–Clapeyron equation from the isotherms measured at different temperatures (see Supporting Information Section S6).

### Electron Paramagnetic Resonance

Electron paramagnetic resonance was measured using a Bruker ELEXYS E580 at 300 K.

### Theoretical Calculations

Quantum chemical calculations were carried out within the DFT framework as implemented in the all-electron full-potential FHI-AIMS electronic structure package. Minimum-energy geometries for **MUV-20a**, **MUV-20b**, and **MUV-21** were obtained starting from the experimental crystal structures, and after full lattice and ionic relaxation using the GGA-type PBEsol functional and the numeric atom-centered orbital tier-1 basis set. Dispersion forces were treated by means of the van der Waals Hirshfeld correction. The electronic band structure and density of states were obtained by means of the hybrid HSE06 functional. A full *k*-path in the *P*1̅ first Brillouin zone and a 3 × 3 × 3 k-grid were employed. The electronic couplings of the different TTF dimers were computed under the fragment-orbital DFT framework, as implemented in FHI-AIMS. See the Supporting Information for full computational details.

### Conductivity Measurements

Powders of **MUV-20a**, **MUV-20b**, and **MUV-21** were pressed to form pellets (*P* ≈ 5 US tons), cut in rectangular shapes, and allowed to make contact with platinum wires (Goodfellow, 99.99%, 25 μm of diameter) and silver conductive paint (RS 123-9911) in a two-probe configuration (Figure S46). Two different pellets were measured for each sample. The geometrical factors (width, length, and thickness) were measured using an optical microscope. **MUV-21** was also measured using a single crystal (Figure S46).

### Film Recrystallization

For the synthesis of the film, 40 mg of NaH_3_TTFTB was dissolved in 2 mL of THF and then was drop-cast on a 3 × 3 cm^2^ glass. After a few minutes, the solution was dried and a continuous film forming dark small crystals appeared on the surface of the support.

## Supporting Information Available

General methods, materials, crystallographic data, computational details, and supporting theoretical data (PDF)

## Accession Codes

CCDC 2153374–2153376 contain the supplementary crystallographic data for this paper. These data can be obtained free of charge via www.ccdc.cam.ac.uk/data_request/cif, or by emailing data_request@ccdc.cam.ac.uk, or by contacting The Cambridge Crystallographic Data Centre, 12 Union Road, Cambridge CB2 1EZ, UK; fax: +44 1223 336033.

## References

[ref1] Hendon C. H.; Tiana D.; Walsh A. Conductive Metal-Organic Frameworks and Networks: Fact or Fantasy?. Phys. Chem. Chem. Phys. 2012, 14, 13120–13132. 10.1039/c2cp41099k. 22858739

[ref2] Souto M.; Perepichka D. F. Electrically Conductive Covalent Organic Frameworks: Bridging the Fields of Organic Metals and 2D Materials. J. Mater. Chem. C 2021, 9, 10668–10676. 10.1039/d1tc00750e.

[ref3] Zhou H.-C. J.; Kitagawa S. Metal-Organic Frameworks (MOFs). Chem. Soc. Rev. 2014, 43, 5415–5418. 10.1039/c4cs90059f. 25011480

[ref4] Côté A. P.; Benin A. I.; Ockwig N. W.; O’Keeffe M.; Matzger A. J.; Yaghi O. M. Porous, Crystalline, Covalent Organic Frameworks. Science 2005, 310, 1166–1171. 10.1126/science.1120411. 16293756

[ref5] Calbo J.; Golomb M. J.; Walsh A. Redox-Active Metal-Organic Frameworks for Energy Conversion and Storage. J. Mater. Chem. A 2019, 7, 16571–16597. 10.1039/c9ta04680a.

[ref6] Xie L. S.; Skorupskii G.; Dincă M. Electrically Conductive Metal-Organic Frameworks. Chem. Rev. 2020, 120, 8536–8580. 10.1021/acs.chemrev.9b00766. 32275412 PMC7453401

[ref7] Meng Z.; Stolz R. M.; Mirica K. A. Two-Dimensional Chemiresistive Covalent Organic Framework with High Intrinsic Conductivity. J. Am. Chem. Soc. 2019, 141, 11929–11937. 10.1021/jacs.9b03441. 31241936

[ref8] Hmadeh M.; Lu Z.; Liu Z.; Gándara F.; Furukawa H.; Wan S.; Augustyn V.; Chang R.; Liao L.; Zhou F.; Perre E.; Ozolins V.; Suenaga K.; Duan X.; Dunn B.; Yamamto Y.; Terasaki O.; Yaghi O. M. New Porous Crystals of Extended Metal-Catecholates. Chem. Mater. 2012, 24, 3511–3513. 10.1021/cm301194a.

[ref9] Dong R.; Zhang T.; Feng X. Interface-Assisted Synthesis of 2D Materials: Trend and Challenges. Chem. Rev. 2018, 118, 6189–6235. 10.1021/acs.chemrev.8b00056. 29912554

[ref10] Hoffmann R. Interaction of Orbitals through Space and through Bonds. Acc. Chem. Res. 1971, 4, 1–9. 10.1021/ar50037a001.

[ref11] Batra A.; Kladnik G.; Vázquez H.; Meisner J. S.; Floreano L.; Nuckolls C.; Cvetko D.; Morgante A.; Venkataraman L. Quantifying Through-Space Charge Transfer Dynamics in π-Coupled Molecular Systems. Nat. Commun. 2012, 3, 1086 10.1038/ncomms2083. 23011140

[ref12] Sirringhaus H.; Brown P. J.; Friend R. H.; Nielsen M. M.; Bechgaard K.; Langeveld-Voss B. M. W.; Spiering A. J. H.; Janssen R. A. J.; Meijer E. W.; Herwig P.; de Leeuw D. M. Two-Dimensional Charge Transport in Self-Organized, High-Mobility Conjugated Polymers. Nature 1999, 401, 685–688. 10.1038/44359.

[ref13] Talin A. A.; Centrone A.; Ford A. C.; Foster M. E.; Stavila V.; Haney P.; Kinney R. A.; Szalai V.; El Gabaly F.; Yoon H. P.; Léonard F.; Allendorf M. D. Tunable Electrical Conductivity in Metal-Organic Framework Thin-Film Devices. Science 2014, 343, 66–69. 10.1126/science.1246738. 24310609

[ref14] Johnson E. M.; Ilic S.; Morris A. J. Design Strategies for Enhanced Conductivity in Metal-Organic Frameworks. ACS Cent. Sci. 2021, 7, 445–453. 10.1021/acscentsci.1c00047. 33791427 PMC8006162

[ref15] Rubio-Giménez V.; Galbiati M.; Castells-Gil J.; Almora-Barrios N.; Navarro-Sánchez J.; Escorcia-Ariza G.; Mattera M.; Arnold T.; Rawle J.; Tatay S.; Coronado E.; Martí-Gastaldo C. Bottom-Up Fabrication of Semiconductive Metal–Organic Framework Ultrathin Films. Adv. Mater. 2018, 30, 1704291 10.1002/adma.201704291. 29341257

[ref16] Ma K.; Li P.; Xin J. H.; Chen Y.; Chen Z.; Goswami S.; Liu X.; Kato S.; Chen H.; Zhang X.; Bai J.; Wasson M. C.; Maldonado R. R.; Snurr R. Q.; Farha O. K. Ultrastable Mesoporous Hydrogen-Bonded Organic Framework-Based Fiber Composites toward Mustard Gas Detoxification. Cell Rep. Phys. Sci. 2020, 1, 100024 10.1016/j.xcrp.2020.100024.

[ref17] Ko M.; Mendecki L.; Eagleton A. M.; Durbin C. G.; Stolz R. M.; Meng Z.; Mirica K. A. Employing Conductive Metal-Organic Frameworks for Voltammetric Detection of Neurochemicals. J. Am. Chem. Soc. 2020, 142, 11717–11733. 10.1021/jacs.9b13402. 32155057

[ref18] Meng Z.; Stolz R. M.; Mendecki L.; Mirica K. A. Electrically-Transduced Chemical Sensors Based on Two-Dimensional Nanomaterials. Chem. Rev. 2019, 119, 478–598. 10.1021/acs.chemrev.8b00311. 30604969

[ref19] Suzuki Y.; Gutiérrez M.; Tanaka S.; Gomez E.; Tohnai N.; Yasuda N.; Matubayasi N.; Douhal A.; Hisaki I. Construction of Isostructural Hydrogen-Bonded Organic Frameworks: Limitations and Possibilities of Pore Expansion. Chem. Sci. 2021, 12, 9607–9618. 10.1039/d1sc02690a. 34349933 PMC8293819

[ref20] Wang B.; Lin R.-B.; Zhang Z.; Xiang S.; Chen B. Hydrogen-Bonded Organic Frameworks as a Tunable Platform for Functional Materials. J. Am. Chem. Soc. 2020, 142, 14399–14416. 10.1021/jacs.0c06473. 32786796

[ref21] Lin R.-B.; He Y.; Li P.; Wang H.; Zhou W.; Chen B. Multifunctional Porous Hydrogen-Bonded Organic Framework Materials. Chem. Soc. Rev. 2019, 48, 1362–1389. 10.1039/c8cs00155c. 30676603 PMC11061856

[ref22] Wang H.; Li B.; Wu H.; Hu T.-L.; Yao Z.; Zhou W.; Xiang S.; Chen B. A Flexible Microporous Hydrogen-Bonded Organic Framework for Gas Sorption and Separation. J. Am. Chem. Soc. 2015, 137, 9963–9970. 10.1021/jacs.5b05644. 26214340

[ref23] Li P.; He Y.; Arman H. D.; Krishna R.; Wang H.; Weng L.; Chen B. A Microporous Six-Fold Interpenetrated Hydrogen-Bonded Organic Framework for Highly Selective Separation of C_2_H_4_/C_2_H_6_. Chem. Commun. 2014, 50, 13081–13084. 10.1039/c4cc05506c. 25223376

[ref24] Liang W.; Carraro F.; Solomon M. B.; Bell S. G.; Amenitsch H.; Sumby C. J.; White N. G.; Falcaro P.; Doonan C. J. Enzyme Encapsulation in a Porous Hydrogen-Bonded Organic Framework. J. Am. Chem. Soc. 2019, 141, 14298–14305. 10.1021/jacs.9b06589. 31426638

[ref25] Sun Z.-B.; Li Y.-L.; Zhang Z.-H.; Li Z.-F.; Xiao B.; Li G. A Path to Improve Proton Conductivity: From a 3D Hydrogen-Bonded Organic Framework to a 3D Copper-Organic Framework. New J. Chem. 2019, 43, 10637–10644. 10.1039/c9nj02025j.

[ref26] Zhou H.; Ye Q.; Wu X.; Song J.; Cho C. M.; Zong Y.; Tang B. Z.; Hor T. S. A.; Yeow E. K. L.; Xu J. A Thermally Stable and Reversible Microporous Hydrogen-Bonded Organic Framework: Aggregation Induced Emission and Metal Ion-Sensing Properties. J. Mater. Chem. C 2015, 3, 11874–11880. 10.1039/c5tc02790j.

[ref27] Han Y.-F.; Yuan Y.-X.; Wang H.-B. Porous Hydrogen-Bonded Organic Frameworks. Molecules 2017, 22, 266 10.3390/molecules22020266. PMC6155736 28208825

[ref28] Jana A.; Bähring S.; Ishida M.; Goeb S.; Canevet D.; Sallé M.; Jeppesen J. O.; Sessler J. L. Functionalised Tetrathiafulvalene- (TTF-) Macrocycles: Recent Trends in Applied Supramolecular Chemistry. Chem. Soc. Rev. 2018, 47, 5614–5645. 10.1039/c8cs00035b. 30033473

[ref29] Otón F.; Pfattner R.; Oxtoby N. S.; Mas-Torrent M.; Wurst K.; Fontrodona X.; Olivier Y.; Cornil J.; Veciana J.; Rovira C. Benzodicarbomethoxytetrathiafulvalene Derivatives as Soluble Organic Semiconductors. J. Org. Chem. 2011, 76, 154–163. 10.1021/jo101817j. 21105721

[ref30] Ding B.; Solomon M. B.; Leong C. F.; D’Alessandro D. M. Redox-Active Ligands: Recent Advances towards Their Incorporation into Coordination Polymers and Metal-Organic Frameworks. Coord. Chem. Rev. 2021, 439, 213891 10.1016/j.ccr.2021.213891.

[ref31] Jin S.; Sakurai T.; Kowalczyk T.; Dalapati S.; Xu F.; Wei H.; Chen X.; Gao J.; Seki S.; Irle S.; Jiang D. Two-Dimensional Tetrathiafulvalene Covalent Organic Frameworks: Towards Latticed Conductive Organic Salts. Chem.—Eur. J. 2014, 20, 14608–14613. 10.1002/chem.201402844. 24782435

[ref32] Su J.; Yuan S.; Wang H.-Y.; Huang L.; Ge J.-Y.; Joseph E.; Qin J.; Cagin T.; Zuo J.-L.; Zhou H.-C. Redox-Switchable Breathing Behavior in Tetrathiafulvalene-Based Metal-Organic Frameworks. Nat. Commun. 2017, 8, 2008 10.1038/s41467-017-02256-y. 29222485 PMC5722820

[ref33] Castells-Gil J.; Mañas-Valero S.; Vitórica-Yrezábal I. J.; Ananias D.; Rocha J.; Santiago R.; Bromley S. T.; Baldoví J. J.; Coronado E.; Souto M.; Mínguez Espallargas G. Electronic, Structural and Functional Versatility in Tetrathiafulvalene-Lanthanide Metal–Organic Frameworks. Chem.—Eur. J. 2019, 25, 12636–12643. 31350922 10.1002/chem.201902855

[ref34] Souto M.; Romero J.; Calbo J.; Vitórica-Yrezábal I. J.; Zafra J. L.; Casado J.; Ortí E.; Walsh A.; Mínguez Espallargas G. Breathing-Dependent Redox Activity in a Tetrathiafulvalene-Based Metal-Organic Framework. J. Am. Chem. Soc. 2018, 140, 10562–10569. 10.1021/jacs.8b05890. 30040405 PMC6166999

[ref35] Souto M.; Calbo J.; Mañas-Valero S.; Walsh A.; Mínguez Espallargas G. Charge-Transfer Interactions between Fullerenes and a Mesoporous Tetrathiafulvalene-Based Metal-Organic Framework. Beilstein J. Nanotechnol. 2019, 10, 1883–1893. 10.3762/bjnano.10.183. 31598454 PMC6774073

[ref36] Souto M.; Santiago-Portillo A.; Palomino M.; Vitórica-Yrezábal I. J.; Vieira B. J. C.; Waerenborgh J. C.; Valencia S.; Navalón S.; Rey F.; García H.; Mínguez Espallargas G. A Highly Stable and Hierarchical Tetrathiafulvalene-Based Metal-Organic Framework with Improved Performance as a Solid Catalyst. Chem. Sci. 2018, 9, 2413–2418. 10.1039/c7sc04829g. 29732116 PMC5909329

[ref37] Vicent-Morales M.; Vitórica-Yrezábal I. J.; Souto M.; Mínguez Espallargas G. Influence of Interpenetration on the Flexibility of MUV-2. CrystEngComm 2019, 21, 3031–3035. 10.1039/c9ce00233b.

[ref38] Narayan T. C.; Miyakai T.; Seki S.; Dincă M. High Charge Mobility in a Tetrathiafulvalene-Based Microporous Metal-Organic Framework. J. Am. Chem. Soc. 2012, 134, 12932–12935. 10.1021/ja3059827. 22827709

[ref39] Ding H.; Li Y.; Hu H.; Sun Y.; Wang J.; Wang C.; Wang C.; Zhang G.; Wang B.; Xu W.; Zhang D. A Tetrathiafulvalene-Based Electroactive Covalent Organic Framework. Chem.—Eur. J. 2014, 20, 14614–14618. 10.1002/chem.201405330. 25266337

[ref40] Hisaki I.; Emilya Affendy N. Q.; Tohnai N. Precise Elucidations of Stacking Manners of Hydrogen-Bonded Two-Dimensional Organic Frameworks Composed of X-Shaped π-Conjugated Systems. CrystEngComm 2017, 19, 4892–4898. 10.1039/c7ce00183e.

[ref41] Gao X.-Y.; Li Y.-L.; Liu T.-F.; Huang X.-S.; Cao R. Single-Crystal-to-Single-Crystal Transformation of Tetrathiafulvalene-Based Hydrogen-Bonded Organic Frameworks. CrystEngComm 2021, 23, 4743–4747. 10.1039/d1ce00519g.

[ref42] Kirlikovali K. O.; Goswami S.; Mian M. R.; Krzyaniak M. D.; Wasielewski M. R.; Hupp J. T.; Li P.; Farha O. K. An Electrically Conductive Tetrathiafulvalene-Based Hydrogen-Bonded Organic Framework. ACS Mater. Lett. 2022, 4, 128–135. 10.1021/acsmaterialslett.1c00628.

[ref43] Yang W.; Yang F.; Hu T.-L.; King S. C.; Wang H.; Wu H.; Zhou W.; Li J.-R.; Arman H. D.; Chen B. Microporous Diaminotriazine-Decorated Porphyrin-Based Hydrogen-Bonded Organic Framework: Permanent Porosity and Proton Conduction. Cryst. Growth Des. 2016, 16, 5831–5835. 10.1021/acs.cgd.6b00924.

[ref44] Yang W.; Li B.; Wang H.; Alduhaish O.; Alfooty K.; Zayed M. A.; Li P.; Arman H. D.; Chen B. A Microporous Porphyrin-Based Hydrogen-Bonded Organic Framework for Gas Separation. Cryst. Growth Des. 2015, 15, 2000–2004. 10.1021/acs.cgd.5b00147.

[ref45] Jackson H. L.; McCormack W. B.; Rondestvedt C. S.; Smeltz K. C.; Viele I. E. Control of Peroxidizable Compounds. J. Chem. Educ. 1970, 47, A175 10.1021/ed047pa175.

[ref46] Kelly R. J. Review of Safety Guidelines for Peroxidizable Organic Chemicals. Chem. Health Saf. 1996, 3, 28–36. 10.1021/acs.chas.8b03515.

[ref47] Clark D. E. Peroxides and Peroxide - Forming Compounds, Chemical Health and Safety. Chem. Health Saf. 2001, 8, 12–22. 10.1016/s1074-9098(01)00247-7.

[ref48] Note that partial protonation (50% fully protonated TTFTBs in the unit cell) in **MUV-20a** and **MUV-20b** leads to a significant change in the electronic band structure of the HOF, with a predicted band gap in the β-channel of 0.51 and 0.52 eV, respectively (see Figures S56–S59).

[ref49] Note that there is no TTF^•+^ polaron in the bare **MUV-21**; hence, chemical or electrochemical oxidation processes need to be considered for hole-carrier generation in this material.

[ref50] The VBM splitting was estimated as the energy difference between the VBM and the VBM-1 divided by 2.

[ref51] Zeiser C.; Moretti L.; Geiger T.; Kalix L.; Valencia A. M.; Maiuri M.; Cocchi C.; Bettinger H. F.; Cerullo G.; Broch K. Permanent Dipole Moments Enhance Electronic Coupling and Singlet Fission in Pentacene. J. Phys. Chem. Lett. 2021, 12, 7453–7458. 10.1021/acs.jpclett.1c01805. 34339199

[ref52] Troisi A. Charge Transport in High Mobility Molecular Semiconductors: Classical Models and New Theories. Chem. Soc. Rev. 2011, 40, 2347–2358. 10.1039/c0cs00198h. 21409232

[ref53] Sun L.; Park S. S.; Sheberla D.; Dincă M. Measuring and Reporting Electrical Conductivity in Metal-Organic Frameworks: Cd2(TTFTB) as a Case Study. J. Am. Chem. Soc. 2016, 138, 14772–14782. 10.1021/jacs.6b09345. 27766856

[ref54] Mitamura Y.; Yorimitsu H.; Oshima K.; Osuka A. Straightforward Access to Aryl-Substituted Tetrathiafulvalenes by Palladium-Catalysed Direct C-H Arylation and Their Photophysical and Electrochemical Properties. Chem. Sci. 2011, 2, 2017–2021. 10.1039/c1sc00372k.

[ref55] Nowell H.; Barnett S. A.; Christensen K. E.; Teat S. J.; Allan D. R. I19, the Small-Molecule Single-Crystal Diffraction Beamline at Diamond Light Source. J. Synchrotron Radiat. 2012, 19, 435–441. 10.1107/s0909049512008801. 22514182

[ref56] Sheldrick G. M. Crystal Structure Refinement with SHELXL. Acta Crystallogr., Sect. C: Struct. Chem. 2015, 71, 3–8. 10.1107/s2053229614024218. 25567568 PMC4294323

[ref57] Dolomanov O. V.; Bourhis L. J.; Gildea R. J.; Howard J. A. K.; Puschmann H. OLEX2: A Complete Structure Solution, Refinement and Analysis Program. J. Appl. Crystallogr. 2009, 42, 339–341. 10.1107/s0021889808042726.

