# Supplementary material for: Semiconductor Porous Hydrogen-Bonded Organic Frameworks Based on Tetrathiafulvalene Derivatives
Source: J Am Chem Soc. 2022 May 16;144(20):9074–82. doi: 10.1021/jacs.2c01957 (PMC9136926; doi:10.1021/jacs.2c01957)
Supplement: Supplementary file 1 — ja2c01957_si_001.pdf [file ja2c01957_si_001.pdf]

Semiconductor Porous Hydrogen-bonded Organic Frameworks Based on Tetrathiafulvalene Derivatives

María Vicent-Morales, [†,⊥] María Esteve-Rochina, [†,⊥] Joaquín Calbo,[*,†] Enrique Ortí,[†] Iñigo J. Vitórica-Yrezábal,[‡] and Guillermo Mínguez Espallargas[*,†]

[†]Instituto de Ciencia Molecular (ICMol), Universidad de Valencia, c/ Catedrático José Beltrán, 2, 46980, Paterna, Spain

[‡]School of Chemistry, University of Manchester, Oxford Road, Manchester M13 9PL, United Kingdom

[⊥] These authors contributed equally to this work

Supporting Information

Table of contents

1. Synthesis 2

1.1. Synthesis of Et_4TTFTB 2

1.2. Synthesis of NaH_3TTFTB 2

1.3. Synthesis of MUV-20a. 4

1.4. Synthesis of MUV-20b. 4

1.5. Synthesis of MUV-21. 4

2. Crystal structures 5

2.1. Crystallographic information. 5

2.2. Crystal structure of MUV-20a 7

2.3. Crystal structure of MUV-20b 11

2.4. Crystal structure of MUV-21 15

3. Infrared spectroscopy (IR) 19

4. Powder X-ray diffraction (PXRD) 19

5. Thermogravimetric analysis 22

6. Gas adsorption 24

7. Scanning electron microscopy (SEM) 27

8. Electrical conductivity 28

9. Theoretical Calculations 32

10. References 44

1. Synthesis

All reagents and solvents employed for the synthesis were of high purity grade and were purchased from Sigma-Aldrich Co. and TCI. ^{1}H NMR spectra were recorded using a Bruker DPX300 (300 MHz) spectrometer and Me_4Si as an internal standard.

1.1. Synthesis of Et_4TTFTB

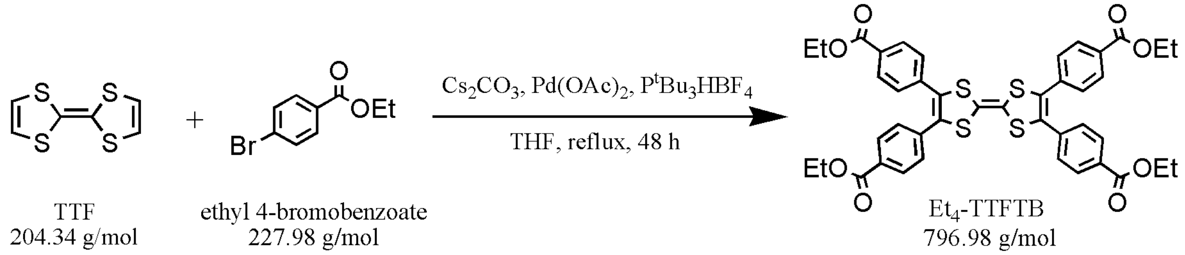

Scheme S1. Synthesis of **Et_4-TTFTB**.

$Pd(OAc)_2$ (84 mg, 0.38 mmol), $P^tBu_3 \cdot HBF_4$ (326 mg, 1.1 mmol), and Cs_2CO_3 (2444 mg, 7.5 mmol) were placed in a 50 mL reaction flask under argon. THF (20 mL) was added and the mixture was stirred for 10 min with heating. A solution of tetrathiafulvalene (307 mg, 1.5 mmol) and ethyl 4-bromobenzoate (1718 mg, 7.5 mmol) in THF (20 mL) was added. The mixture was heated at reflux for 48 h. The organic compounds were extracted with chloroform three times. The combined organic part was washed with brine, dried over anhydrous Na_2SO_4, and concentrated in vacuum. Chromatographic purification on silica gel by using chloroform as an eluent afforded 2,3,6,7-tetra-(2-ethoxycarbonylbenzene) tetrathiafulvalene (**Et_4-TTFTB**) (840 mg, 1.1 mmol, 73 %) as a dark red solid. Characterization: ^{1}H NMR ($CDCl_3$): δ/ppm = 1.37 (t, J = 7.1 Hz, 12H, $CO_2CH_2C\mathbf{H}_3$), 4.36 (q, J = 7.1 Hz, 8H, $CO_2C\mathbf{H}_2CH_3$), 7.25–7.27 (m, 8H), 7.91 (dt, J = 8.7, 1.8 Hz, 8H). IR (ν/cm^{-1}): 1716 (s, C=O); 1696 (m); 1275 (s); 1101 (s).

1.2. Synthesis of NaH_3TTFTB

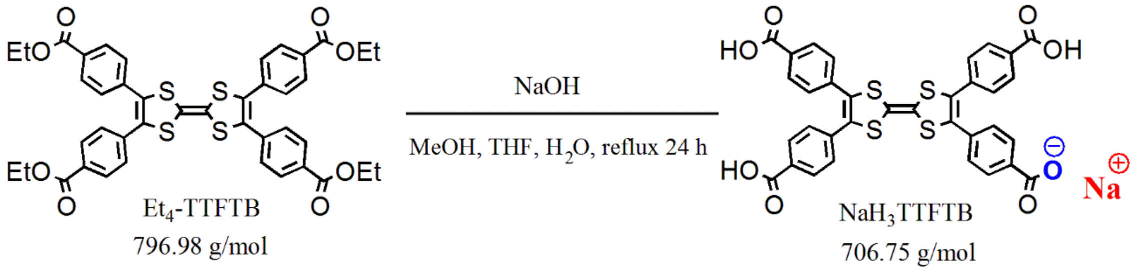

Scheme S2. Synthesis of **NaH_3TTFTB**.

A 50 mL flask was charged with **Et_4-TTFTB** (840 mg, 1.1 mmol) and subjected to three cycles of evacuation and refilling with argon. MeOH (10 mL) and THF (10 mL) were added to generate a suspension. In a separate flask, NaOH (558 mg) was dissolved in degassed water (10 mL). The sodium hydroxide solution was added to Et_4-TTFTB under argon and the reaction was heated to reflux for 24 hours. The reaction was then cooled to room temperature and the volatiles were

removed in vacuum. A 1 M solution of HCl (30 mL) was added to afford a maroon precipitate, which was collected by filtration and washed with water (50 mL). The product was collected and dried under high vacuum for 12 hours to afford **NaH_3TTFTB** as a maroon solid (739 mg, 0.968 mmol, 88% yield). Characterization: ^{1}H NMR (DMSO-d_6): δ/ppm = 13.14 (br s, 3H, CO_2**H**), 7.87 (dt, 8H, J = 8.3 Hz, 1.8 Hz), 7.35 (dt, 8H, J =8.4 Hz, 1.8 Hz). IR (ν/cm^{-1}): 3420 (s, OH); 1686 (s, C=O); 1602 (s); 1412 (m); 1265 (m). EDAX mapping shows a homogenous distribution of Na along the solid (Figure S2). In addition, the ratio Na:S is in agreement with the presence of 1 Na^+ cation per TTFTB moiety (theoretical S:Na ratio = 4:1, found S:Na ratio = 4.3:1, see Table S1).

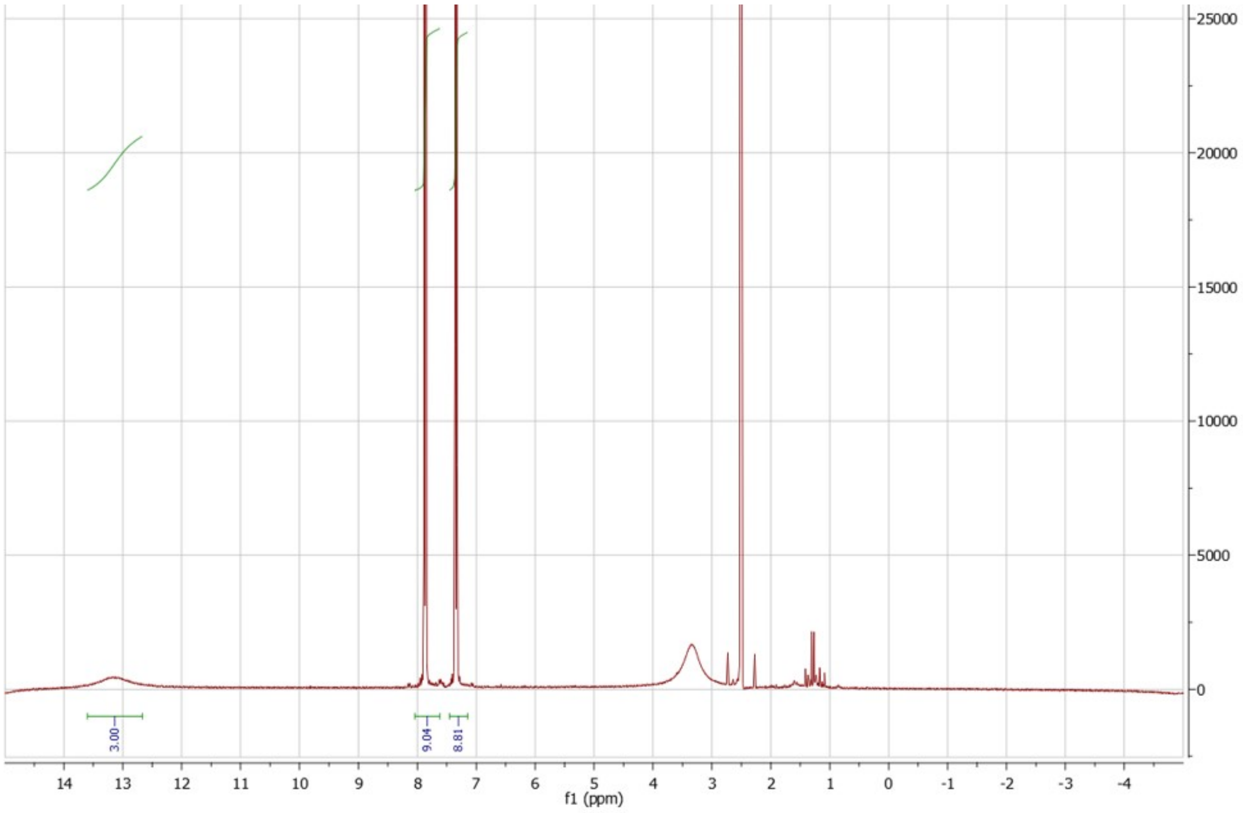

Figure S1. ^{1}H NMR of **NaH_3TTFTB** in DMSO-d_6.

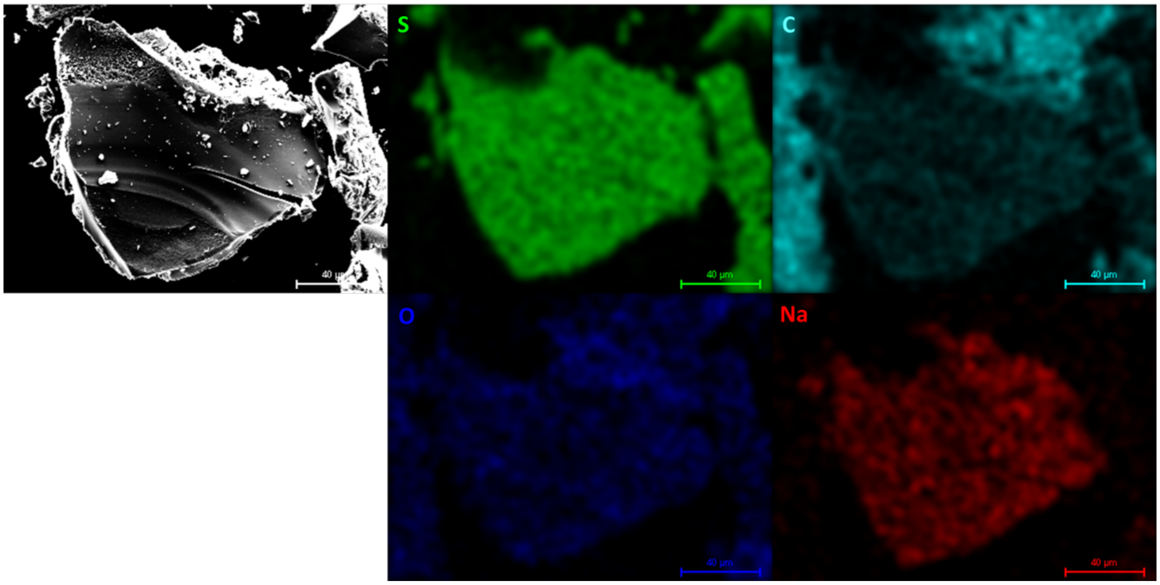

Figure S2. EDAX mapping of a crystal of the precursor NaH_3TTFTB.

Table S1 Sulfur and sodium percentage inferred from EDAX analysis of different samples (**1-6**) of the precursor NaH_3TTFTB.

	**1**	**2**	**3**	**4**	**5**	**6**
**Sulfur**	7.35	2.11	2.39	5.18	7.65	3.08
**Sodium**	1.9	0.44	0.49	1.5	1.63	0.87
**Ratio S:Na**	**3.868421**	**4.795455**	**4.877551**	**3.453333**	**4.693252**	**3.54023**
**Mean**	**4.28 ± 0.65**					

1.3. Crystallization of MUV-20a.

2 mL of tetrahydrofuran (THF) were added to 20 mg of **NaH_3TTFTB** in a 40 mL vial. The mixture was sonicated for 2 minutes until a transparent dark red solution was formed. This solution was heated for 5 min at 80 ºC by placing the vial on top of a heating plate. Then, 30 mL of diethyl ether were added, resulting in red needle-like single crystals of **MUV-20a**, after few minutes at room temperature, in which THF molecules are embedded in the pores (Figure S11). Finally, the crystals were filtered at air (12 mg, yield 60%).

1.4. Crystallization of MUV-20b.

Upon formation of red needle-like single crystals of **MUV-20a,** these were washed with diethyl ether, yielding red needle-like single crystals of **MUV-20b** (16 mg, yield 80%), in which THF molecules are replaced by diethyl ether molecules (Figure S19).

1.5. Crystallization of MUV-21.

20 mg of **NaH_3TTFTB** were solved with 1 mL of DMF in a 4 mL vial at 105 ºC during 60 hours into the oven with heating and cooling ramps of 0.5 ºC min^{-1}, resulting in red needle-like single crystals of **MUV-21** that were washed with diethyl ether at room temperature (4 mg, yield: 20 %). **MUV-21** can also be prepared using the same synthetic conditions but starting with **H_4TTFTB**.

2. Crystal structures

2.1. Crystallographic information.

Data Collection. X-ray data for compound **MUV-20a** were collected at beamline I19 in Diamond Light Source synchrotron at a temperature of 100 K using a Pilatus 6M detector.[1] X-ray data for compound **MUV-20b** were collected at 100 K using a Rigaku FR-X rotating anode with a Hypix 6000HE detector. X-ray data for compound **MUV-21** were collected at 120 K using a Rigaku microfocus Supernova with an Atlas CCD detector. Data were measured using GDA and CrysAlisPro suite of programs.

Crystal structure determinations and refinements. X-ray data were processed and reduced using the CrysAlisPro suite of programs. Absorption correction was performed using empirical methods (SCALE3 ABSPACK) based upon symmetry-equivalent reflections combined with measurements at different azimuthal angles. The crystal structures were solved and refined against all F^2 values using the SHELX and Olex 2 suite of programs.[2,3]All atoms were refined anysotropically with the exception of the partially occupied diethyl ether molecule in **MUV-20b** and a solvent DMF in **MUV-21**. Hydrogen atoms were placed in the calculated positions.

Crystal structure of MUV-20a. Diffraction data from compound **MUV-20a** presented a large mosaicity at high angle and overloads at low angle. The ill-shaped and overloaded reflexions were excluded from the final data, obtaining a lower than ideal completeness (89%). THF solvent molecules were found disordered and modelled over two positions. C–O and C–C distances were restrained using same distance (SADI) and distance fix (DFIX) SHELX restrains. The atomic displacement parameters (adp) were restrained using rigid body restrains (SHELX RIGU and SIMU commands). **MUV-20a** was modelled with three carboxylic acids and a carboxylate, where O1 is deprotonated, despite that C1–O1 and C1–O2 atomic distances were also consistent with the presence of a carboxylic group. This particular carboxylate group was chosen to be deprotonated due to the lack of possible hydrogen bond interactions. This model was also chosen to match the EPR data and theoretical structures obtained.

Crystal structure of MUV-20b. Disordered diethyl ether molecules were modelled over a special position and the overall occupancy was refined. Diethyl ether molecules were refined as a rigid body using idealized geometry for the C–C and C–O bonds. The atomic displacement parameters (adp) were restrained using rigid body restrains (SHELX RIGU and SIMU commands). **MUV-20b** was also modelled with three carboxylic acids and a carboxylate group, where O1 is deprotonated, despite that C1–O1 and C1–O2 atomic distances were also consistent with the presence of a carboxylic group. This model was chosen to match the EPR data and theoretical models obtained.

Crystal structure of MUV-21. Despite of the usage of a highly intense X-ray source, crystals of **MUV-21** only diffracted to 1 Å of resolution. Crystal structure of **MUV-21** was modelled as an anion with a carboxylic group disordered over two positions, forming hydrogen bonds with two disordered dimethylamonium cations and a carboxylate group of a neighbouring molecule. A disordered DMF solvent molecule was also found and modelled over two positions. C–O, C–N and C–C distances were restrained using same distance (SADI) and distance fix (DFIX) SHELX restrains. The atomic displacement parameters (adp) were restrained using rigid body restrains (S,HELX RIGU and SIMU commands). The carboxylic hydrogens were disordered over the two oxygen atoms in two carboxylic groups in order to form a sensible hydrogen-bonded network. Solvent mask protocol implemented in OLEX2 was used to account with the remaining electron density in the pores of **MUV-21**, finding 29 electrons which could correspond to 0.75 DMF molecules per formula unit.

CCDC 2153374–2153376 contain the supplementary crystallographic data for this paper. These data can be obtained free of charge via www.ccdc.cam.ac.uk/conts/retrieving.html (or from the Cambridge Crystallographic Data Centre, 12 Union Road, Cambridge CB21EZ, UK; fax: (+44)1223-336-033; or deposit@ccdc.cam.ac.uk).

Table S2. Crystallographic information of compounds **MUV-20a**, **MUV-20b**, and **MUV-21**

Identification code	**MUV20a**	**MUV20b**	**MUV21**
Empirical formula	$C_{38}H_{27}O_9S_4$	$C_{35.58}H_{26.89}O_{8.39}S_4$	$C_{40.8}H_{38.19}N_{2.6}O_{9.6}S_4$
Formula weight	755.83	716.98	846.70
Temperature/K	293(5)	100(2)	119(2)
Crystal system	triclinic	triclinic	triclinic
Space group, Z	*P*-1, 2	*P*-1, 2	*P*-1, 2
a/Å	10.0563(7)	10.3419(14)	8.8853(7)
b/Å	11.5820(8)	12.3660(19)	18.4027(9)
c/Å	15.9935(10)	15.222(2)	19.3889(18)
α/°	92.553(6)	86.172(13)	114.461(7)
β/°	107.690(6)	70.626(13)	95.946(7)
γ/°	102.434(6)	66.203(14)	93.308(5)
Volume/Å^3	1721.0(2)	1675.2(5)	2852.5(4)
ρ_{calc}/g cm^{-3}	1.459	1.421	0.986
μ/mm^{-1}	0.309	3.062	0.209
F(000)	782.0	743.0	884.0
Crystal size/mm^3	0.19 × 0.15 × 0.06	0.25 × 0.05 × 0.05	0.32 × 0.04 × 0.04
Radiation	synchrotron (*λ* = 0.6889)	Cu Kα (*λ* = 1.54184)	Mo Kα (*λ* = 0.71073)
2Θ range for data collection/°	3.516 to 49.028	6.174 to 151.85	6.568 to 41.63
Index ranges	$-12 \le h \le 12$, $-13 \le k \le 13$, $-19 \le l \le 19$	$-8 \le h \le 12$, $-15 \le k \le 15$, $-18 \le l \le 18$	$-8 \le h \le 8$, $-18 \le k \le 18$, $-19 \le l \le 19$
Reflections collected	11455	16022	25915
Independent reflections	5612 [R_{int} = 0.0462, R_{sigma} = 0.0388]	6501 [R_{int} = 0.0481, R_{sigma} = 0.0568]	5939 [R_{int} = 0.1208, R_{sigma} = 0.1146]
Data/restraints/parameters	5612/249/509	6501/7/432	5939/731/603
Goodness-of-fit on F^2	1.062	1.028	1.045
Final R indexes [I>=2σ (I)]	R_1 = 0.1137, wR_2 = 0.2981	R_1 = 0.0776, wR_2 = 0.2058	R_1 = 0.0880, wR_2 = 0.2447
Final R indexes [all data]	R_1 = 0.1351, wR_2 = 0.3182	R_1 = 0.1154, wR_2 = 0.2498	R_1 = 0.1256, wR_2 = 0.2806
Largest diff. peak/hole / e Å^{-3}	1.43/–0.81	1.12/–0.92	0.64/–0.42

2.2. Crystal structure of MUV-20a

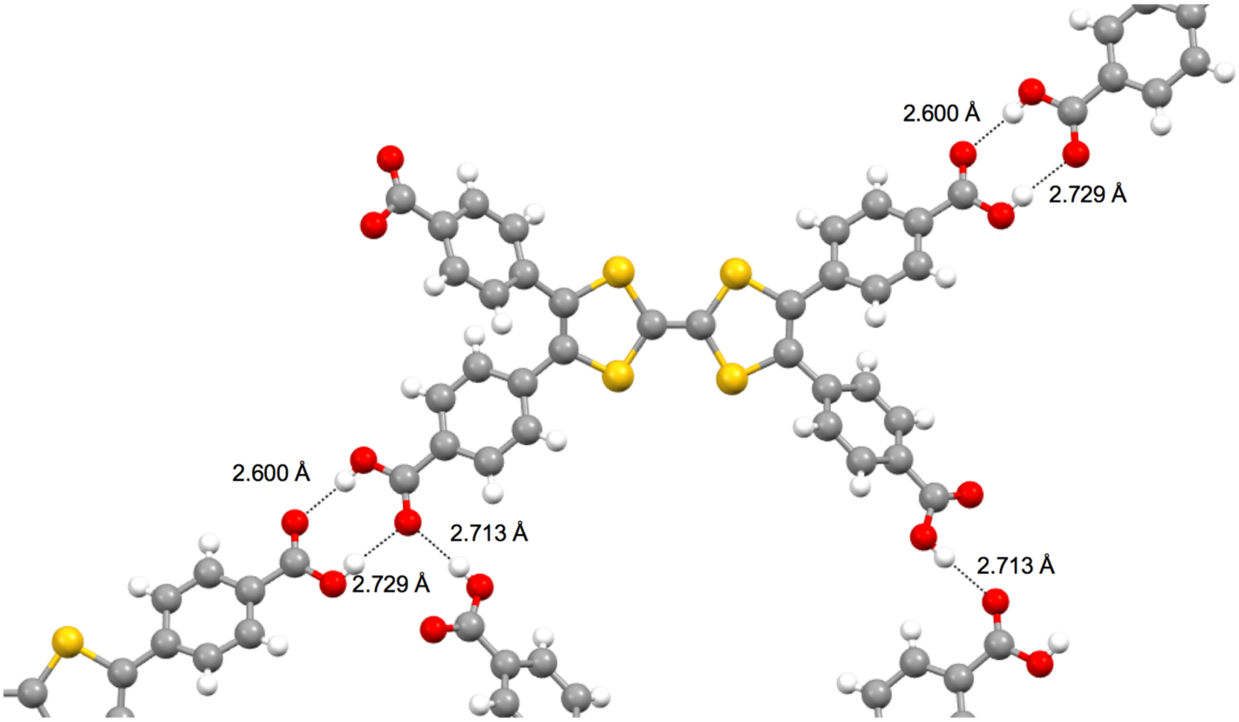

Figure S3. Structural details in **MUV-20a**, showing the H-bonding of three of the four carboxylates (O···O distances between carboxylic groups are indicated).

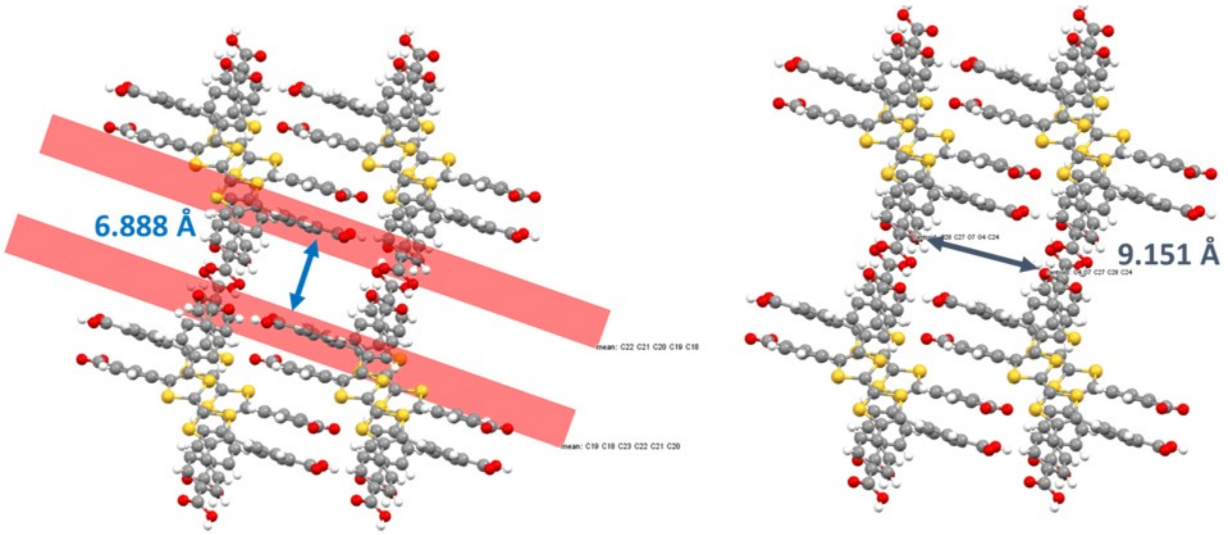

Figure S4. Pore dimensions for **MUV-20a**: (left) distance between average planes of parallel benzenes; (right) distance between calculated centroids.

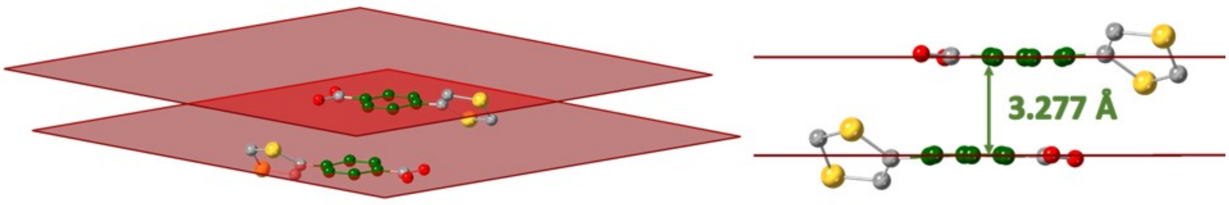

Figure S5. π–π stacking distance between benzene planes from **MUV-20a.**

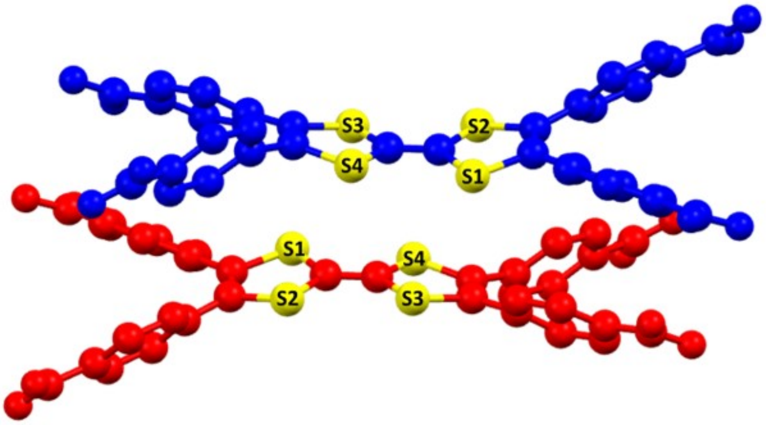

Figure S6. Representation of closest neighboring layers in **MUV-20a** with the sulphur labelling (S···S distances summarized in Table S3).

Table S3. Distances (in Å) between sulphur atoms (S···S) from closest neighboring layers in **MUV-20a**.

**S1···S1**	7.002	**S2···S1**	5.900	**S3···S1**	3.978	**S4···S1**	5.215
**S1···S2**	5.900	**S2···S2**	6.161	**S3···S2**	4.384	**S4···S2**	**3.658**
**S1···S3**	3.978	**S2···S3**	4.384	**S3···S3**	4.568	**S4···S3**	**3.910**
**S1···S4**	5.215	**S2···S4**	**3.658**	**S3···S4**	**3.910**	**S4···S4**	5.195

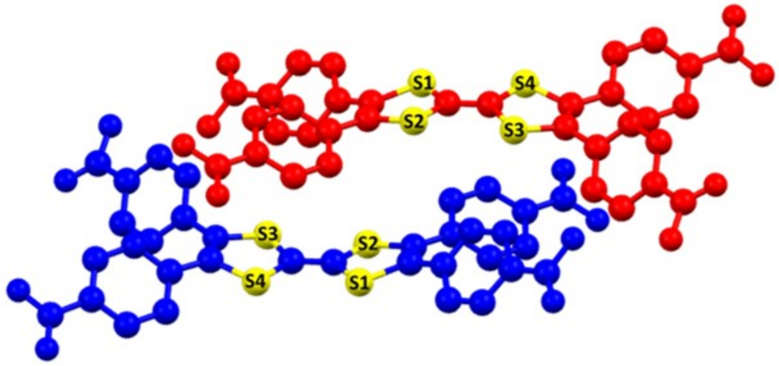

Figure S7. Representation of furthest neighboring layers in **MUV-20a** with the sulphur labelling (S···S distances summarized in Table S4).

Table S4. Distances (in Å) between sulphur atoms (S···S) from furthest neighboring layers in **MUV-20a**.

**S1···S1**	7.555	**S2···S1**	5.467	**S3···S1**	6.899	**S4···S1**	8.819
**S1···S2**	5.467	**S2···S2**	**4.447**	**S3···S2**	6.165	**S4···S2**	7.140
**S1···S3**	6.899	**S2···S3**	6.165	**S3···S3**	8.738	**S4···S3**	9.465
**S1···S4**	8.819	**S2···S4**	7.140	**S3···S4**	9.465	**S4···S4**	10.959

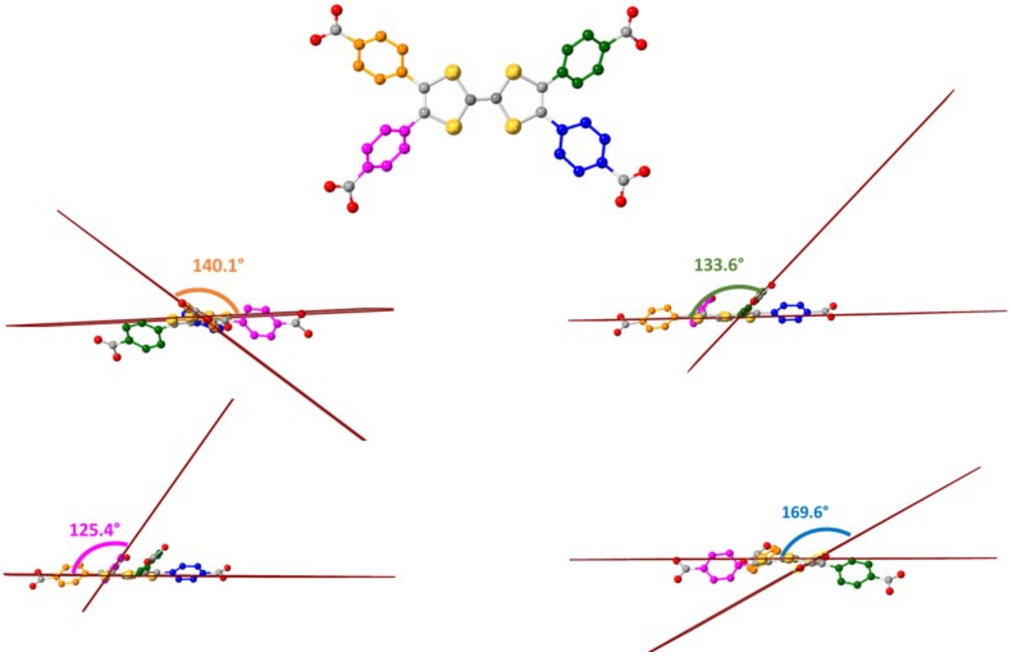

Figure S8. Angles formed by the average planes computed for benzene groups and the TTF moiety in **MUV-20a**.

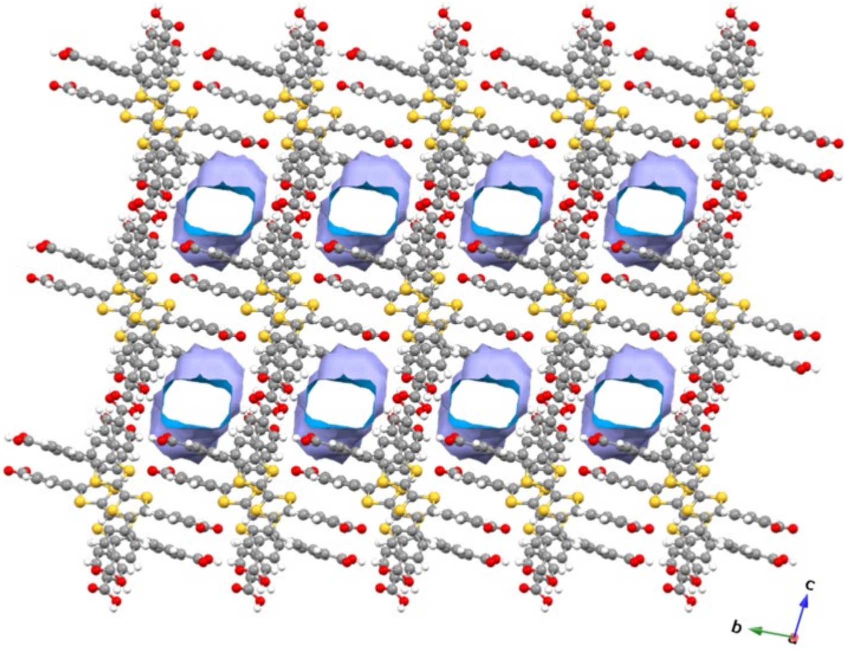

Figure S9. Void space in **MUV-20a** seen along the *a* axis.

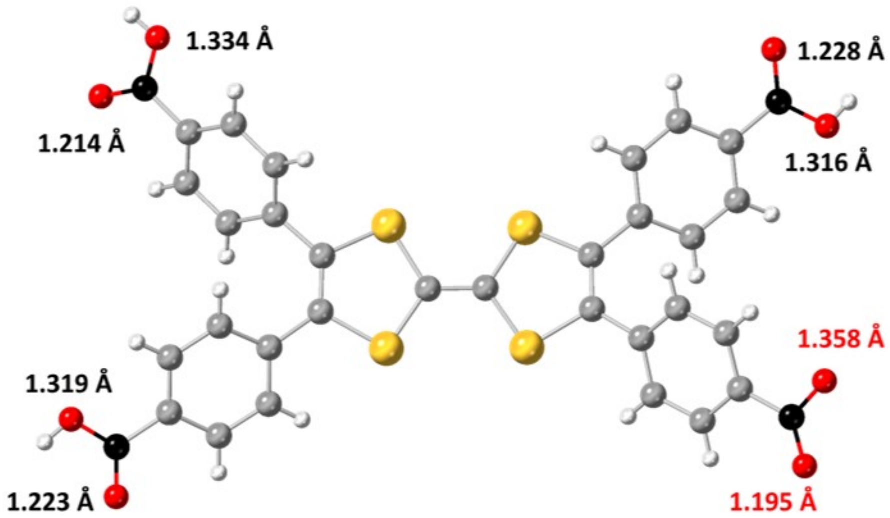

Figure S10. C–O distances of the carboxylic/carboxylate groups in **MUV-20a**.

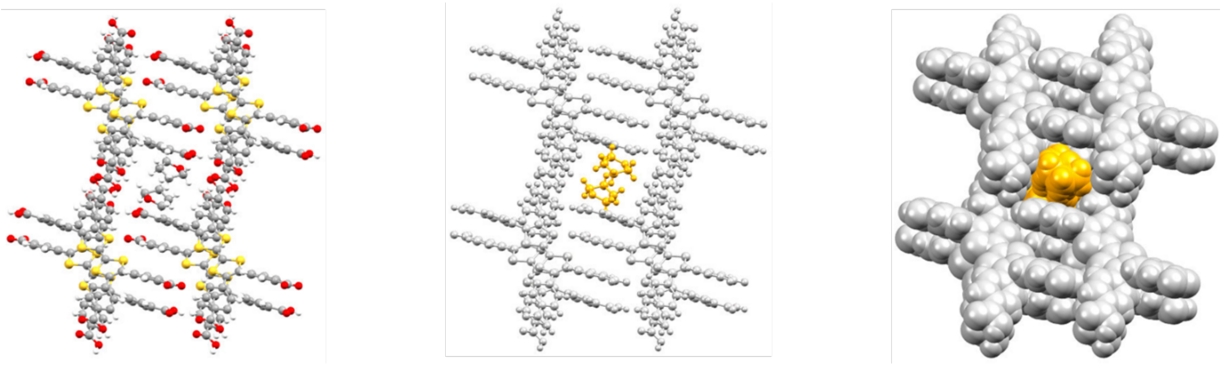

Figure S11. Crystal structure of as-synthetized **MUV-20a**, highlighting the presence of THF molecules in the pores.

2.3. Crystal structure of MUV-20b

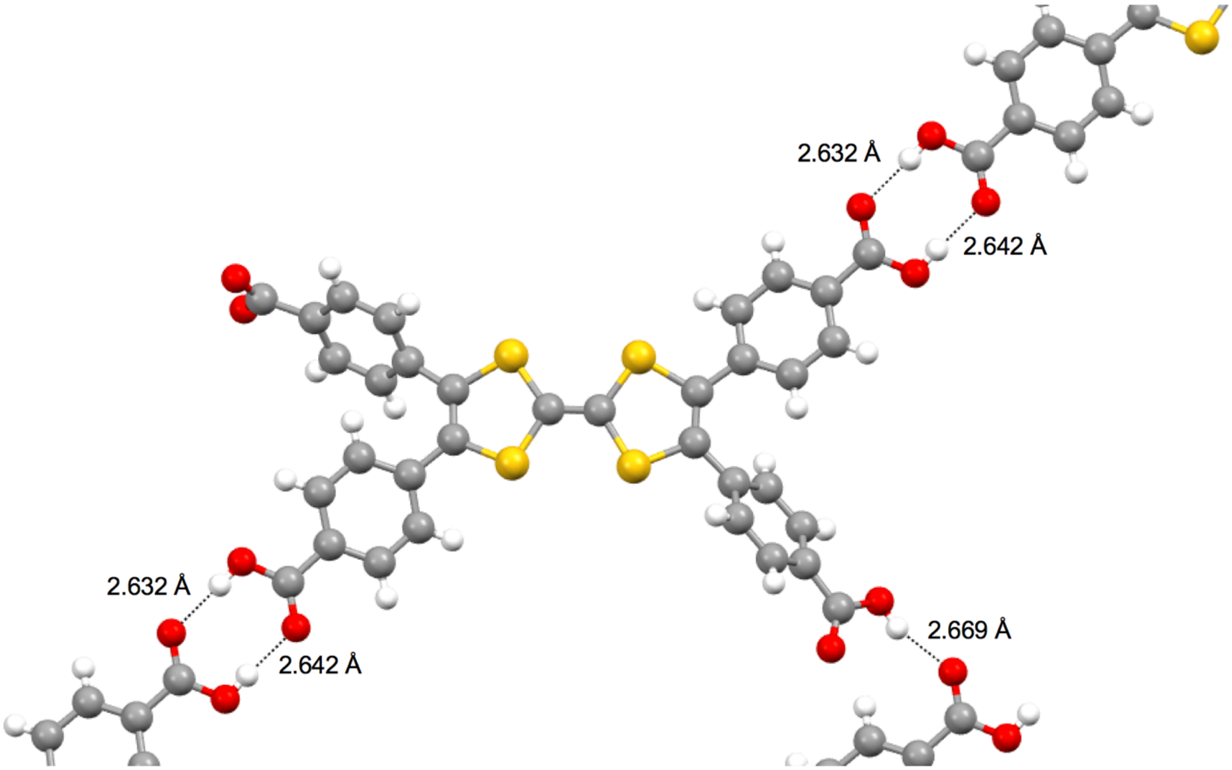

Figure S12. Structural details in **MUV-20b**, showing the H-bonding of three of the four carboxylates (O···O distances between carboxylic groups indicated).

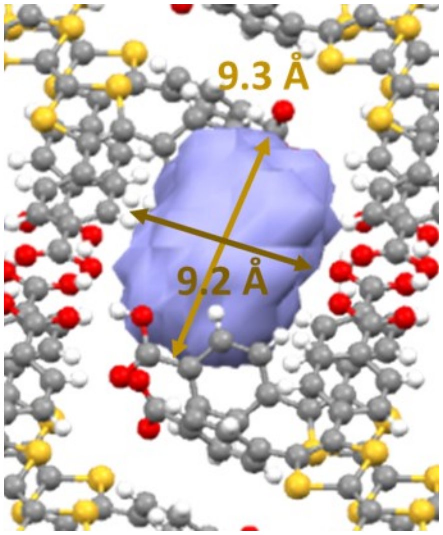

Figure S13. Pore dimensions for **MUV-20b.**

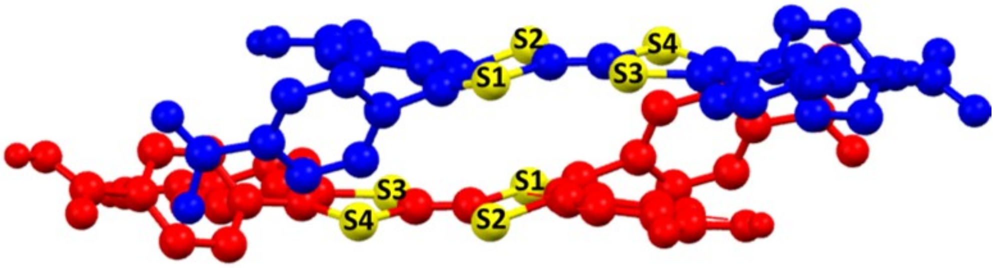

Figure S14. Representation of closest neighboring layers in **MUV-20b** with the sulphur labelling (S···S distances summarized in Table S5).

Table S5. Distances (in Å)between sulphur atoms (S···S) from closest neighboring layers in **MUV-20b**.

**S1···S1**	6.639	**S2···S1**	5.995	**S3···S1**	4.562	**S4···S1**	3.873
**S1···S2**	5.995	**S2···S2**	6.722	**S3···S2**	**3.638**	**S4···S2**	4.972
**S1···S3**	4.562	**S2···S3**	**3.638**	**S3···S3**	4.439	**S4···S3**	**3.660**
**S1···S4**	3.873	**S2···S4**	4.972	**S3···S4**	**3.660**	**S4···S4**	4.941

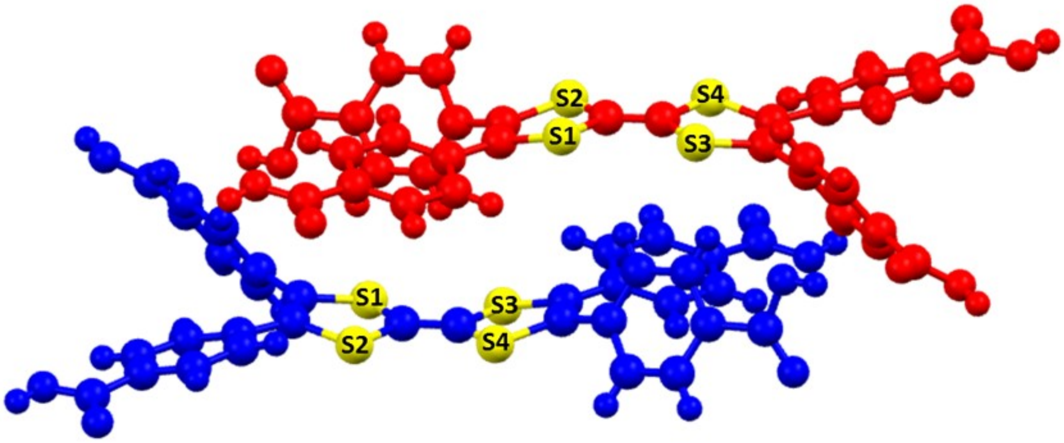

Figure S15. Representation of furthest neighboring layers in **MUV-20b** with the sulphur labelling (S···S distances summarized in Table S6).

Table S6. Distances (in Å) between sulphur atoms (S···S) from furthest neighboring layers in **MUV-20b**.

**S1···S1**	7.363	**S2···S1**	5.321	**S3···S1**	8.624	**S4···S1**	6.790
**S1···S2**	5.321	**S2···S2**	**4.448**	**S3···S2**	7.003	**S4···S2**	6.170
**S1···S3**	8.624	**S2···S3**	7.003	**S3···S3**	10.803	**S4···S3**	9.377
**S1···S4**	6.790	**S2···S4**	6.170	**S3···S4**	9.377	**S4···S4**	8.746

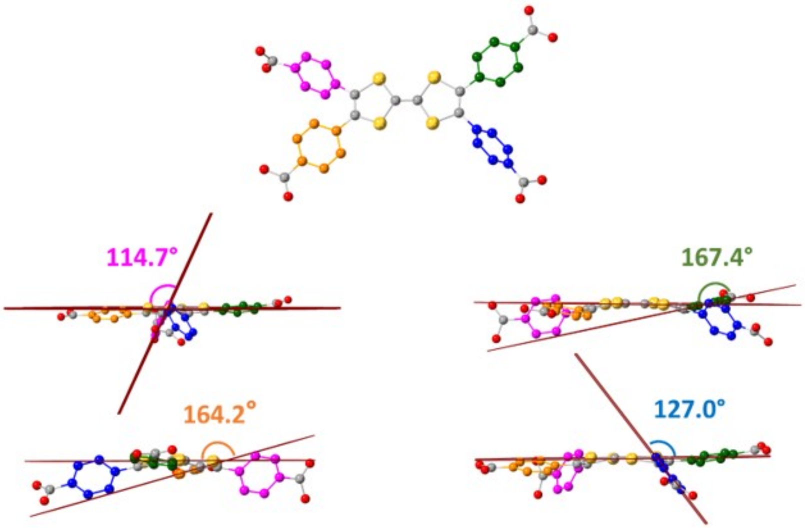

Figure S16. Angles formed by the average planes computed for benzene groups and the TTF moiety in **MUV-20b**.

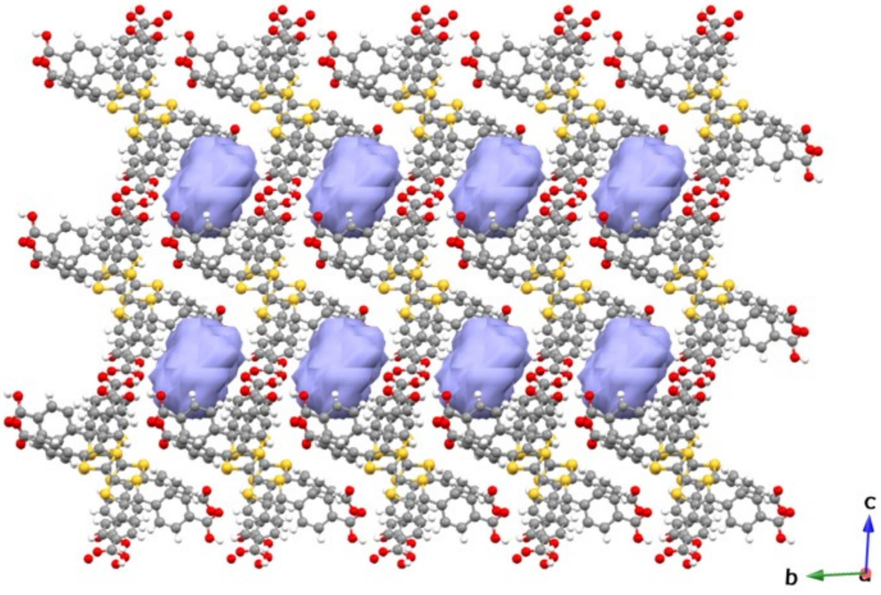

Figure S17. Void space in **MUV-20b** seen along the *a* axis.

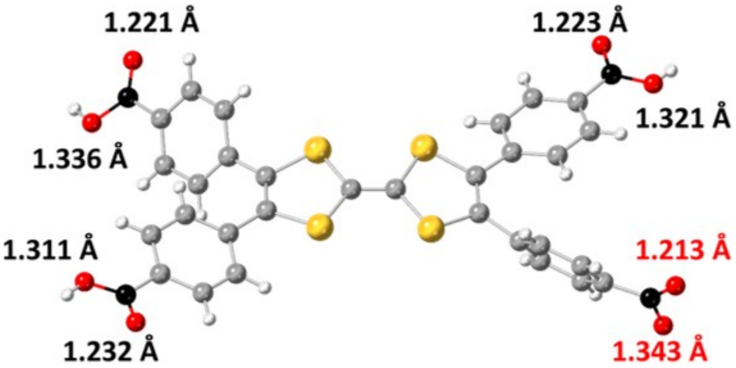

Figure S18. C–O distances of the carboxylic/carboxylate groups in **MUV-20b**.

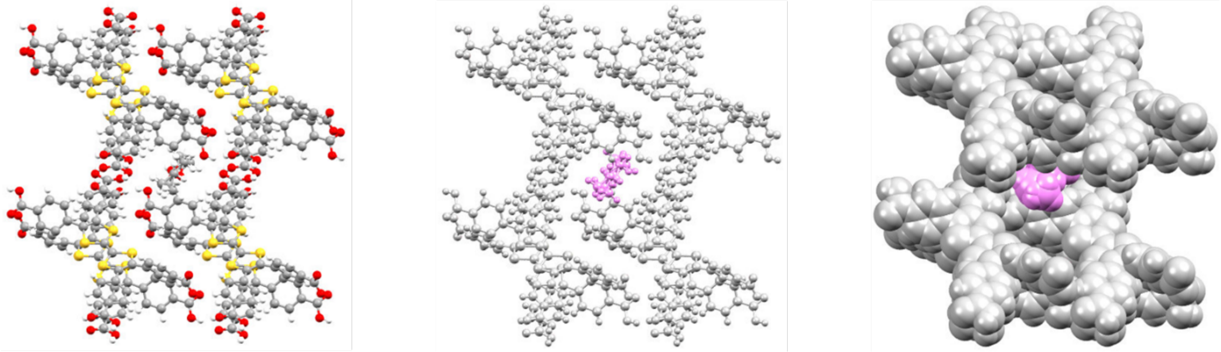

Figure S19. Crystal structure of as-synthetized **MUV-20b**, highlighting the presence of diethyl ether molecules in the pores.

2.4. Crystal structure of MUV-21

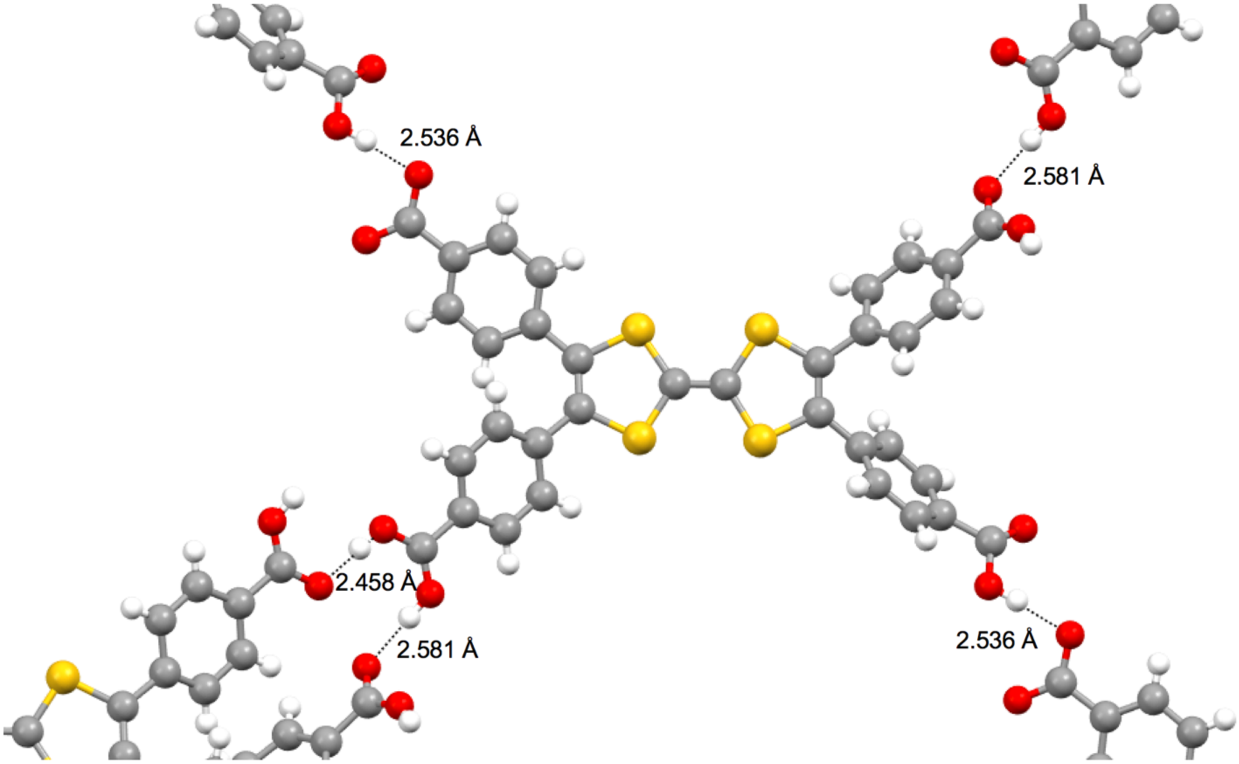

Figure S20. Structural details in **MUV-21**, showing the H-bonding of the four carboxylates (O···O distances between carboxylic groups indicated).

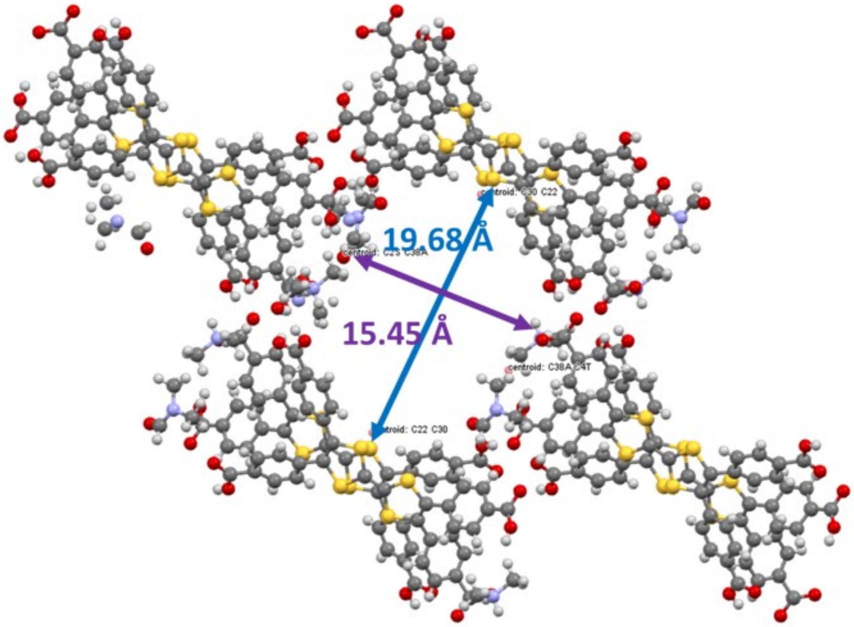

Figure S21. Pore dimensions for **MUV-21.**

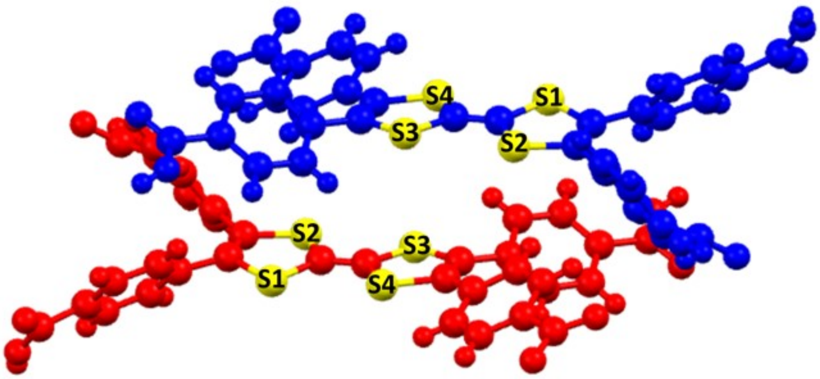

Figure S22. Representation of closest neighboring layers in **MUV-21** with the sulphur labelling (S···S distances summarized in Table S7).

Table S7. Distances (in Å) between sulphur atoms (S···S) from closest neighboring layers in **MUV-21**.

**S1···S1**	6.440	**S2···S1**	7.089	**S3···S1**	4.954	**S4···S1**	4.144
**S1···S2**	7.089	**S2···S2**	8.745	**S3···S2**	7.134	**S4···S2**	5.126
**S1···S3**	4.954	**S2···S3**	7.134	**S3···S3**	6.774	**S4···S3**	4.633
**S1···S4**	4.144	**S2···S4**	5.126	**S3···S4**	4.633	**S4···S4**	**3.771**

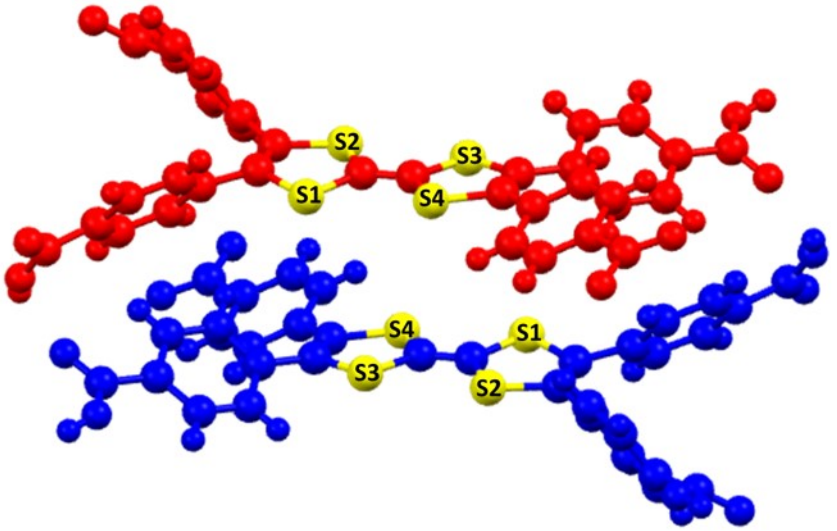

Figure S23. Representation of furthest neighboring layers in **MUV-21** with the sulphur labelling (S···S distances summarized in Table S8).

Table S8. Distances (in Å) between sulphur atoms (S···S) between furthest neighboring layers in **MUV-21**.

**S1···S1**	9.277	**S2···S1**	7.883	**S3···S1**	5.579	**S4···S1**	7.230
**S1···S2**	7.883	**S2···S2**	7.462	**S3···S2**	4.978	**S4···S2**	5.356
**S1···S3**	5.519	**S2···S3**	4.978	**S3···S3**	**3.802**	**S4···S3**	4.307
**S1···S4**	7.230	**S2···S4**	5.356	**S3···S4**	4.307	**S4···S4**	6.311

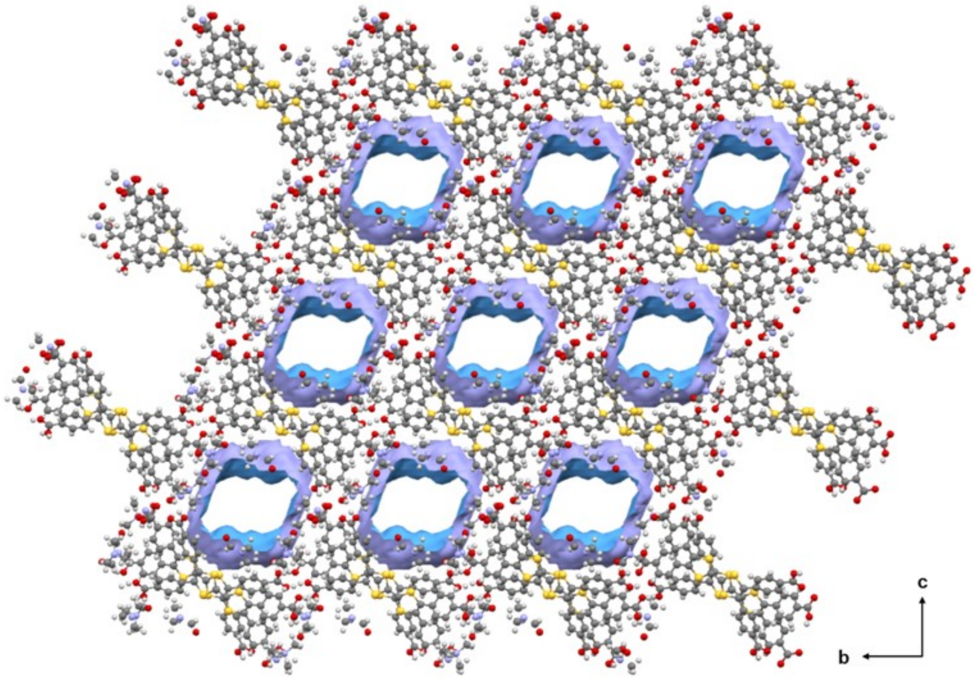

Figure S24. Void space in **MUV-21** seen along the *a* axis.

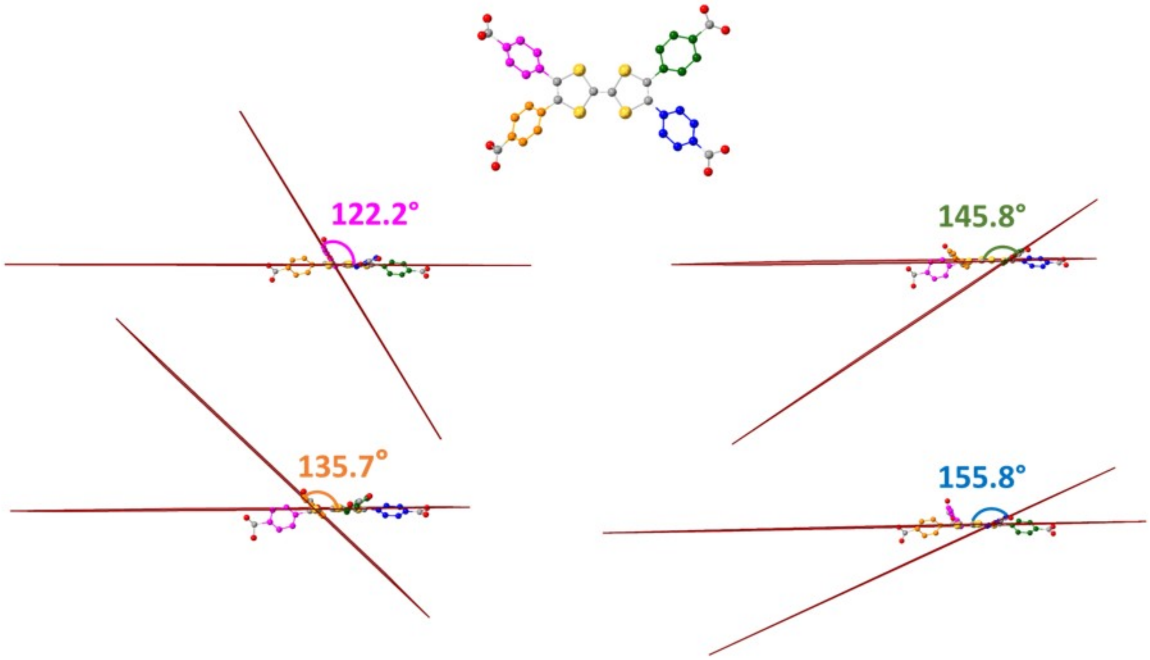

Figure S25. Angles formed by the average planes computed for benzene groups and the TTF moiety in **MUV-21**.

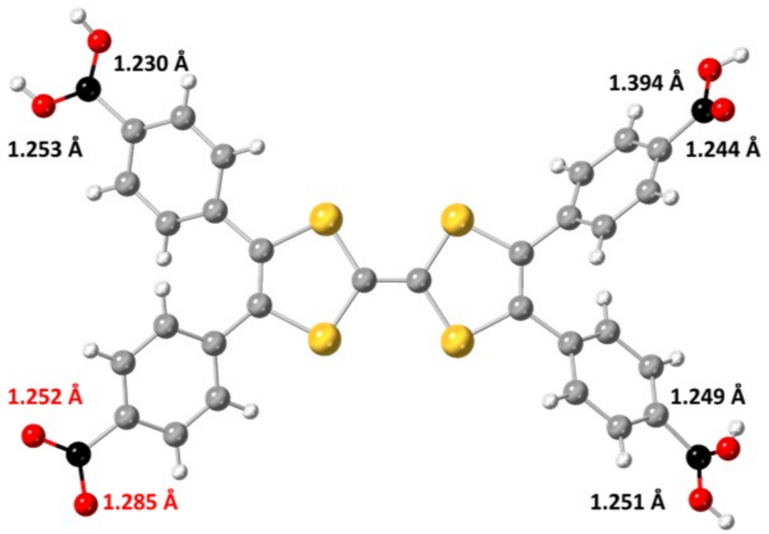

Figure S26. C–O distances of the carboxylic/carboxylate groups in **MUV-21**.

3. Infrared spectroscopy (IR)

Infrared spectra were recorded in a Platinum-ATR diamond Bruker spectrometer in the 4000–400 cm^{-1} range using microcrystalline powder.

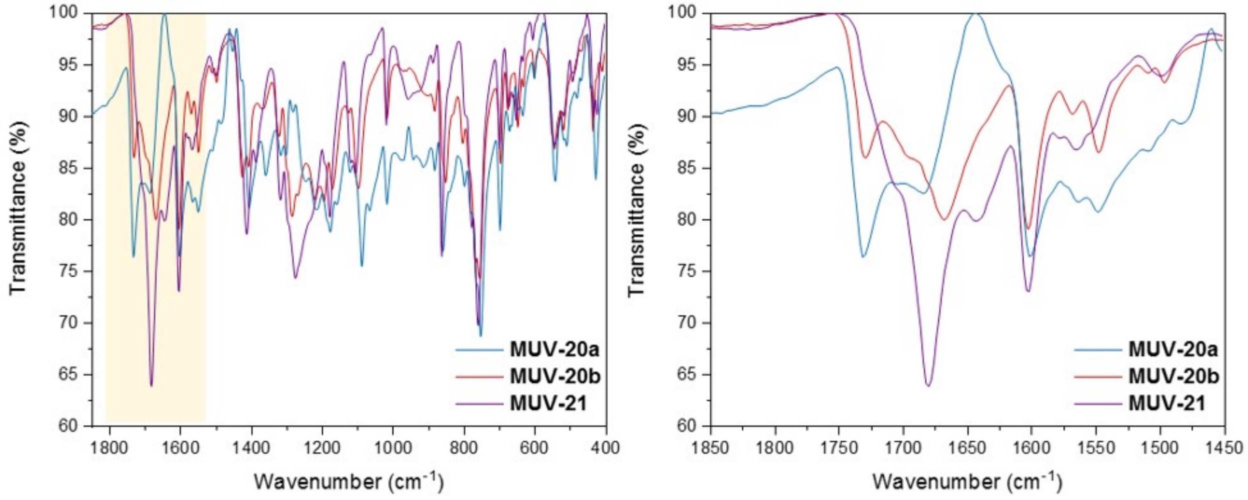

Figure S27. Infrared spectra of **MUV-20a** (blue), **MUV-20b** (red), and **MUV-21** (purple).

4. Powder X-ray diffraction (PXRD)

Powder X-ray diffraction spectra were recorded using 0.7 mm borosilicate capillaries that were aligned on an Empyrean PANalytical powder diffractometer, using Cu Ka radiation (λ = 1.54056 Å) with a PIXcel detector.

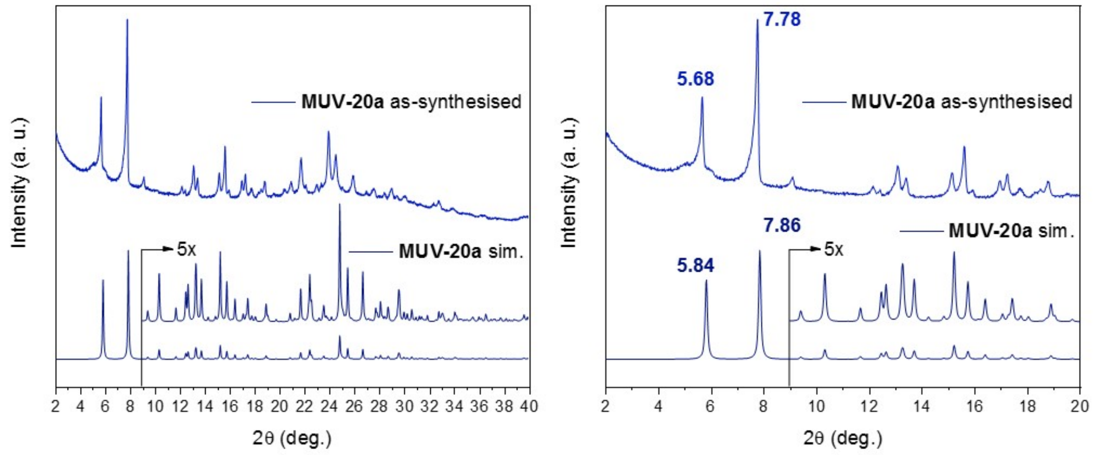

Figure S28. PXRD patterns of as-synthesized **MUV-20a** and simulated (from single crystal data).

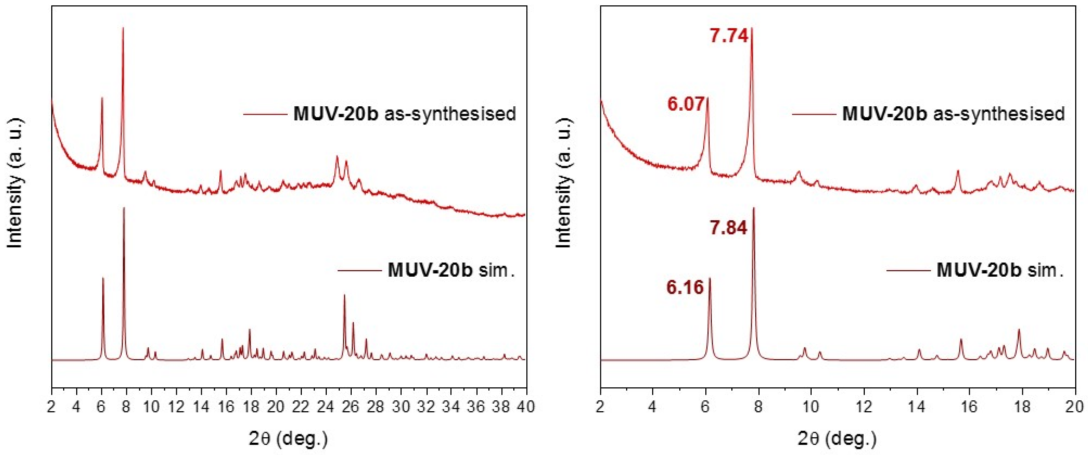

Figure S29. PXRD patterns of as-synthesized **MUV-20b** and simulated (from single crystal data).

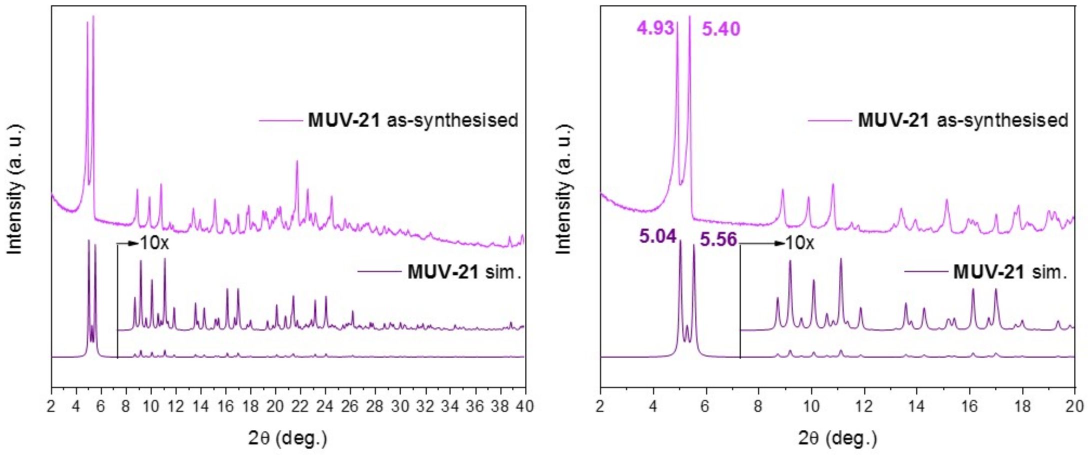

Figure S30. PXRD patterns of as-synthesized **MUV-21** and simulated (from single crystal data).

Washing crystals of **MUV-20a** with diethyl ether causes the transformation to **MUV-20b** (Figure S31)

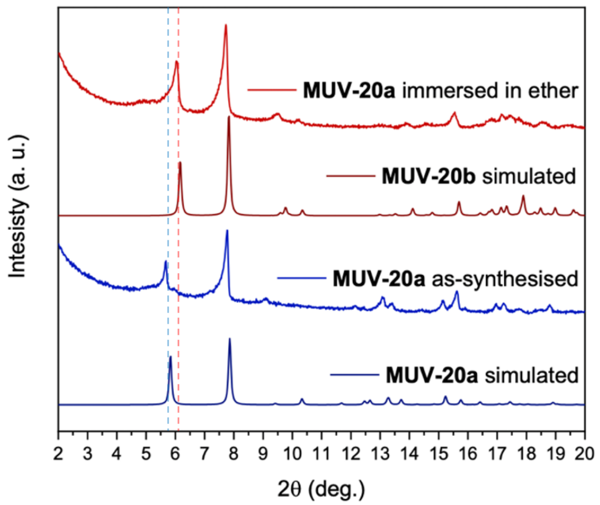

Figure S31. X-ray powder diffraction pattern of as-synthetised **MUV-20a** (in red) and after washing with diethyl ether, converting to **MUV-20b** (in blue).

The chemical stabilities of **MUV-20a**, **MUV-20b** and **MUV-21** were studied by immersing the solids in different solvents for 30 min. As can be seen in Figures S32, S33 and S34, **MUV-20a** and **MUV-20b** are stable in water, but not in the other solvents tested (EtOH, hexane, ethyl acetate and acetonitrile). On the contrary, **MUV-21** is stable in acetonitrile, hexane and ether, but not in water, ethyl acetate and EtOH.

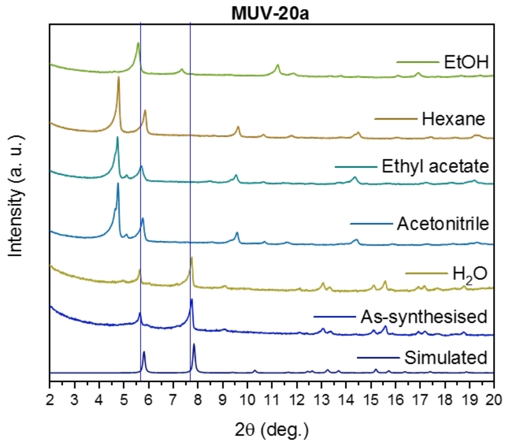

Figure S32. XRPD of **MUV-20a** after immersion in different solvents.

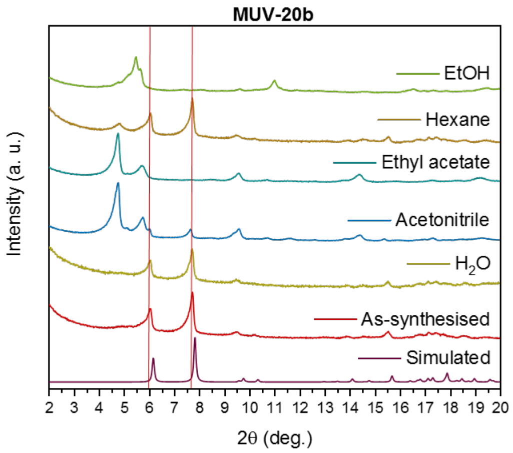

Figure S33. XRPD of **MUV-20b** after immersion in different solvents.

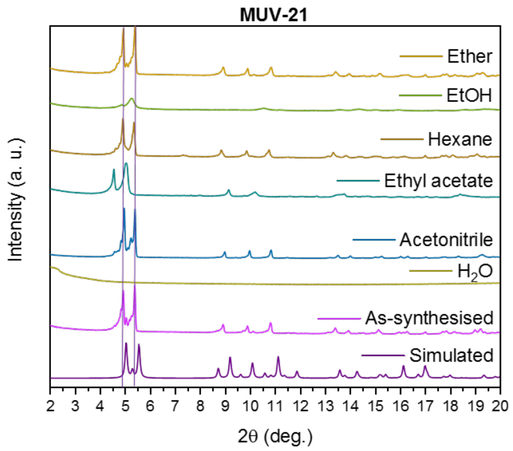

Figure S34. XRPD of **MUV-21** after immersion in different solvents.

5. Thermogravimetric analysis

Thermogravimetric analysis was carried out with a Mettler Toledo TGA/SDTA 851 apparatus in the 25–700 ºC temperature range under 5 ºC·min^{-1} scan rate and an air flow of 30 mL·min^{-1}.

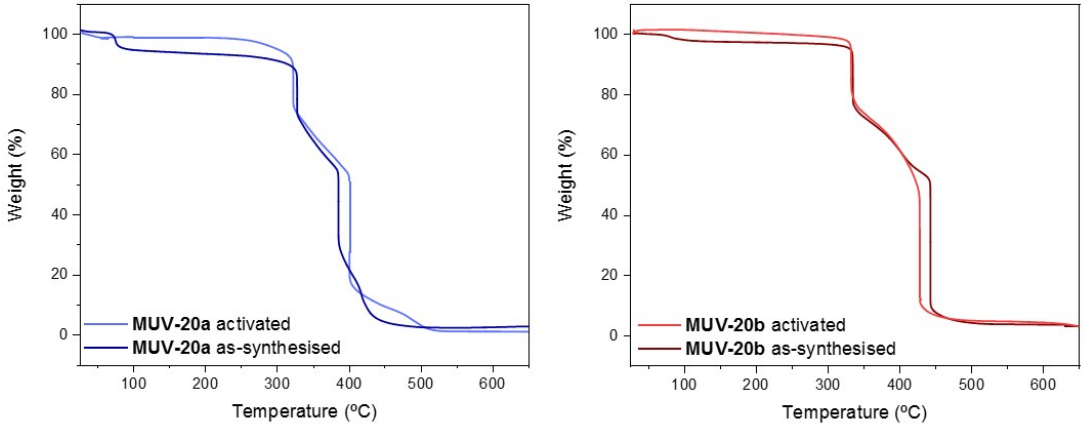

Figure S35. TGA of **MUV-20a** (left) and **MUV-20b** (right).

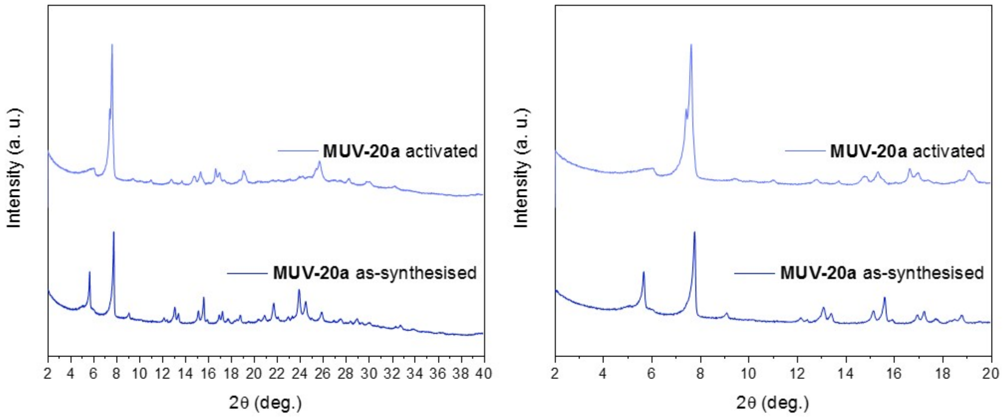

Figure S36. PXRD patterns of as-synthesised **MUV-20a** and after activation (1 hour heating at 70 ºC).

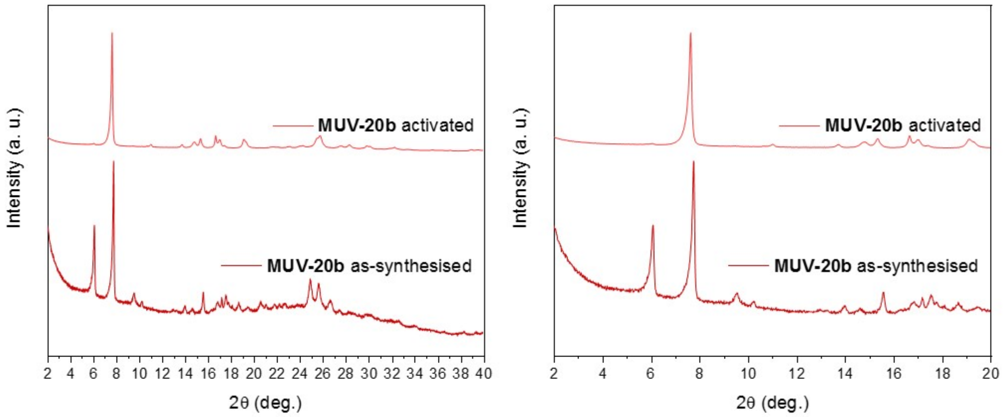

Figure S37. PXRD patterns of as-synthesised **MUV-20b** and after activation (1 hour heating at 70 ºC).

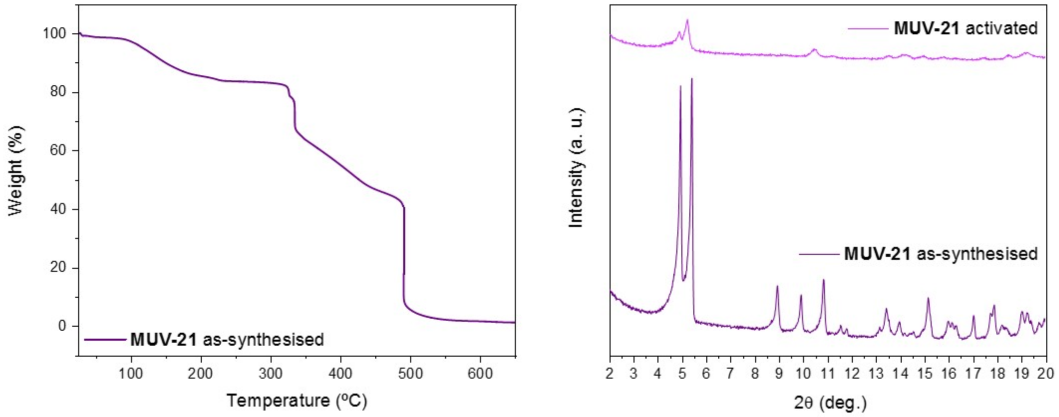

Figure S38. (left) TGA of **MUV-21**; (right) PXRD patterns of as-synthesised **MUV-21** and after activation (1 hour heating at 70 ºC).

6. Gas adsorption

High-pressure gravimetric CO_2 adsorption isotherms were measured at different temperatures, ranging from 283 to 318 K, in an IGA-100 gas sorption analyser (from Hiden Isochema) using approximately 50 mg of sample. Before each adsorption experiment, the sample was outgassed at 393 K under vacuum (10^{-5} Pa) for two hours. Equilibrium conditions corresponded to 600 s interval, and 0.001 mg min^{-1} tolerance.

Table S9. Sorption capacity at 6 bar and different temperatures of **MUV-20a** and **MUV-20b**.

Temperature (ºC)	mmol CO_2/ g		Sorption capacity (%)	
	**MUV-20a**	**MUV-20b**	**MUV-20a**	**MUV-20b**
10	1.91	1.71	8.41	7.52
25	1.66	1.47	7.33	6.47
40	1.45	1.31	6.38	5.79
55	1.23	1.15	5.42	5.08

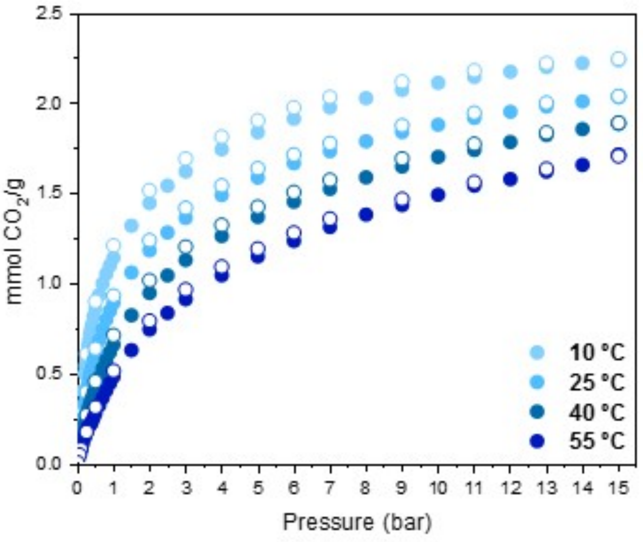

Figure S39. CO_2 isotherms at different temperatures for **MUV-20a**.

Table S10. Sorption capacity at 15 bar and different temperatures of **MUV-20a**.

Temperature (ºC)	mmol CO_2/ g	Sorption capacity (%)
10	2.25	9.85
25	2.03	8.94
40	1.88	8.30
55	1.71	7.52

The experimental isotherms were fitted by using a fourth grade virial equation[4]:

$$\ln\left(\frac{P}{Q}\right) = A_0 + A_1 Q + A_2 Q^2 + A_3 Q^3 + A_4 Q^4$$

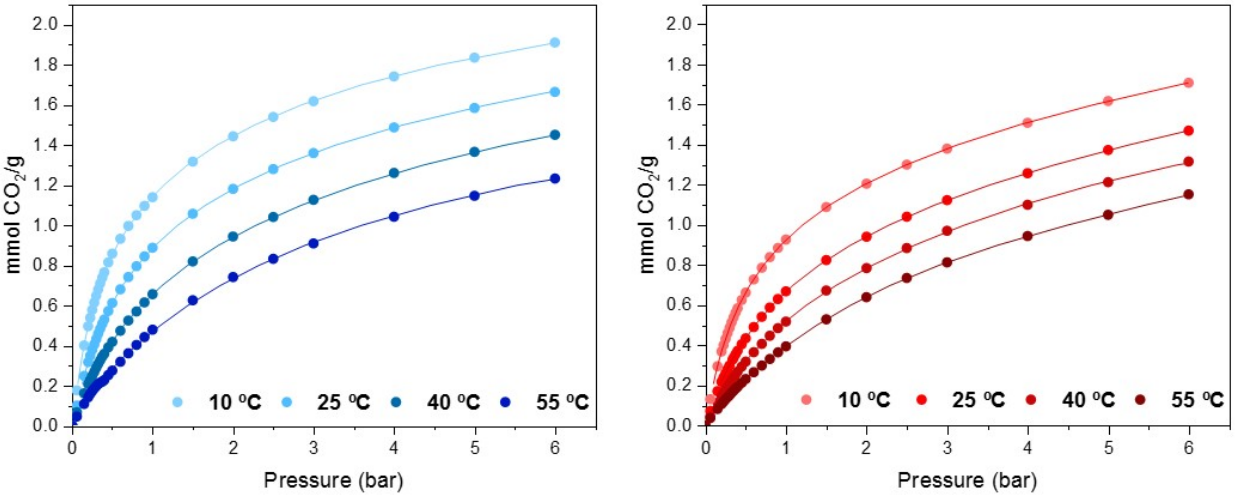

Figure S40. Virial fitting of CO_2 isotherms for **MUV-20a** (left) and **MUV-20b** (right).

The heat of adsorpion was calculated according to the Clausius-Clapeyron equation[5] through the data extracted from the experimental isotherms at different termperatures:

$$q_{st} = R \cdot T^2 \cdot \left[\frac{\partial(\ln P)}{\partial T}\right]_{Q=cte} = -R \cdot \left[\frac{\partial(\ln P)}{\partial\left(1/T\right)}\right]_{Q=cte}$$

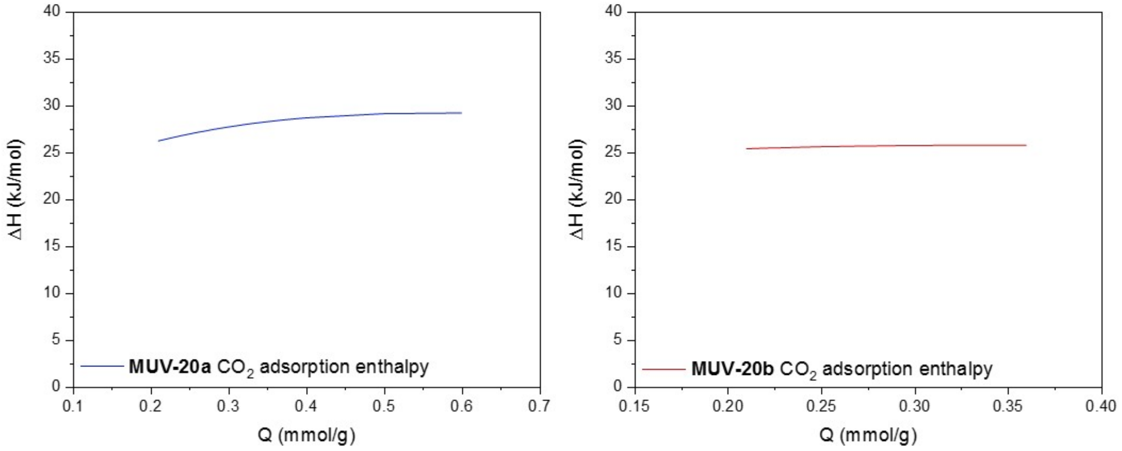

Figure S41. Isosteric heat of adsorption of CO_2 on **MUV-20a** (left) and **MUV-20b** (right), according to the Clausius-Clapeyron equation.

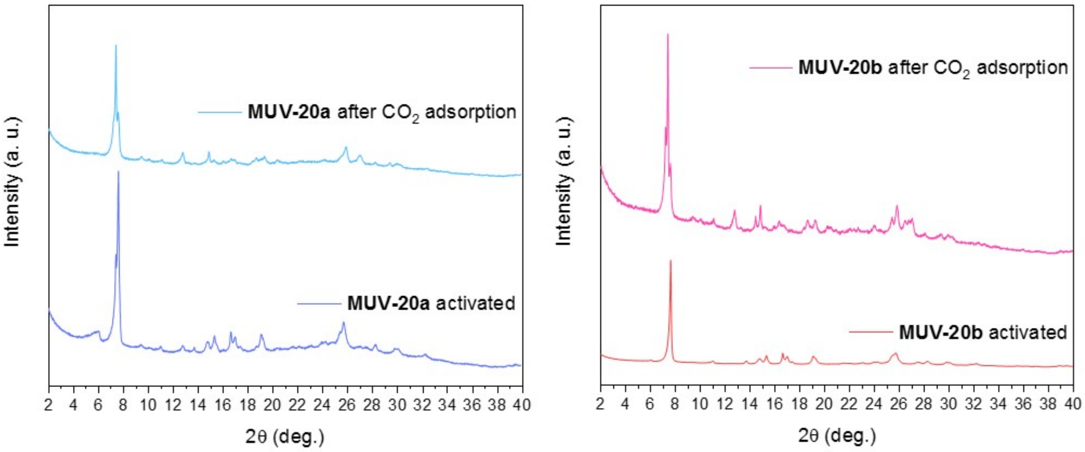

Figure S42. PXRD of **MUV-20** crystals before and after CO_2 adsorption.

7. Scanning electron microscopy (SEM)

Images from Scanning Electron Microscope were taken with an Hitachi S-4800.

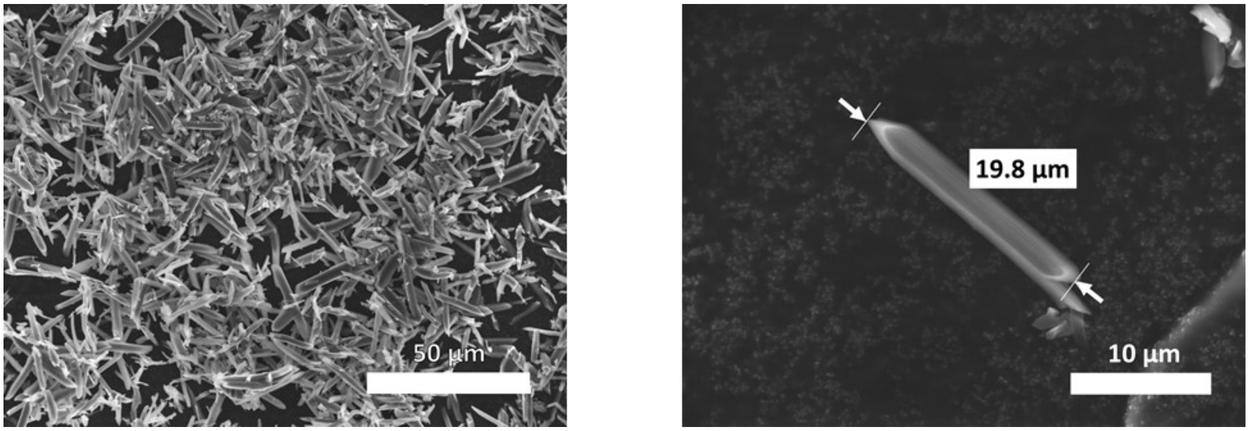

Figure S43. Scanning electron microscopy images from **MUV-20a** showing the size around 20 µm**.**

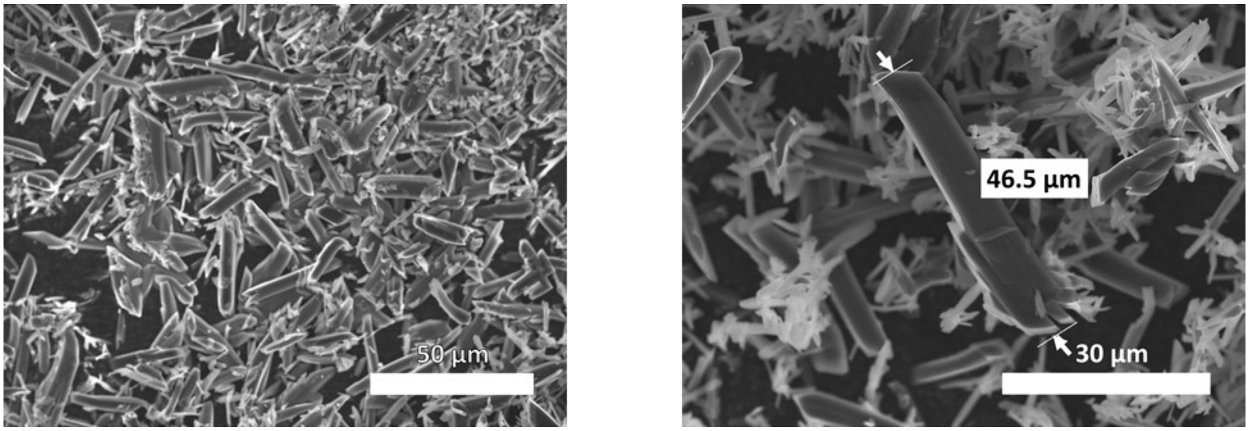

Figure S44. Scanning electron microscopy images from **MUV-20b** showing the size around 46 µm**.**

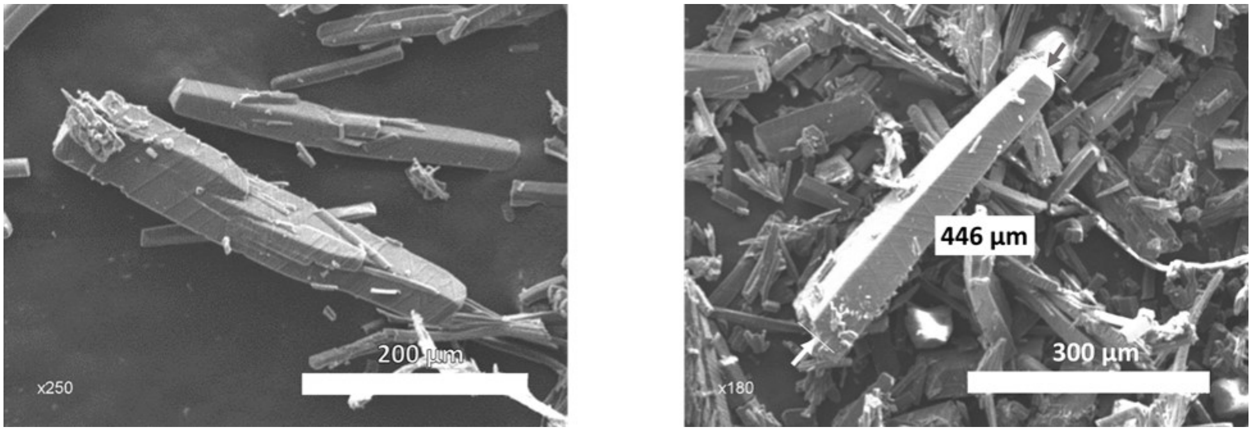

Figure S45. Scanning electron microscopy images from **MUV-21** showing the size around 450 µm.

8. Electrical conductivity

8.1. 2-probe measurements

Electrical conductivity was calculated from the following equation:

$\sigma = (1/R) \times (l/wt)$,

in which σ is conductivity (S cm^{-1}), l is the longitude, w the width, and t the thickness (cm) of the pellet/crystal, and R is the resistance (Ω).

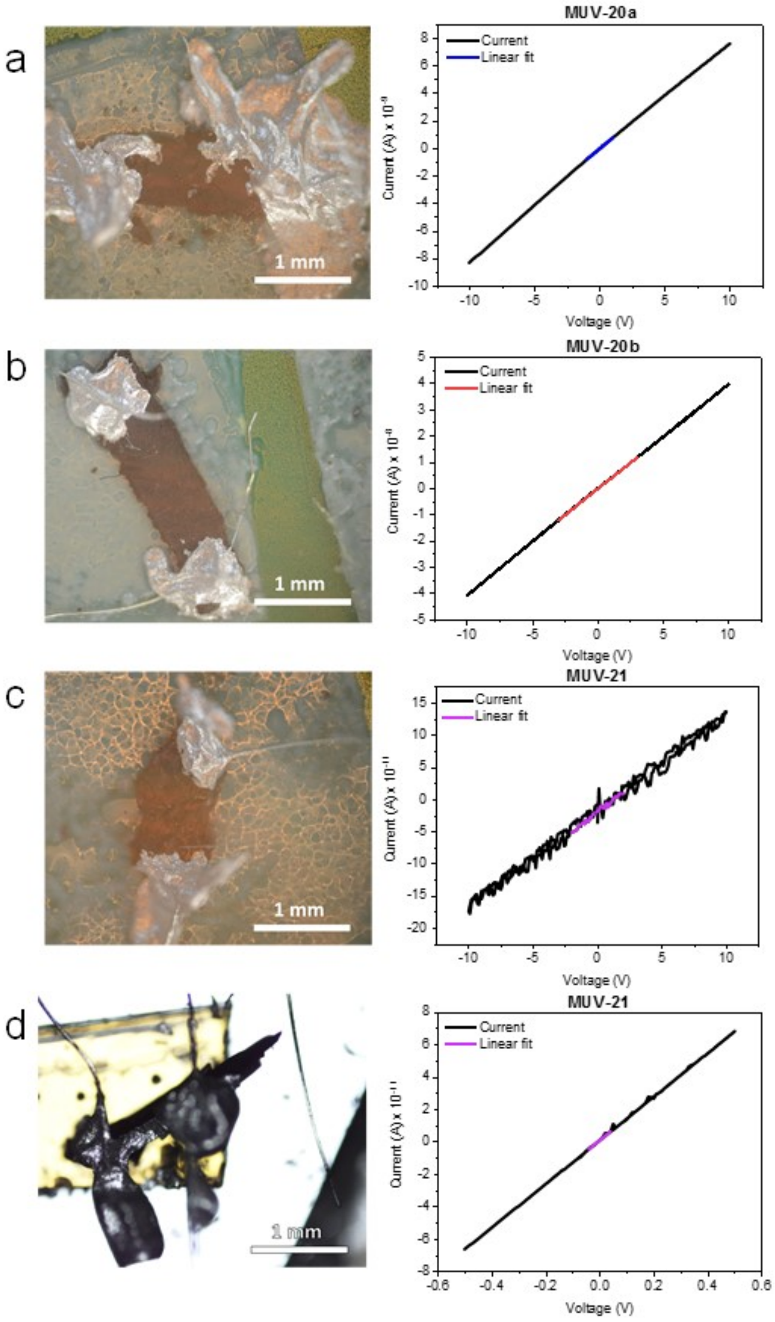

Figure S46. Electrical conductivity measurements of a) **MUV-20a** (pellet), b) **MUV-20b** (pellet), c) **MUV-21** (pellet), d) **MUV-21** (single crystal): (left) optical microscope images of the measured samples; (right) representation of the current vs voltage.

Table S11. Electrical conductivity measurements of **MUV-20a**, **MUV-20b**, and **MUV-21** from a 2-probe pellet, and **MUV-21** from a single crystal.

	Width (cm)	Length (cm)	Thickness (cm)	R (Ω) at 300 K	σ (S cm^{-1}) at 300 K
**MUV-20a**	66.2×10^{-3}	21.2×10^{-2}	4.2×10^{-3}	1.25×10^{9}	6.07×10^{-7}
**MUV-20b**	68.2×10^{-3}	17.7×10^{-2}	7.7×10^{-3}	2.49×10^{8}	1.35×10^{-6}
**MUV-21**[a]	66.5×10^{-3}	12.0×10^{-2}	4.6×10^{-3}	6.28×10^{10}	6.23×10^{-9}
**MUV-21**[b]	12.1×10^{-2}	67.4×10^{-2}	9.3×10^{-3}	2.42×10^{8}	7.95×10^{-8}

[a]2-Probe pellet; [b]2-probe single crystal

Table S12. Conductivity values at room temperature of different MOFs, COFs and HOFs containing the TTFTB ligand

	Material	σ (S·cm^{-1})	σ method	reference
**MOFs**	**Cd_2(TTFTB)**	$2.9(5) \times 10^{-4}$	2-probe single crystal	*J. Am. Chem. Soc.* **2015**, *137*, 1774–1777
		$2.5(5) \times 10^{-4}$	2-probe single crystal	*J. Am. Chem. Soc.* **2016**, *138*, 14772–14782
		1.91×10^{-4}	4-probe single crystal	*J. Am. Chem. Soc.* **2016**, *138*, 14772–14782
		6.8×10^{-4}	4-probe single crystal	*J. Am. Chem. Soc.* **2015**, *137*, 1774–1777
		4.39×10^{-6}	4-probe single pellet	*J. Am. Chem. Soc.* **2016**, *138*, 14772–14782
		2.7×10^{-6}	vdP[a] pellet	*J. Am. Chem. Soc.* **2016**, *138*, 14772–14782
		$2.1(1) \times 10^{-6}$	2-probe single pellet	*J. Am. Chem. Soc.* **2016**, *138*, 14772–14782
		$2.4(7) \times 10^{-7}$	2-probe single crystal	*J. Am. Chem. Soc.* **2016**, *138*, 14772–14782
	**Co_2(TTFTB)**	1.53×10^{-5}	2-probe single crystal	*J. Am. Chem. Soc.* **2015**, *137*, 1774–1777
		5×10^{-5}	4-probe single crystal	*J. Am. Chem. Soc.* **2015**, *137*, 1774–1777
	**Mn_2(TTFTB)**	9.0×10^{-5}	2-probe single crystal	*J. Am. Chem. Soc.* **2015**, *137*, 1774–1777
		1×10^{-4}	4-probe single crystal	*J. Am. Chem. Soc.* **2015**, *137*, 1774–1777
	**Zn_2(TTFTB)**	5.0×10^{-4}	2-probe single crystal	*J. Am. Chem. Soc.* **2015**, *137*, 1774–1777
		5.0×10^{-4}	Time-Resolved Terahertz Spectroscopy	*J. Am. Chem. Soc.* **2019**, *141*, 9793–9797
	**Zn_2(TTFTB)(H_2O)$_2$**	2.5×10^{-10}	EIS[b]	*Polyhedron* **2018**, *154,* 334–342
	**Zn_2(TTFTB)(H_2O)$_2$@I_2**	1.6×10^{-9}	EIS[b]	*Polyhedron* **2018**, *154,* 334–342
	**La_4(TTFTB)$_4$**	$2.5(7) \times 10^{-6}$	2-probe pellet	*Chem. Sci.* **2019**, *10*, 8558–8565
	**La(TTFTB)**	$9.0(4) \times 10^{-7}$	2-probe pellet	*Chem. Sci.* **2019**, *10*, 8558–8565
	**La_4(TTFTB)$_3$**	$1.0(5) \times 10^{-9}$	2-probe pellet	*Chem. Sci.* **2019**, *10*, 8558–8565
	**MUV-5(Gd)**	2.0×10^{-7}	4-probe pellet	*Chem. Eur. J.* **2019**, *25*, 12636–12643
	**MUV-5(Tb)**	1.5×10^{-7}	4-probe pellet	*Chem. Eur. J.* **2019**, *25*, 12636–12643

	**MUV-5a(Dy)**	3.9×10^{-7}	4-probe pellet	*Chem. Eur. J.* **2019**, *25*, 12636–12643
	**MUV-5a(Ho)**	6.7×10^{-6}	4-probe pellet	*Chem. Eur. J.* **2019**, *25*, 12636–12643
	**MUV-5a(Er)**	7.4×10^{-6}	4-probe pellet	*Chem. Eur. J.* **2019**, *25*, 12636–12643
	**MUV-5b(Dy)**	3.3×10^{-7}	4-probe pellet	*Chem. Eur. J.* **2019**, *25*, 12636–12643
	**$Er_4(TTFTB)_3$**	1×10^{-9}	2-probe pellet	*Inorg. Chem.* **2019**, *58*, 3698–3706
	**$Er_4(TTFTB_{)3}(I_3)_2$**	2×10^{-8}	2-probe pellet	*Inorg. Chem.* **2019**, *58*, 3698–3706
	**$Tb_4(TTFTB_{)3}(I_3)_2$**	4×10^{-8}	2-probe pellet	*Inorg. Chem.* **2019**, *58*, 3698–3706
	**$Tb_4(TTFTB)_3$**	1×10^{-8}	2-probe pellet	*Inorg. Chem.* **2019**, *58*, 3698–3706
	**$Dy_4(TTFTB)_3(I_3)_2$**	1×10^{-8}	2-probe pellet	*Inorg. Chem.* **2019**, *58*, 3698–3706
	**$Dy_4(TTFTB)_3$**	7×10^{-9}	2-probe pellet	*Inorg. Chem.* **2019**, *58*, 3698–3706
	**$Ho_4(TTFTB)_3(I_3)_2$**	8×10^{-9}	2-probe pellet	*Inorg. Chem.* **2019**, *58*, 3698–3706
	**$Ho_4(TTFTB)_3$**	1×10^{-9}	2-probe pellet	*Inorg. Chem.* **2019**, *58*, 3698–3706
	**$Yb_6(TTFTB)_5$**	$9(7) \times 10^{-7}$	2-probe pellet	*Isr. J. Chem*. **2018**, *58*, 1119–1122
	**$Lu_6(TTFTB)_5$**	$3(2) \times 10^{-7}$	2-probe pellet	*Isr. J. Chem*. **2018**, *58*, 1119–1122
	**MUV-2**	3.7×10^{-11}	2-probe pellet	*Beilstein J. Nanotechnol*. **2019**, *10*, 1883–1893
	**C_{60}@MUV-2**	4.7×10^{-9}	2-probe pellet	*Beilstein J. Nanotechnol*. **2019**, *10*, 1883–1893
**COFs**	**TTF-COF**	1.2×10^{-6}	2-probe film	*Chem. Sci.* **2014**, *5*, 4693–4700
	**TTF-COF@I_2**	2.8×10^{-3}	2-probe film	*Chem. Sci.* **2014**, *5*, 4693–4700
	**JUC-518@I_2**	2.9×10^{-7}	2-probe pellet	*J. Am. Chem. Soc.* **2019**, *141*, 13324–13329
	**JUC-519@I_2**	1.8×10^{-7}	2-probe pellet	*J. Am. Chem. Soc.* **2019**, *141*, 13324–13329
	**TTF-DMTA**	1.8×10^{-4}	2-probe pellet	*ACS Appl. Mater. Inter.* **2020**, *12*, 19054–19061
**HOFs**	**HOF-110 (activated)**[c]	7.8×10^{-10}	2-probe pellet	*ACS Materials Lett.* **2022**, *4*, 128−135
	**HOF-110**[c]	2.2×10^{-8}	2-probe pellet	*ACS Materials Lett.* **2022**, *4*, 128−135
	**HOF-110@I_2-1**[c]	2.7×10^{-7}	2-probe pellet	*ACS Materials Lett.* **2022**, *4*, 128−135
	**HOF-110@I_2-2**[c]	6.0×10^{-7}	2-probe pellet	*ACS Materials Lett.* **2022**, *4*, 128−135
	**MUV-20a**	6.07×10^{-7}	2-probe pellet	This work
	**MUV-20b**	1.35×10^{-6}	2-probe pellet	This work
	**MUV-21**	6.23×10^{-9}	2-probe pellet	This work
		7.95×10^{-8}	2-probe single crystal	This work

[a] Van der Pauw method.[b] Electrochemical Impedance Spectroscopy.[c] HOF-110 is not based on TTFTB, but has been included in the list for completion, as it is the only conductive HOF reported to date.

8.2. 4-probe measurements

In order to compare different types of conductivity measurements, both 2-probe and 4-probe methodologies were measured on the same pellets. The obtained values are within the same range for **MUV-20a** and **MUV-20b**, with slightly lower resistance (i.e., higher conductivities) using the 4-probe technique (see Figure S47). For **MUV-21**, it was not possible to obtain any reliable data using the 4-probe as it is very insulating and the Keithley 2450 used cannot inject current.

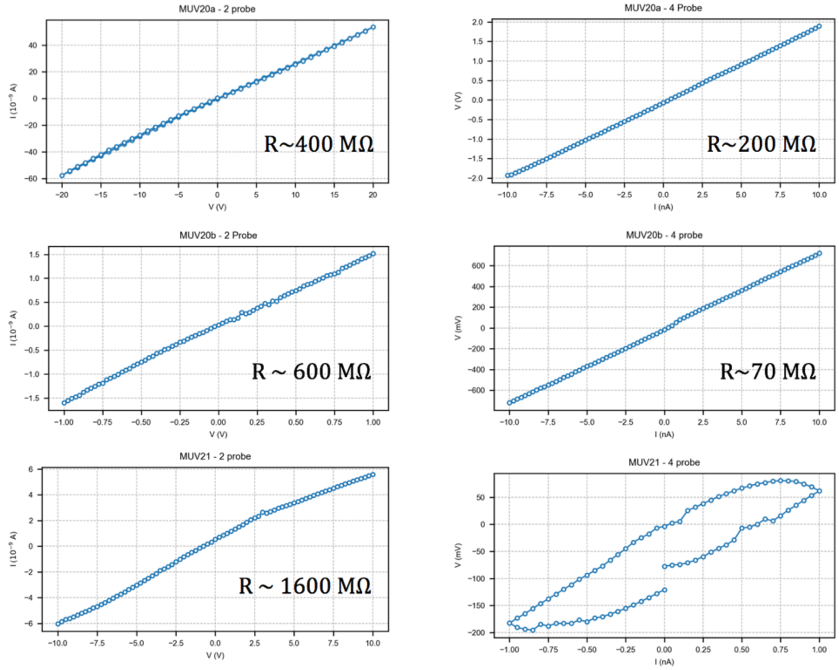

Figure S47. I-V measurements for **MUV-20a**, **MUV-20b** and **MUV-21** using both 2-probe and 4-probe methodologies on the same pellets. The resistivity values, R, indicated for each measurement, show values that are within the same range regardless of the methodology used. Note that for **MUV-21**, it was not possible to obtain any reliable data using the 4-probe technique, because the sample is very insulating and the Keithley 2450 used cannot inject current in such an isolating system.

9. Theoretical Calculations

Quantum chemical calculations in periodic boundary conditions were carried out within the density functional theory (DFT) framework as implemented in the all-electron full-potential FHI-AIMS electronic structure code package.[6,7,8] Minimum-energy geometries for **MUV-20a**, **MUV-20b**, and **MUV-21** were obtained, starting from the experimental crystal structures, after full lattice and ionic relaxation using the GGA-type PBEsol functional[9] and the numeric atom-centered orbital Tier-1 basis set.[6] Solvent molecules present in the pores were excluded in the calculations with the exception of the relevant DMA^+ countercations in **MUV-21**. Dispersion forces were treated by means of the vdW Hirshfeld correction based on the partitioning of the electron density as described by Tkatchenko and Scheffler.[10] Lattice parameters of the optimized HOFs are summarized in Table S10. The minimum-energy crystal structure obtained for **MUV-21** with the vdW Hirshfeld correction showed a slightly short "*a*" lattice parameter (8.55 Å) compared to the experimental X-ray data (8.93 Å). This suggests that either the solvent molecules within the big pore of the HOF not considered in the calculations are important to describe the final crystal structure of **MUV-21**, or the vdW correction overestimates the π–π interaction between the TTFTB ligands. By switching off the vdW correction, a better match of the "*a*" lattice parameter (8.86 Å) with respect to the experiment was found. However, we decided to use the vdW correction by default in all calculations for consistency. The electronic band structure and density of states were obtained by means of the hybrid HSE06 functional. A full *k*-path in the $P\bar{1}$ first Brillouin zone of Γ−Z−Y−X−V−U−T−R−Γ and a 3×3×3 k-grid were employed. The size of the polaron generated in both frameworks was assessed by extracting a large (11 units) 1D TTF stacking from each optimized crystal structure, and replacing the TTF unit in the middle of the stack by an oxidized TTF. Environment effects were included by means of a dielectric continuum according to the PCM scheme ($\varepsilon = 7.43$ corresponding to THF) as implemented in the Gaussian-16.A03 suite of programs.[11] The spin density was calculated on these oligomers using the HSE06 functional and the 6-31G(d,p) basis set,[12] and the isovalue contours were set to a value of 0.001 atomic units (au).

In hopping-like models for charge transport, the rate constant of the charge transfer between neighboring molecular moieties can be described by means of the Markus equation,[13]

$$k = \frac{2\pi}{\hbar}\frac{J^2}{\sqrt{4\pi\lambda k_b T}} e^{-\frac{(\lambda+\Delta G^\circ)^2}{4\lambda k_b T}}$$

$$\lambda = \lambda_1 + \lambda_2$$

$$\lambda_1 = E_N^{gC} - E_N^{gN}$$

$$\lambda_2 = E_C^{gN} - E_C^{gC}$$

where J is the electronic coupling, λ is the total reorganization energy, and ΔG° is the total change of the free energy for the electronic transfer reaction. In our case, the total reorganization energy λ is composed of λ_1, the reorganization energy for the neutral TTFTB, and λ_2, the reorganization energy for the cation form of the ligand. E_N^{gN} is the energy of the neutral TTFTB at its neutral geometry, E_N^{gC} is the energy of the neutral form at the cation geometry, E_C^{gN} is the energy of the

cation at the neutral geometry, and E_C^{gC} is the energy of the cation at the cation geometry. Note that since the electronic couplings, in this case, are calculated for the different dimers of the same molecular system (TTFTB), we neglect $\Delta G°$.

The electronic couplings of the different TTF dimers were computed under the fragment-orbital FO-DFT framework as implemented in FHI-AIMS, by means of the PBE/Tier-1 level of theory.[14] Among the different flavors available, the H_{2n-1}@DA scheme was used, where neutral fragment calculations are combined for hole transfer with a reset of the occupation number in the highest occupied molecular orbital. The reorganization energy of the TTFTB moiety was computed by means of molecular calculations for the fully-protonated system at the DFT level using Gaussian-16.A03.[11] The HSE06 functional and the double-polarized Pople's 6-31G(d,p) basis set were employed. Four different calculations were carried out for computing λ: the neutral and the oxidized states at both the minimum-energy geometry of the neutral and the oxidized ligand. The values obtained for the electronic couplings and the electron-transfer rate constants after applying the Marcus equation are summarized in Table S12.

The crystalline geometries, spin densities, and orbitals were displayed using the software VESTA.[15]

Table S13. Lattice parameters calculated for the minimum-energy crystal structures optimized at the PBEsol level in comparison with the experimentally resolved crystal structures for **MUV-20a**, **MUV-20b**, and **MUV-21**.

System	*a* (Å)	*b* (Å)	*c* (Å)	*α* (°)	*β* (°)	*γ* (°)
**MUV-20a**[a]	9.84	11.39	15.96	92.6	108.0	102.4
**MUV-20a** (experimental)	10.06	11.59	16.00	92.5	107.7	102.4
**MUV-20b**[a]	10.23	12.43	14.92	81.7	71.3	66.1
**MUV-20b** (experimental)	10.34	12.37	15.22	86.2	70.6	66.2
**MUV-21**[b]	8.55	18.22	19.39	114.5	93.4	95.4
**MUV-21**[c]	8.86	18.21	19.47	114.5	94.7	94.9
**MUV-21** (experimental)	8.93	18.45	19.48	114.5	96.4	93.1

[a] Minimum-energy crystal structures considering the two TTFTBs of the unit cell singly deprotonated.
[b,c] Lattice parameters of **MUV-21** were obtained with (b) and without (c) vdW dispersion corrections, respectively.

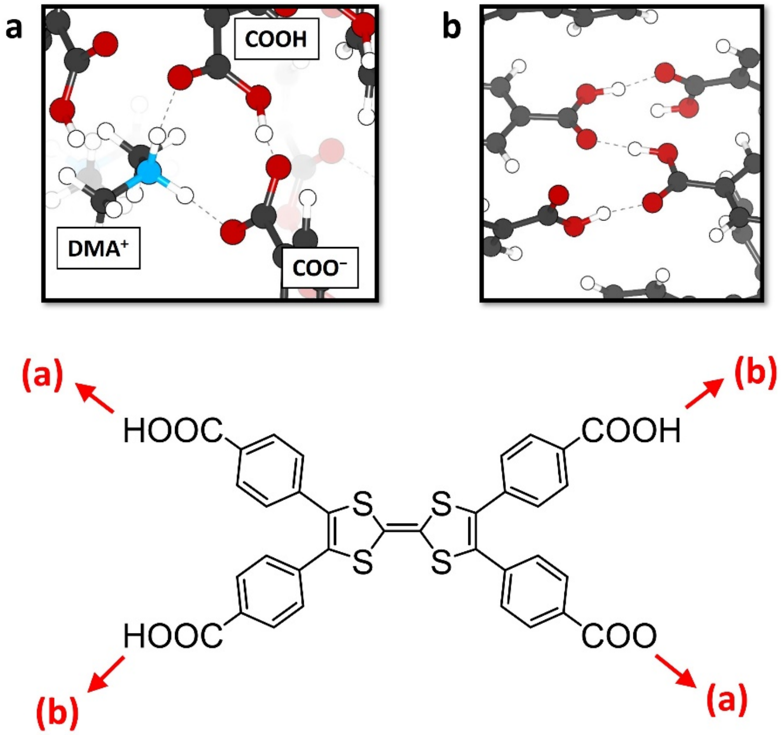

Figure S48. H-bonding connection in **MUV-21**: a) between two TTFTB units (one of them with a deprotonated carboxylic acid; COO^{-}) and the countercation in two diagonal carboxylic positions of the ligand, and b) between four TTFTBs for the other two diagonal positions.

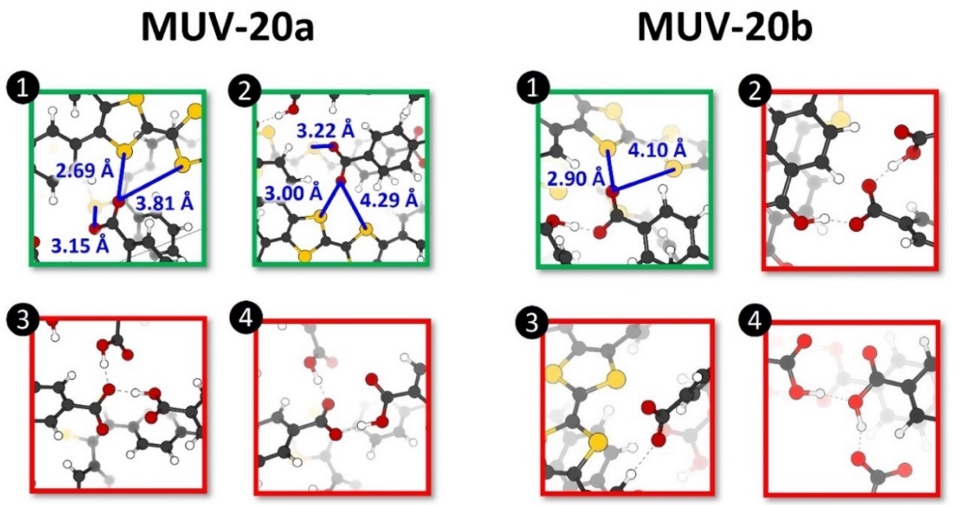

MUV-20a

Deprotonated position	Relative Energy (eV)	
	PBEsol	HSE06
1	0.00	0.01
2	0.02	0.00
3	0.30	0.35
4	0.39	0.32

MUV-20b

Deprotonated position	Relative Energy (eV)	
	PBEsol	HSE06
1	0.00	0.00
2	0.08	0.13
3	0.24	0.21
4	0.26	0.25

Figure S49. Minimum-energy crystal structures calculated for **MUV-20a** and **MUV-20b** placing the deprotonated carboxylate group in the four inequivalent carboxylic acid positions of the unit cell. Relative energies are indicated at two levels of theory (PBEsol and HSE06).

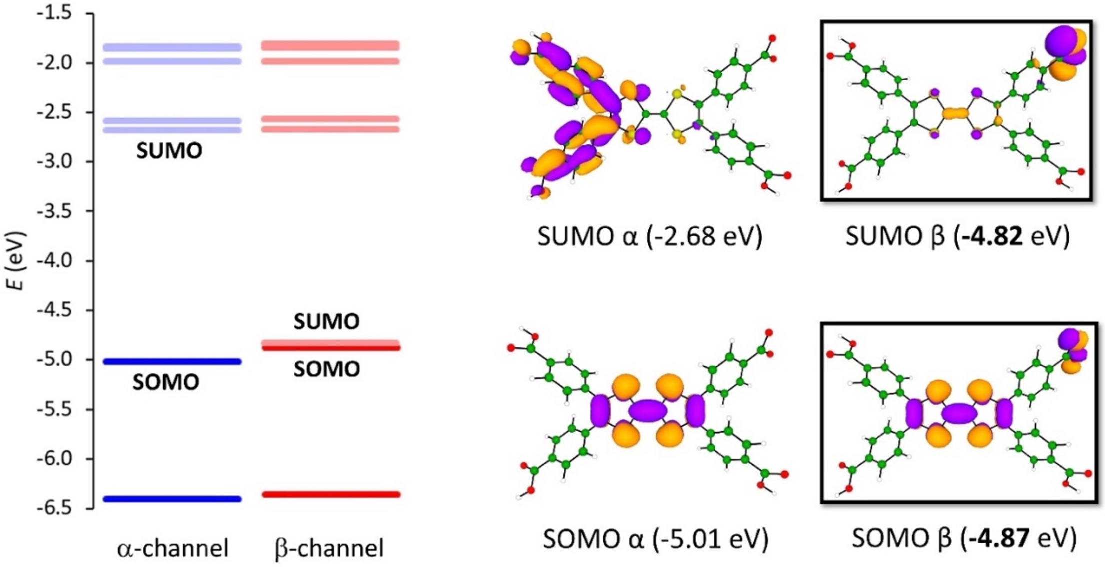

Figure S50. Frontier molecular orbital diagram and topologies for the α- and β-spin channels calculated for the singly deprotonated, neutral TTFTB ligand in gas phase at the HSE/6-31G(d,p) level.

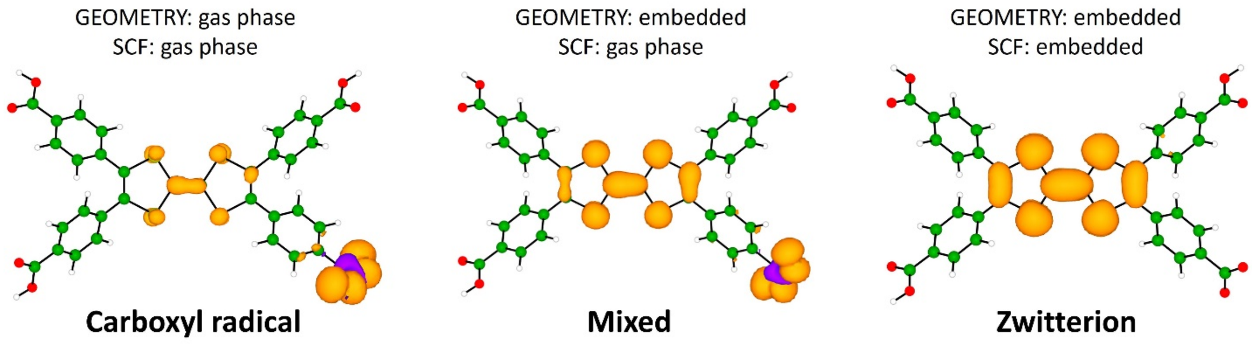

Figure S51. Spin density computed for the singly deprotonated, neutral TTFTB ligand at the HSE06/6-31G(d,p) level of theory in different situations of geometry and embedding conditions (polarizable continuum solvent model with $\varepsilon = 7.43$).

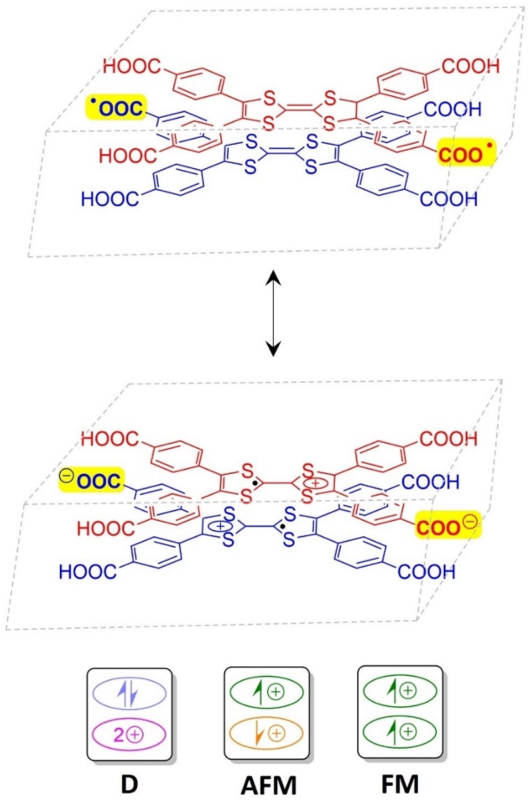

Figure S52. Schematic representation of spin configuration states in **MUV-20a** and **MUV-20b**, either in a carboxylic radical or a zwitterionic form: diamagnetic (D), antiferromagnetic (AFM), and ferromagnetic (FM).

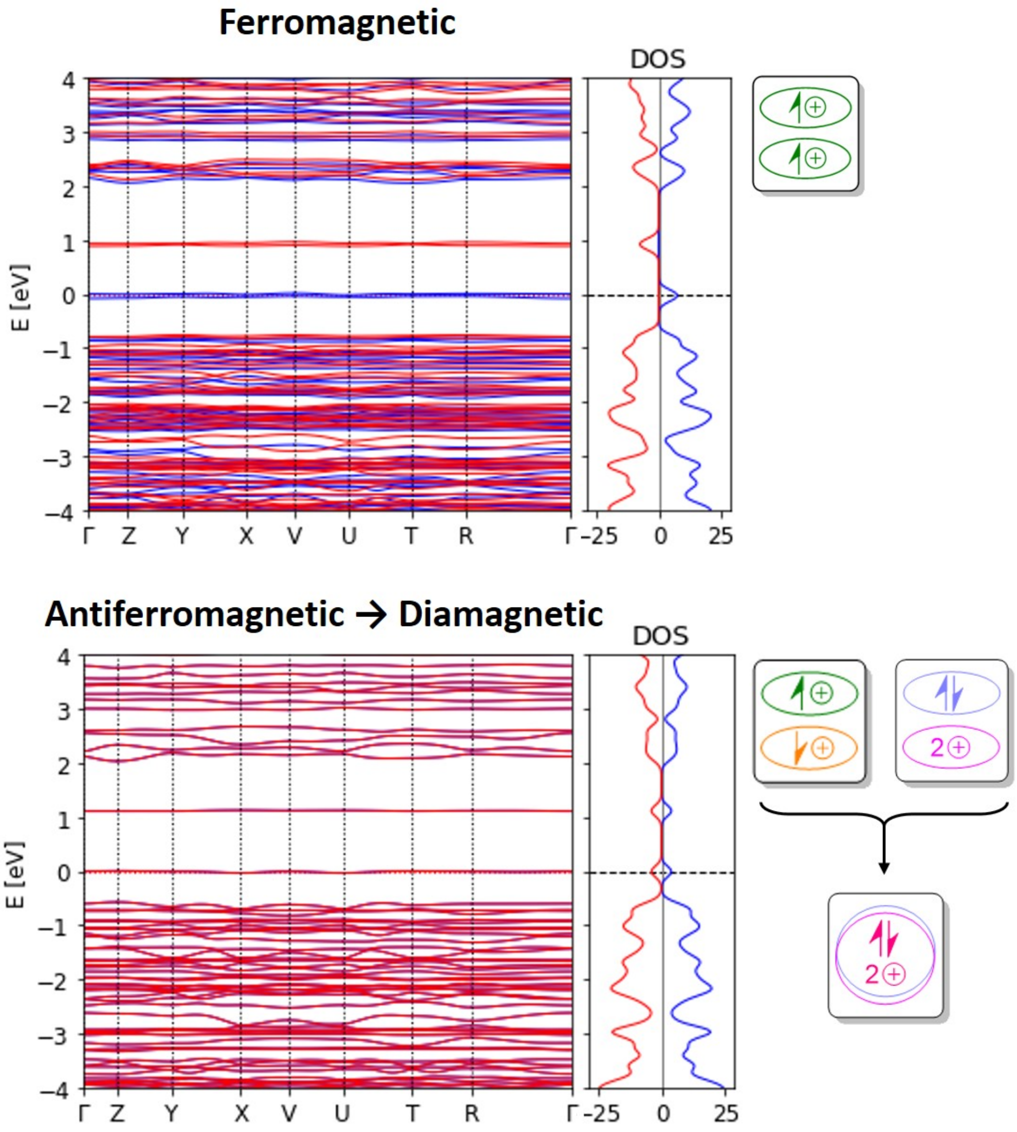

Figure S53. Band structure diagram and density of states (DOS) calculated at the HSE06 level for the ferromagnetic and antiferromagnetic spin configurations of **MUV-20a**. Spin-up α and spin-down β channels are displayed in blue and red, respectively. The Fermi level was set to the valence-band maximum (VBM). Note that the antiferromagnetic configuration evolves to a diamagnetic electronic structure delocalized over the two TTF units of the unit cell, as confirmed by the spin density (see Figure S54 below). The ferromagnetic configuration is calculated 0.33 and 0.17 eV more stable than the antiferromagnetic ("diamagnetic") system in **MUV-20a** and **MUV-20b**, respectively.

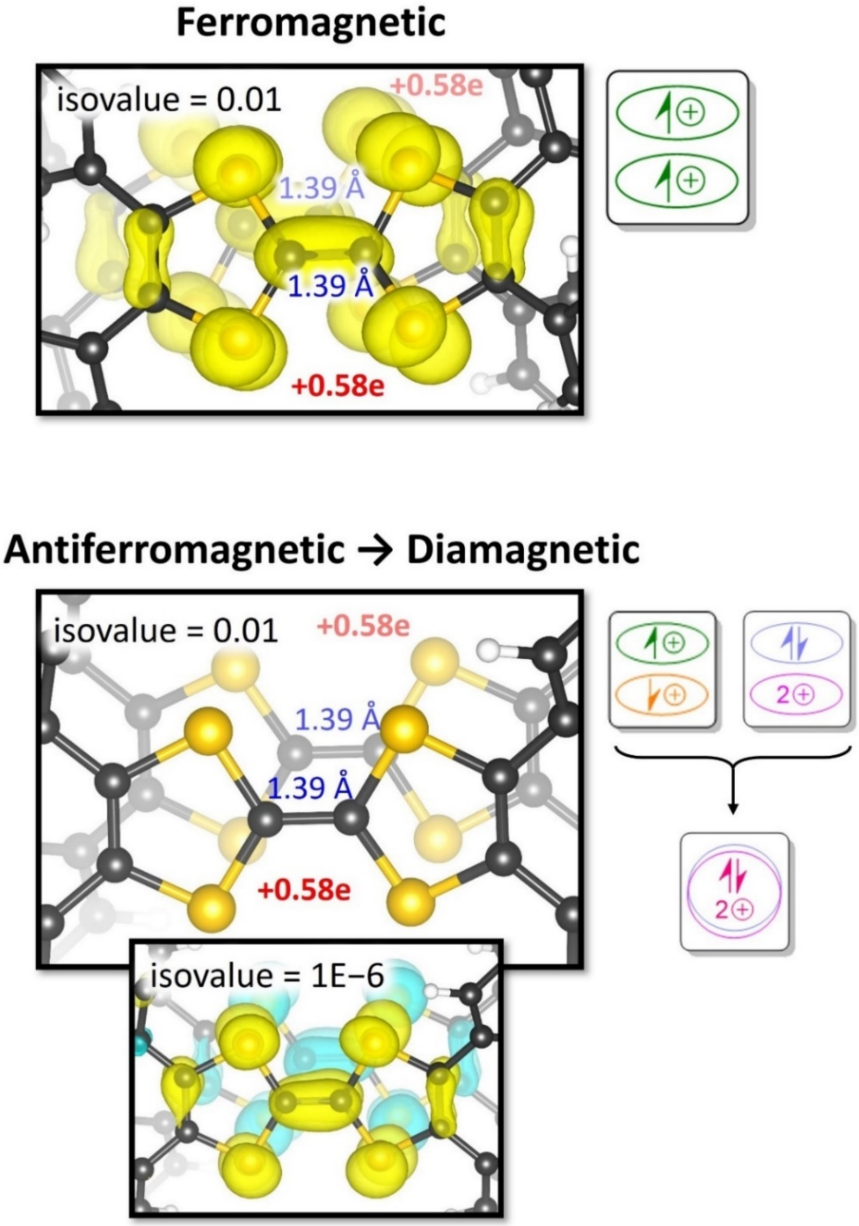

Figure S54. Spin density calculated at the HSE06 level for **MUV-20a** in a ferromagnetic (top) and antiferromagnetic (bottom) configuration. The initial antiferromagnetic electronic structure evolves towards a delocalized diamagnetic configuration as confirmed by the very small spin density on each TTF unit. Characteristic C–C distance (in blue) and charge accumulation increase (Δq, in red) of the TTF core are indicated. Unpaired-electron spin density on each TTF is calculated to be 0.90 and 7×10^{-5} for ferromagnetic and antiferromagnetic configurations, respectively.

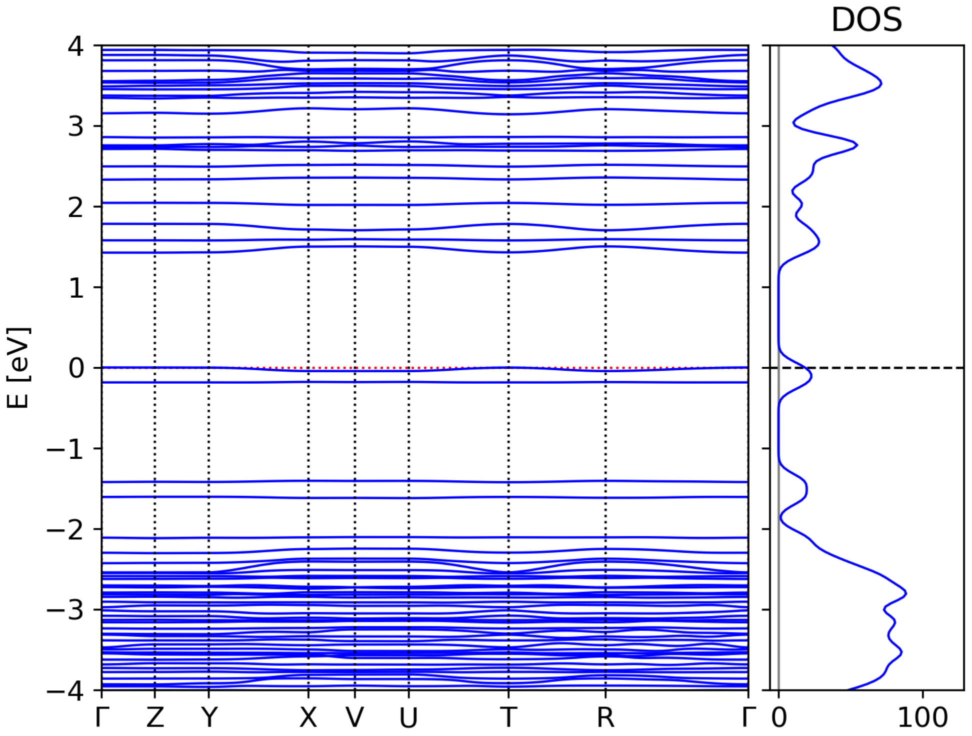

Figure S55. Band structure diagram and DOS calculated at the HSE06 level for **MUV-21**. The Fermi level was set to the VBM.

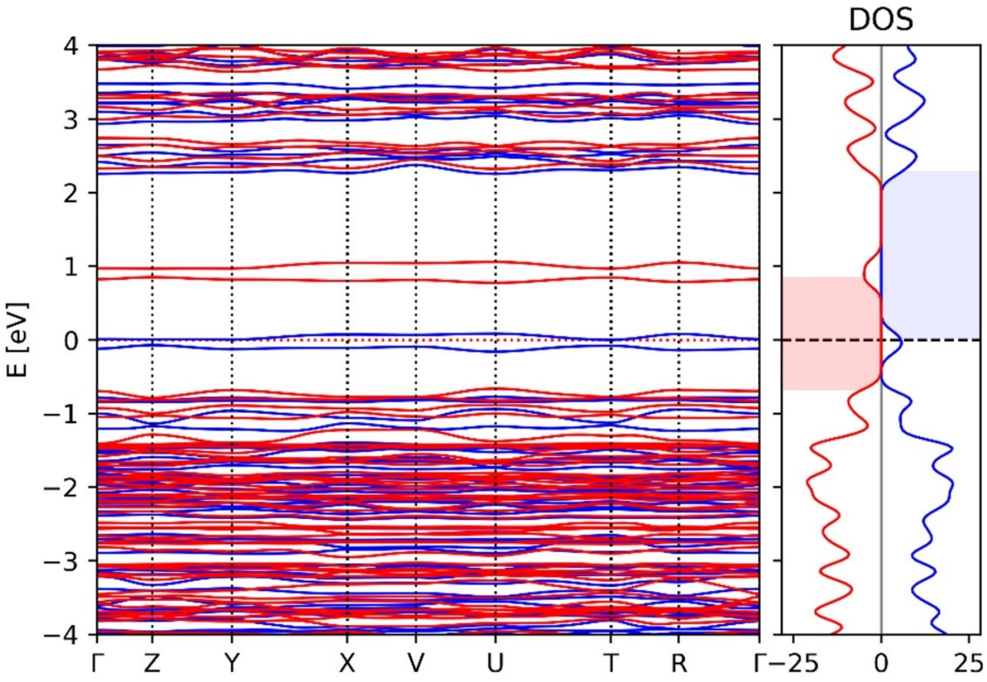

Figure S56. Band structure diagram and DOS calculated at the HSE06 level for ferromagnetic **MUV-20b**. The Fermi level was set to the VBM.

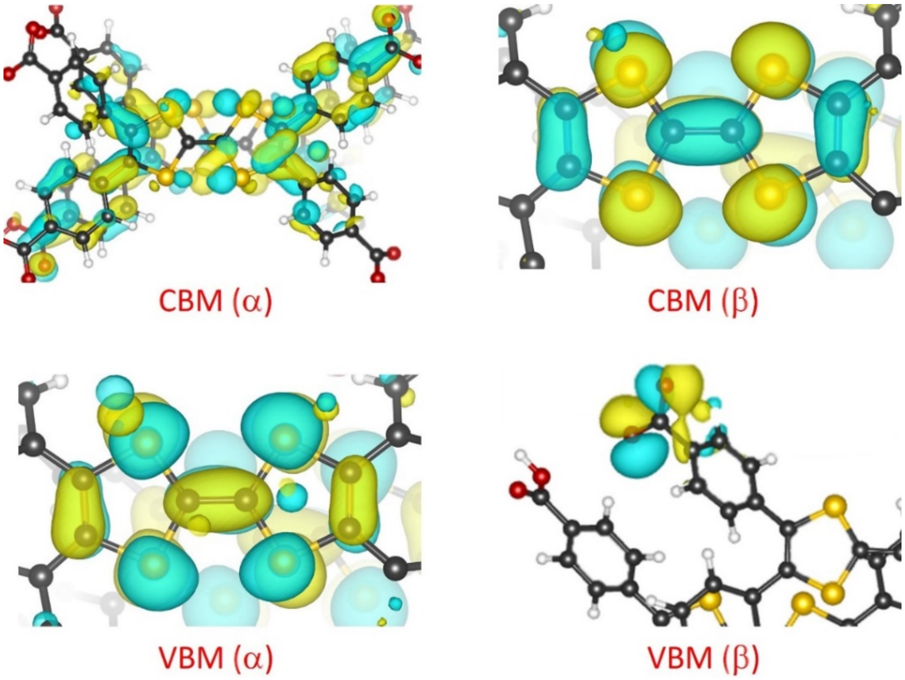

Figure S57. Valence-band maximum (VBM) and conduction-band minimum (CBM) in spin-up (α) and spin-down (β) channels calculated at the HSE06 level for ferromagnetic **MUV-20a**. Isovalue for the eigenstates is set to 0.06 au.

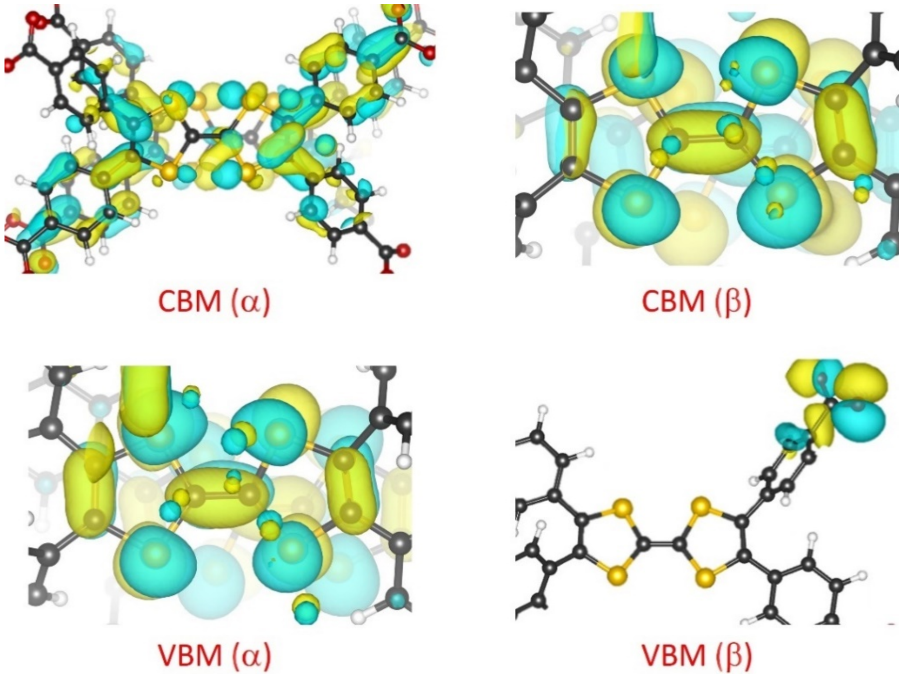

Figure S58. VBM and CBM in spin-up (α) and spin-down (β) channels calculated at the HSE06 level for ferromagnetic **MUV-20b**. Isovalue for the eigenstates is set to 0.06 au.

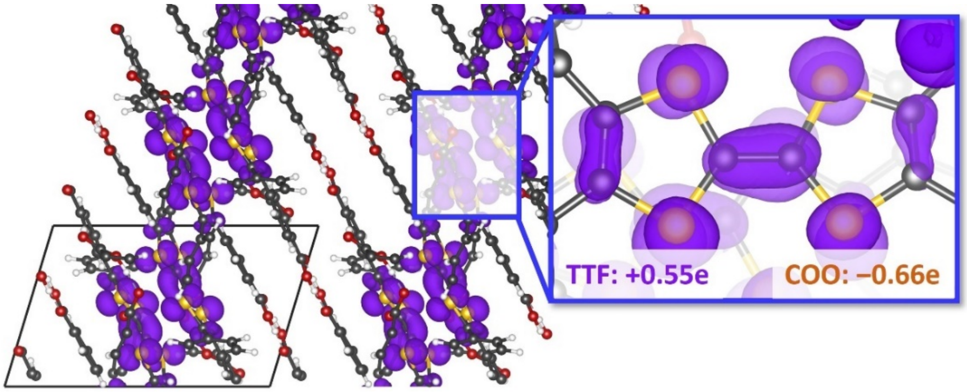

Figure S59. Unpaired-electron spin density computed at the HSE06 level for zwitterionic, ferromagnetic **MUV-20b** represented with isovalue contours of 0.008 au. The charge accumulation increase (Δq) for the TTF core and the carboxylate group with respect to isolated, fully protonated TTFTB ligand is indicated.

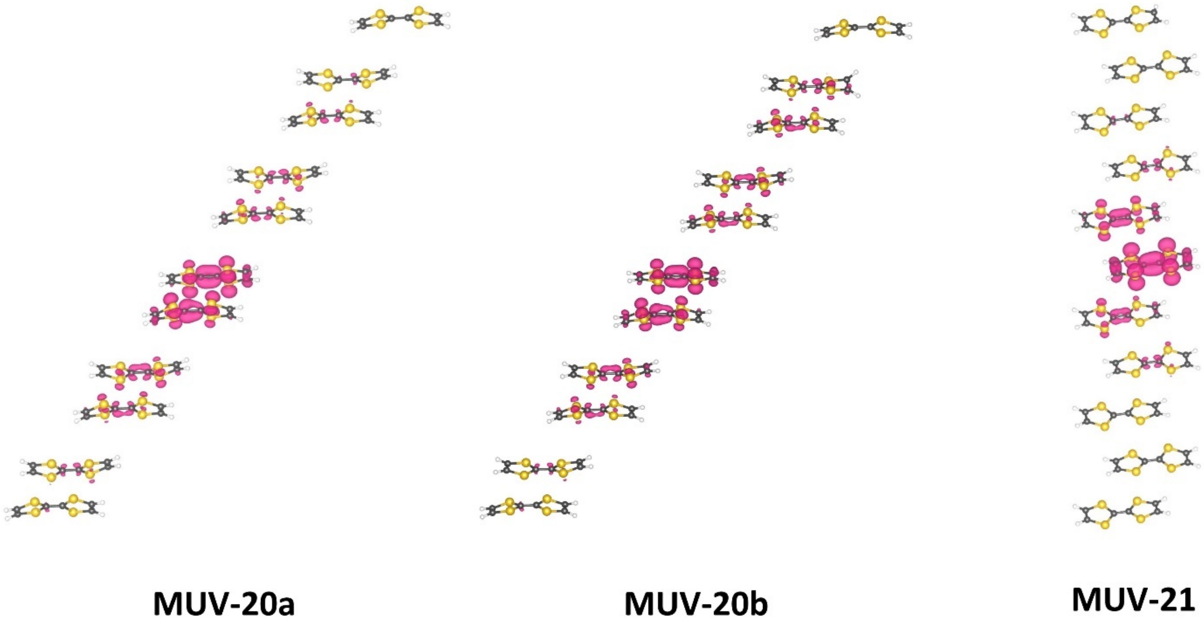

Figure S60. Spin density contours calculated for a 11-mer of TTF units extracted from the crystal structure of **MUV-20a**, **MUV-20b**, and **MUV-21**, and replacing the TTF in the middle of the stack by a fully-optimized oxidized $TTF^{\bullet+}$ unit. Isovalue countours were set to 0.001 au.

Table S14. Electronic couplings (*J*) and electron-transfer rate constants (*k*) calculated according to a hopping charge-transport regime (Marcus theory) for inequivalent TTFTB pairs in **MUV-20a**, **MUV-20b**, and **MUV-21**.

TTF···TTF dimers	*J* (meV)	*k* (s^{-1})[c]
**MUV-20a**[a]	152.05	9.09×10^{12}
**MUV-20a**[b]	14.24	7.98×10^{10}
**MUV-20b**[a]	91.64	3.30×10^{12}
**MUV-20b**[b]	37.41	5.51×10^{11}
**MUV-21**[a]	55.45	1.21×10^{12}
**MUV-21**[b]	7.18	2.03×10^{10}

[a,b] Dimer A and B, respectively, as depicted in Figure 7 of the main text.
[c] Total reorganization energy (λ) was calculated to be 0.43 eV for TTFTB.

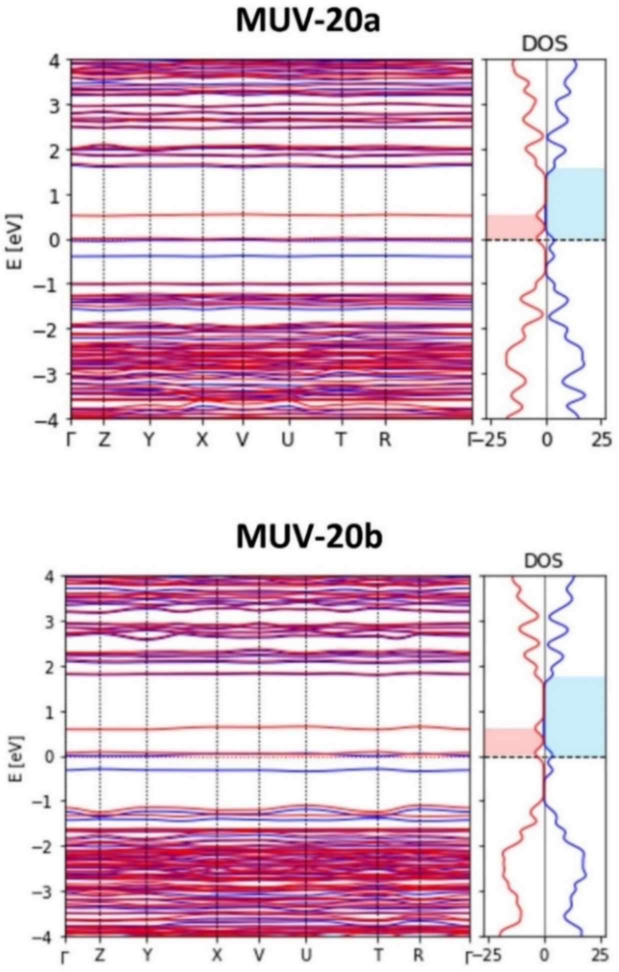

Figure S61. Electronic band structure diagram (left) and DOS (right) calculated for the zwitterionic half-protonated **MUV-20a** and **MUV-20b** at the HSE06 level. The Fermi level is set to the VBM. Spin-up α and spin-down β channels are displayed in blue and red, respectively. The bandgaps of α (1.68 and 1.80 eV, respectively) and β (0.51 and 0.52 eV, respectively) are colored in blue and red, respectively.

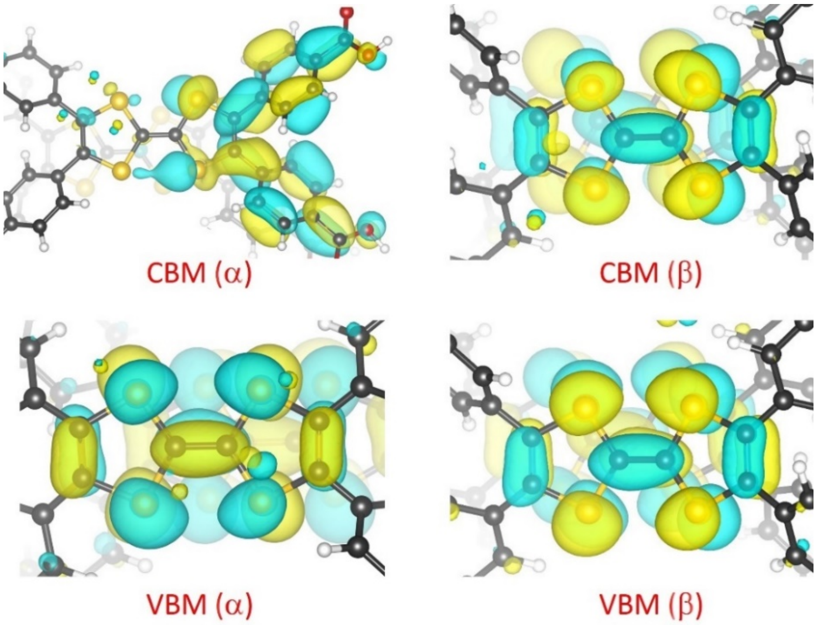

Figure S62. VBM and CBM computed at the HSE06 level for the half-protonated (50% fully protonated TTFTBs in the unit cell) **MUV-20a**. Isovalue contours for the eigenstates are set to 0.06 au.

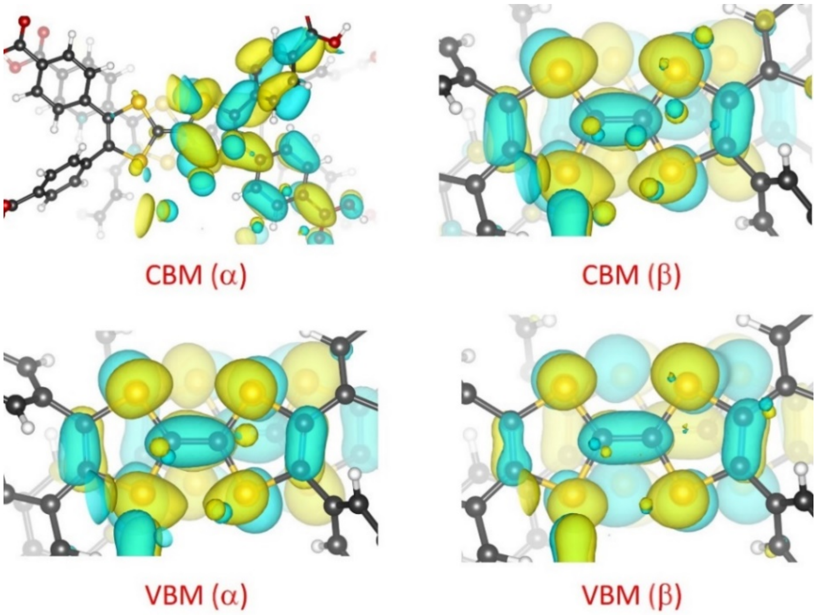

Figure S63. VBM and CBM computed at the HSE06 level for the half-protonated (50% fully protonated TTFTBs in the unit cell) **MUV-20b**. Isovalue contours for the eigenstates are set to 0.06 au.

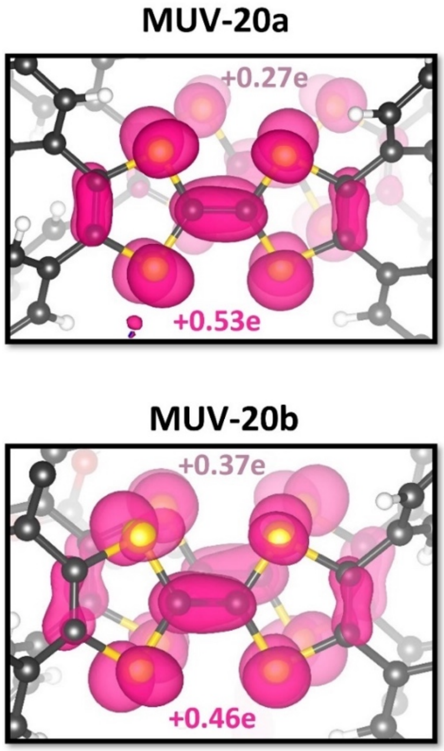

Figure S64. Unpaired-electron spin density calculated at the HSE06 level for half-protonated (50% fully protonated TTFTBs in the unit cell) **MUV-20a** and **MUV-20b** represented with isovalue contours of 0.008 au. The charge accumulation increase (Δq) for the TTF core with respect to isolated, fully protonated TTFTB ligand is indicated.

10. References

(1) Nowell, H.; Barnett, S. A.; Christensen, K. E.; Teat, S. J.; Allan, D. R. I19, the Small-Molecule Single-Crystal Diffraction Beamline at Diamond Light Source. *J. Synchrotron Radiat.* **2012**, *19* (3), 435–441. https://doi.org/10.1107/S0909049512008801.

(2) Sheldrick, G. M. Crystal Structure Refinement with SHELXL. *Acta Crystallogr. Sect. C Struct. Chem.* **2015**, *71* (Md), 3–8. https://doi.org/10.1107/S2053229614024218.

(3) Dolomanov, O. V.; Bourhis, L. J.; Gildea, R. J.; Howard, J. A. K.; Puschmann, H. OLEX2: A Complete Structure Solution, Refinement and Analysis Program. *J. Appl. Crystallogr.* **2009**, *42* (2), 339–341. https://doi.org/10.1107/S0021889808042726.

(4) Ono, S. Application of Ursell and Mayer's Treatment for Imperfect Gases to Adsorption. *J. Chem. Phys.* **1950**, *18* (3), 397. https://doi.org/10.1063/1.1747647.

(5) Hill, T. L. Statistical Mechanics of Adsorption. VI. Localized Unimolecular Adsorption on a Heterogeneous Surface. *J. Chem. Phys.* **1949**, *17* (9), 762–771. https://doi.org/10.1063/1.1747397.

(6) Blum, V.; Gehrke, R.; Hanke, F.; Havu, P.; Havu, V.; Ren, X.; Reuter, K.; Scheffler, M.

Ab Initio Molecular Simulations with Numeric Atom-Centered Orbitals. *Comput. Phys. Commun.* **2009**, *180* (11), 2175–2196. https://doi.org/10.1016/j.cpc.2009.06.022.

(7) Havu, V.; Blum, V.; Havu, P.; Scheffler, M. Efficient O (N) Integration for All-Electron Electronic Structure Calculation Using Numeric Basis Functions. *J. Comput. Phys.* **2009**, *228* (22), 8367–8379. https://doi.org/10.1016/j.jcp.2009.08.008.

(8) Ren, X.; Rinke, P.; Blum, V.; Wieferink, J.; Tkatchenko, A.; Sanfilippo, A.; Reuter, K.; Scheffler, M. Resolution-of-Identity Approach to Hartree-Fock, Hybrid Density Functionals, RPA, MP2 and GW with Numeric Atom-Centered Orbital Basis Functions. *New J. Phys.* **2012**, *14*. https://doi.org/10.1088/1367-2630/14/5/053020.

(9) Perdew, J. P.; Ruzsinszky, A.; Csonka, G. I.; Vydrov, O. A.; Scuseria, G. E.; Constantin, L. A.; Zhou, X.; Burke, K. Restoring the Density-Gradient Expansion for Exchange in Solids and Surfaces. *Phys. Rev. Lett.* **2008**, *100* (13), 1–4. https://doi.org/10.1103/PhysRevLett.100.136406.

(10) Tkatchenko, A.; Scheffler, M. Accurate Molecular van Der Waals Interactions from Ground-State Electron Density and Free-Atom Reference Data. *Phys. Rev. Lett.* **2009**, *102* (7), 6–9. https://doi.org/10.1103/PhysRevLett.102.073005.

(11) "Gaussian 16, Revision A.03, Frisch, M. J.; Trucks, G. W.; Schlegel, H. B.; Scuseria, G. E.; Robb, M. A.; Cheeseman, J. R.; Scalmani, G.; Barone, V.; Petersson, G. A.; Nakatsuji, H.; Li, X.; Caricato, M.; Marenich, A. V.; Bloino, J.; Janesko, B. G.; Gomperts, R.; Mennucci, B.; Hratchian, H. P.; Ortiz, J. V.; Izmaylov, A. F.; Sonnenberg, J. L.; Williams-Young, D.; Ding, F.; Lipparini, F.; Egidi, F.; Goings, J.; Peng, B.; Petrone, A.; Henderson, T.; Ranasinghe, D.; Zakrzewski, V. G.; Gao, J.; Rega, N.; Zheng, G.; Liang, W.; Hada, M.; Ehara, M.; Toyota, K.; Fukuda, R.; Hasegawa, J.; Ishida, M.; Nakajima, T.; Honda, Y.; Kitao, O.; Nakai, H.; Vreven, T.; Throssell, K.; Montgomery, J. A., Jr.; Peralta, J. E.; Ogliaro, F.; Bearpark, M. J.; Heyd, J. J.; Brothers, E. N.; Kudin, K. N.; Staroverov, V. N.; Keith, T. A.; Kobayashi, R.; Normand, J.; Raghavachari, K.; Rendell, A. P.; Burant, J. C.; Iyengar, S. S.; Tomasi, J.; Cossi, M.; Millam, J. M.; Klene, M.; Adamo, C.; Cammi, R.; Ochterski, J. W.; Martin, R. L.; Morokuma, K.; Farkas, O.; Foresman, J. B.; Fox, D. J. Gaussian, Inc., Wallingford CT, 2016."

(12) Rassolov, V. A.; Ratner, M. A.; Pople, J. A.; Redfern, P. C.; Curtiss, L. A. 6-31G* Basis Set for Third-Row Atoms. *J. Comput. Chem.* **2001**, *22* (9), 976–984. https://doi.org/10.1002/jcc.1058.

(13) Marcus, R. A. On the Theory of Oxidation-Reduction Reactions Involving Electron Transfer. I. *J. Chem. Phys.* **1956**, *24* (5), 966–978. https://doi.org/10.1063/1.1742723.

(14) Perdew, J. P.; Burke, K.; Ernzerhof, M. Generalized Gradient Approximation Made Simple. *Phys. Rev. Lett.* **1996**, *77* (18), 3865–3868. https://doi.org/10.1103/PhysRevLett.77.3865.

(15) Momma, K.; Izumi, F. VESTA 3 for Three-Dimensional Visualization of Crystal, Volumetric and Morphology Data. *J. Appl. Crystallogr.* **2011**, *44* (6), 1272–1276. https://doi.org/10.1107/S0021889811038970.
